# Digital assistive technologies for community-dwelling people with dementia: A systematic review of systematic reviews by the INTERDEM AI & assistive technology taskforce

**DOI:** 10.1177/20552076251362353

**Published:** 2025-08-03

**Authors:** David Neal, Michael P Craven, Jane Cross, Shirley Evans, Christopher Fox, Laila Oksnebjerg, Isabel Alexandre, Aidin Aryankhesal, Arlene Astell, Ahmet Begde, Annabel Ditton, Thomas Engelsma, Rikke Gregersen, Pascale Heins, Eef Hogervorst, Aysegul Humeyra Kafadar, Jackie Poos, Louise Robinson, Duygu Sezgin, Hanneke JA Smaling, Dorota Szczesniak, Josephine Rose Orejana Tan, Marjolein de Vugt, Franka JM Meiland

**Affiliations:** 1 Department of Medical Informatics, Amsterdam UMC, Amsterdam, The Netherlands; 2 NIHR MindTech HealthTech Research Centre & Centre for Dementia, Institute of Mental Health, University of Nottingham Innovation Park, Nottingham UK; 3 Human Factors Research Group, Faculty of Engineering, 6123 University of Nottingham, Nottingham, UK; 4 School of Health Sciences, Faculty of Medicine and Health Sciences, 6106 University of East Anglia, Norwich, UK; 5 The Association for Dementia Studies, 8709 University of Worcester, Worcester UK; 6 NIHR HealthTech Research Centre in Sustainable Innovation, 3286 University of Exeter, Exeter, UK; 7 NIHR HealthTech Research Centre National Coordinating Centre, 7315 University of Sheffield, Sheffield UK; 8 School of Health and Community Sciences, Medical School, 3286 University of Exeter, Exeter, UK; 9 Department of Psychology, 4321 University of Copenhagen, Copenhagen, Denmark; 10 Department of Information Science and Technology, Instituto Universitário de Lisboa (ISCTE-IUL), Lisboa, Portugal; 11 Instituto de Telecomunicações, Lisboa, Portugal; 12 School of Health Sciences, Faculty of Medicine and Health Sciences, 6106 University of East Anglia, Norwich, UK; 13 Psychology Department, Faculty of Health & Life Sciences, 5995 Northumbria University, Newcastle upon Tyne, UK; 14 Department of Psychiatry, 6396 University of Oxford, Oxford, UK; 15 School of Health and Community Sciences, Medical School, 3286 University of Exeter, Exeter, UK; 16 eHealth Living & Learning Lab Amsterdam, Department of Medical Informatics, Amsterdam UMC, Amsterdam, The Netherlands; 17 Amsterdam Public Health, Digital Health, Amsterdam, Netherlands; 18 Amsterdam Public Health, Aging & Later Life, Amsterdam, Netherlands; 19 Research Centre for Activity and Prevention, 99407 VIA University College, Aarhus N, Denmark; 20 Alzheimer Centrum Limburg, Department of Psychiatry and Neuropsychology, 5211 Maastricht University, Maastricht, the Netherlands; 21 School of Sports Exercise and Health Sciences, 5156 Loughborough University, Epinal Way, Loughborough, UK; 22 Academic Unit of Mental Health and Clinical Neuroscience, School of Medicine, 6123 University of Nottingham, Nottingham, UK; 23 Alzheimercenter Erasmus MC, Department of Neurology, Erasmus MC University Medical Center, Rotterdam, The Netherlands; 24 Population Health Sciences Institute, Faculty of Medicine, 5994 Newcastle University, Newcastle upon Tyne, UK; 25 School of Nursing and Midwifery, 8799 University of Galway, Galway, Ireland; 26 Department of Public Health and Primary Care, 4501 Leiden University Medical Center, Leiden, The Netherlands; 27 University Network of the Care Sector Zuid-Holland, 4501 Leiden University Medical Center, Leiden, The Netherlands; 28 Department of Psychiatry, 49550 Wroclaw Medical University, Wroclaw, Poland; 29 Department of Psychiatry, Amsterdam Public Health Research Institute, Amsterdam UMC, location Vrije Universiteit, Amsterdam, The Netherlands; 30 Alzheimer Centrum Limburg, Department of Psychiatry and Neuropsychology, Mental Health and Neurosciences Research Institute, Faculty of Health Medicine and Life Sciences, 5211 Maastricht University, Maastricht, The Netherlands; 31 Department of Medicine for Older People, Amsterdam UMC, Amsterdam, The Netherlands; 32 Amsterdam Public Health Research Institute, Aging & Later life, Amsterdam, The Netherlands

**Keywords:** Dementia, assistive technology, digital health, artificial intelligence, systematic review, development, usability, implementation, cost-effectiveness, ethics

## Abstract

**Introduction:**

The use of digital assistive technologies by and for people living with dementia is promising for supporting social health and advocated as a partial solution to growing prevalence worldwide. A state-of-the-art position paper published in 2017 identified challenges regarding digital assistive technologies, around five themes: development, usability, (cost-)effectiveness, implementation and ethics. This systematic review summarizes progress on the challenges found in 2017, and persisting or emerging challenges.

**Methods:**

A systematic review of systematic reviews was conducted, focused on studies published after 2016. The inclusion criteria required that the target group included, at least in part, people with dementia living in the community and that the technologies aimed to support social health. For the five themes, literature searches were conducted in Medline, CINAHL, PsycINFO, and Embase databases.

**Results:**

A total of 112 reviews were included, covering various applications such as smart homes, care robots, exergaming and everyday technologies. No applications of artificial intelligence were included. The challenges included personalization of applications (development); limited use of standardized methods (usability); insufficient quantity and quality of randomized controlled trials (cost-effectiveness); overly high expectations of assistive technologies (implementation); and the need for more equitable access to technologies (ethics).

**Conclusion:**

Much research has been conducted since the 2017 state of the art position paper. While some challenges identified at that time remain relevant, others have been addressed, and new challenges have emerged. Future research should prioritize emerging artificial intelligence applications; the development of integrated assistive technologies; evaluation using robust methods and meaningful outcomes; and the promotion of more accessible and inclusive technologies.

## Introduction

Dementia is a major global health priority, with prevalence estimated at more than 50 million, which may double by 2050.^1^ The high prevalence and complex nature of dementia lead to high economic costs, with the global annual cost of dementia care amounting to US $1.33 trillion.^2^ There is currently no cure for dementia and new medications for Alzheimer's disease at best, produce small and time-limited effects on the rate of disease progression, for a subgroup of eligible people.^3,4^ Early diagnosis and availability of personalized care and support therefore remain essential for people living with dementia to enjoy the best possible quality of life and good social health (a positive health state arising from the ability manage independently in daily life, engage in meaningful and pleasurable activities and maintain social networks, and meet societal obligations).^5,6^ As part of providing effective personalized care and support, many global and national dementia policies emphasize the use of digital assistive technologies – by people with dementia themselves, family carers and health and social care professionals – especially to support those living at home, supported primarily by friends and family.^7^

The INTERDEM AI & Assistive Technology taskforce defines digital assistive technology as “any item, piece of equipment, product or system driven by electronics, whether acquired commercially, off-the-shelf, modified or customized, that is used to help people living with dementia in dealing with the consequences of dementia”^8^^(p.2)^. This includes digital technologies, such as televisions or smartphones, not purposely designed for people with dementia, but adapted to their needs, and few such technologies are provided systematically through health and social care. Many technologies are funded by people with dementia themselves or by family carers. People with dementia and their supporters experience the need for information about the condition, memory and cognitive support, engaging in social or meaningful activities and managing psychological distress,^9^ all of which may be helped by digital assistive technologies. Technologies primarily used by people with dementia may also indirectly benefit their supporters, such as family or informal carers. For example, hybrid exergaming interventions that combine virtual reality and physical activity have been shown to improve cognition in the short term and social functioning of people with dementia and competence of family carers.^10^ Interventions that support people to find and use software applications have been shown to increase capacity and reduce burden on informal carers.^11^ Many people living with dementia wish to live independently at home for as long as possible,^12^ suggesting a particularly important role for digital assistive technologies that support quality of life and social health of community-dwelling people with dementia.

Despite the promise of digital assistive technologies for supporting social health, a 2017 position paper from INTERDEM^8,13^ (an interdisciplinary network supporting pan-European research on psychosocial interventions in dementia) identified important challenges related to their development, usability, (cost-)effectiveness, deployment (or implementation) and ethics.^8^ In development, challenges included accounting for individual variations in needs, abilities and preferences, developing technologies that address emotional needs alongside functional needs, and integrating digital assistive technologies into the physical environment and organizational health care processes. In the evaluation of usability, a fundamental challenge was a lack of research on this topic. Evaluating (cost-)effectiveness was challenging due to a lack of methodologically sound comparative studies such as randomized controlled trials (RCTs). A related implementation challenge was all stakeholders’ (i.e., technology users, funders and professionals) lack of access to trusted sources of information about what works. Another implementation challenge was inadequate infrastructure, such as internet or network connections, service provision, data storage, system integrity, privacy and security. Additionally, ethical issues related to users’ lack of understanding of technology and possible inability to provide informed consent to its use required more attention in evaluation studies. A number of actions were proposed, primarily for researchers, to address the challenges identified, such as including people with dementia in technology design and performing more studies on cost-effectiveness.^8(p.12)^

Since the publication of the first INTERDEM position paper, the field of digital assistive technology for people with dementia has continued to develop rapidly, partly driven by adaptations during the COVID-19 pandemic,^14^ and also intensifying interest in the capability of artificial intelligence. In light of these developments, the goal of this literature review was to address three research questions:

1. To what extent have the challenges identified in the previous INTERDEM position paper been addressed?
2. What new or emerging challenges need to be addressed going forward?
3. In light of this, what are the current priorities for research and practice?

## Methods

A systematic review of systematic reviews was performed by members of the INTERDEM AI & Assistive Technology Taskforce. PRISMA guidelines were followed in designing, executing and reporting on the study, although the review protocol was not pre-registered.^15^ A review of reviews provides a broad, yet detailed understanding of the current landscape, informing future research and practice in this rapidly evolving area.^16^

The inclusion criteria were: 1) the study was a quantitative or mixed methods systematic review; 2) the review was written in English; 3) the review was published in, or after, 2016 (the date of searches run in relation to the 2017 position paper, which this study is based on); 4) the review reported (at least partly) on persons with dementia living in the community; 5) included at least one study concerning a digital assistive technology; and 6) at least some of the technologies identified by the review aimed to support one or more of: self-management or (basic or instrumental) activities of daily living, engagement in meaningful and pleasurable activities or delivery of health or social care if this involved the person with dementia using technology. Protocols and any other form of review than a systematic review were excluded.

Parallel literature searches were conducted on each of the predefined topics (development, usability, (cost-)effectiveness, implementation and ethics) in Medline, CINAHL, PsycINFO and Embase databases. Searches for four themes were conducted in June 2024 and for the development theme in September 2024. All of the searches shared common terms related to dementia, digital assistive technology and reviews. For each search, further terms related to the specific theme were then added to these common terms with the AND operator. See Supplementary File 1 for the full search strings for each theme.

Literature search results were imported into the software Rayyan.^17^ Following de-duplication, titles of the results from each search were initially screened by a single author and clearly irrelevant records were excluded. Subsequently, titles and abstracts were screened, and results that did not meet the inclusion criteria were excluded. For the implementation theme, two reviewers screened 10% of the results, after which a single reviewer completed the process, as inter-rater reliability exceeded 90% and senior researcher capacity on this theme was limited. For all other themes, titles and abstracts were screened by two reviewers working independently. Disagreements were resolved through discussion with a senior researcher to reach consensus. Reviews that met the inclusion criteria were included for analysis according to each theme. Any given review could be included and analyzed from the perspective of multiple themes.

Relevant data from each included review were extracted by a single reviewer using a form adapted from a tool used for the 2017 position paper, and data were checked by a second reviewer. For all themes, the following information was recorded: nature of the technologies investigated, the total number of studies and participants included per review, and any challenges and research priorities discussed by the review authors. For the (cost-)effectiveness theme, outcomes, method of synthesis, reported overall risk of bias of included studies and any reported meta-analysis results were recorded. Formal assessment of the quality of the reviews themselves was not undertaken. Data extraction was performed by a single investigator and then checked by a senior investigator.

The technologies described in the reviews were heterogeneously defined (e.g., based on underlying hardware or software; specific applications of technology; or based on the purpose for which technology was implemented). An inductive, iterative clustering process was therefore undertaken to define categories of technologies. Tabulated vote-counting (a simple numerical synthesis summing the number of relevant studies) was used to present technologies investigated in reviews included in each theme. Narrative synthesis of challenges for research and practice identified by authors of included reviews was undertaken, with comparison to challenges and priorities identified in the 2017 position paper.^8^ Priority actions for researchers and other stakeholders were then formulated by the investigators.

## Results

**Figure 1.**
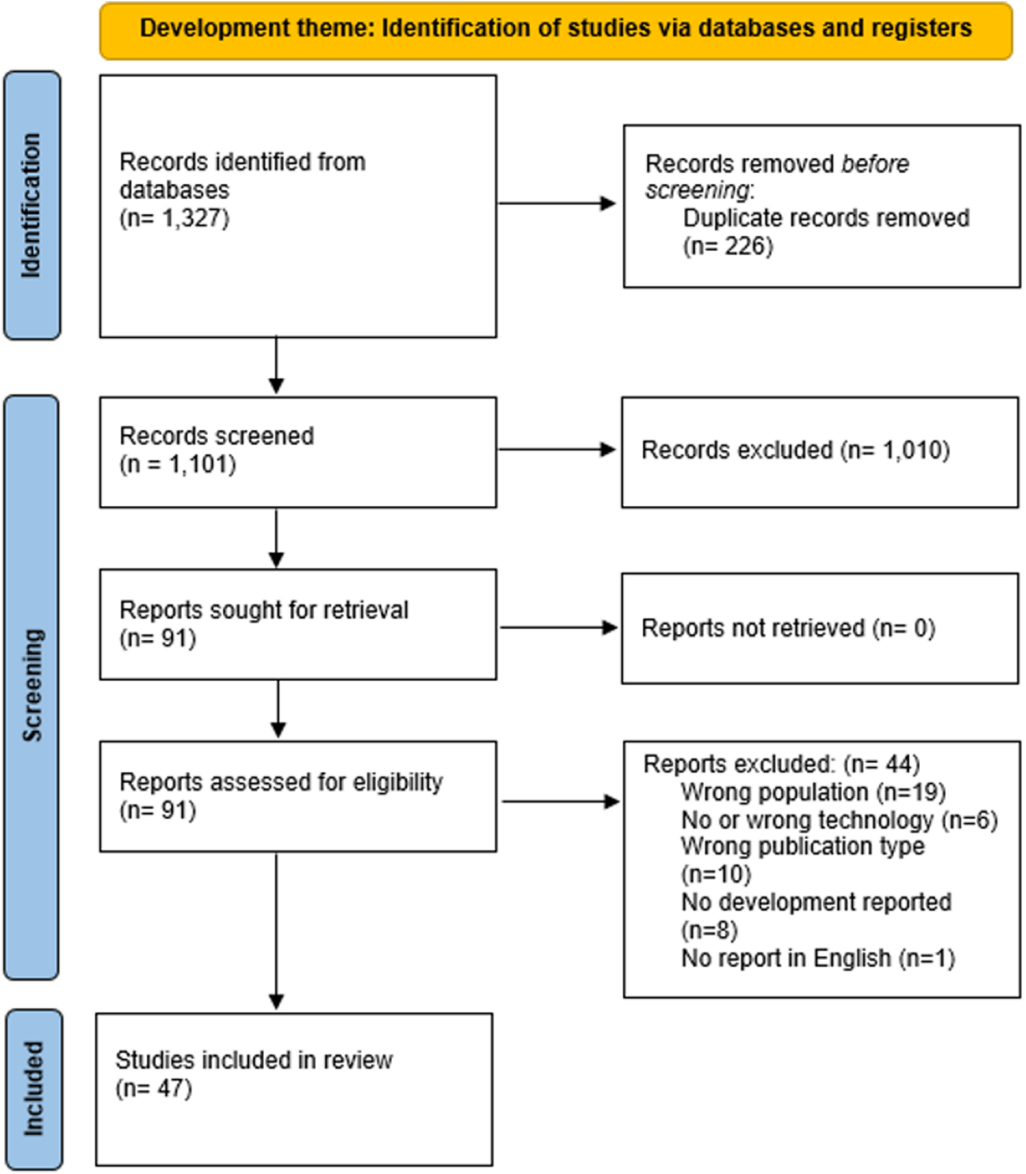
PRISMA flow diagram for the Development theme search and screening process.

For each search, the number of unique records screened, as well as the reports assessed for eligibility and included in this review, is presented in Table 1. Figure 1 illustrates the full screening process and reasons for exclusions of full reports not included, for the development theme. Similar flow diagrams depicting the screening processes for the other themes are available in Supplementary File 2. Some reviews were included in more than one theme. Across all themes, this systematic review of systematic reviews included 112 unique systematic reviews in total.

**Table 1.** Number of records and full reports screened and studies included within each theme based on the respective search and screening process.

Theme	Unique records screened (n)	Full reports screened (n)	Studies included (n)
Development	1101	91	47^14,18–63^
Usability	284	57	39^26,28,29,32,38,39,43,44,56,58–60,63–89^
(Cost-)effectiveness	1113	60	35^10,19,23,25,30,35,41,45,46,49,53,57,59,61,90–110^
Implementation	1399	254	33^18,22,28,29,33,44,45,47,56,68,69,71,72,86,91,100,102,110–125^
Ethics	1215	26	14^38,39,56,60,71,76,82,89,91,110,119,126–128^

Data extracted from reviews within each theme are provided in Supplementary File 3. Table 2 shows the digital technologies addressed in the included systematic reviews by theme, along with the number of reviews within each theme that focused on each technology. Since many reviews addressed more than one digital technology, the total number of reviews per technology within each theme exceeds the number of systematic reviews included per theme. No reviews were focused on applications of artificial intelligence.

**Table 2.** Type and number of digital technologies investigated in studies included within each theme.

	Technology						
Theme	AAL, smart home & sensor-based (n studies)	Care robots & conversational agents (n studies)	Exergame & VR (n studies)	Telecare (n studies)	Wearable and tracking devices (n studies)	Screen- based software applications (n studies)	Everyday technologies (n studies)
Development	15	13	12	6	7	18	7
Usability	6	16	3	11	6	10	6
(Cost-) effectiveness	4	7	14	4	2	13	0
Implementation	8	8	11	12	11	16	4
Ethics	6	3	3	3	5	7	1

AAL: ambient and assisted living; VR: virtual reality.

## Narrative synthesis of included reviews

### Development

Of the 47 included reviews, eight included at least one meta-analysis,^24,28,30,46,52–54,61,63^ with the rest employing narrative synthesis. As shown in Table 2, there were more reviews on the development of software apps, ambient and assisted living technologies, robots, conversational agents and VR technologies, than on telecare, (wearable) tracking devices or everyday technologies.

Many reviews conclude that appropriate inclusion of people with dementia in the design process remains a challenge. For example, many developers seemed to include people with dementia in the development only as informants rather than co-creators, and methods such as iterative consultation and prototype evaluation were underused.^55^ This is particularly pertinent for GPS tracking technologies and dementia-specific apps, leading to mismatched designs and inadequate attention to security and usability.^32,55^ Moreover, people with dementia were often considered a homogeneous group for the purposes of inclusion in co-design, despite variation in needs by dementia type (e.g., Alzheimer's Disease, Parkinson's dementia) and stage. Other challenges to successful technology development detailed in the included reviews related to technical aspects of making applications personalizable, high development costs and concerns amongst developers regarding weak evidence for acceptance and effectiveness.^63^ Even when development prioritized design aspects such as simplicity, personalization and interactivity, issues with technology adoption seemed to persist, especially for those with sensory impairments.^56,59^

### Usability

The inclusion of 39 reviews, collectively addressing applications of a wide range of different digital technologies, shows that one challenge identified in the 2017 position paper has been addressed, namely that there were then very few reviews to include. There has been an increase in interest in usability.

Regarding further challenges, authors of the included reviews continued to appeal for more involvement of technology end-users in design and development of technologies, to ensure usability of technologies.^26,32,38,68,71,84^ However, literature now goes further and deeper into the conceptual framework for usability, and the process of conducting usability and user experience (UX) studies. For example, several authors emphasized the importance of technologies being not only functional but also pleasurable and meaningful, recognizing UX and user satisfaction as key components of usability.^44,56,63,69,70,81,86^ A wide range of designs and methods were described but there is an ongoing challenge of poor utilization of standardized methods and instruments, and over-reliance on the ad hoc use of unvalidated interview guides and instruments that are insufficiently grounded in human factors engineering or human–computer interaction theory.^60,67,70,79^ A more standardized approach would facilitate A/B testing of similar solutions and would facilitate benchmarking and quantitative comparisons between solutions rather than qualitative descriptions of single products.^26,63,76^

In terms of defining and understanding the *user* in the context of usability, the timeliness and fit of technologies within the context of the dementia journey – the experience of needs changing as the disease progresses – were highlighted by authors as being under-addressed, with negative consequences for technology acceptance.^28,58,68,84^ Additionally, there was limited mention of equity, diversity and inclusion in usability research, particularly in terms of including representative samples of technology end users.^71,74,80^

### (Cost-)effectiveness

Of the 35 included reviews, 15 included at least one meta-analysis,^30,46,53,90–93,95,97,98,101,104,105,107,108^ with the remainder employing narrative synthesis. Three reviews additionally sought evidence on the cost-effectiveness of assistive technologies in dementia care,^10,94,96^ but only one of these eventually identified and included any relevant cost-effectiveness studies.^96^ Amongst the meta-analyses, three reviews claimed low overall risk of bias of the included studies,^90,91,107^ the rest noted moderate, high or unclear overall risk of bias.

Little research was found by review authors on the impact of technologies on delivery of health or social care, with minimal evidence for effectiveness and no indication of cost-effectiveness. Overall, most of the challenges identified in 2017 review remained: authors of several of the now-included reviews continued to note both insufficient quantity and quality of RCTs, and methodologically sound research in general^23,45,59,91,97,102^; lack of an evidence-based consensus on acceptable alternative designs to RCTs^45,98^; and on adequate and valid outcome measures^19,30,45,46,49,61,93,97,99,101,109^; and, in line with our search results, some authors noted an absence of cost-effectiveness studies.^10,91,94,96^ Additionally, new challenges were identified, including concerns about the number of studies including wealthy and educated segments of populations in the global north,^30,103^ thus producing questions regarding real-world applicability of evidence from trials.^23,59^

### Implementation

Authors of some recent reviews considered that frustration arising from unrealistically high expectations of technology was a barrier to adoption and implementation. This contrasts with one of the challenges identified in 2017, that the perceived potential of digital assistive technologies was often unclear to users. This includes expectations around the ease of use and integration into daily life and health care workflows, as well as expectations in terms of the functional or interactive capabilities of, for example, robots.^56,129^

Many other challenges identified in 2017 remain, and research in the intervening period has served to further specify the nature of these challenges. For example, where previously review authors noted poor usability of technologies as a barrier to implementation, this has now been further refined as due to unreliable or poor quality technologies, suggesting insufficient testing and validation before implementation.^18,29,44,45,47,56,68,71,72,86,100,102,111,116,117,119,121,123^ Poor and variable access to technologies has been further specified as relating to high out-of-pocket costs and lack of essential infrastructure such as home internet connections.^18,33,56,68,110,111,114,115,117,119,122^ The poor digital literacy of many people living with dementia, their supporters and care professionals, remains a challenge, and recent reviews also specifically highlighted a lack of training or implementation support for users of technologies currently on the market.^45,68,71,114,121,124^ Problems with reimbursement for developers of novel technologies were already noted in 2017, and more recently, authors also expressed that new regulatory burden on developers create a risk-averse and stifled innovation landscape that contributes to higher out-of-pocket costs.^91,110,119,125^ In 2017, authors reported concerns about privacy weighed on implementation and adoption, and more recent reviews highlighted additionally, a broader understanding of and concern about a wide range of ethical issues in relation to assistive technologies in dementia care as barriers to implementation.^110,111,113,114,119,125^ Finally, adverse consequences of assistive technologies such as stigma and depersonalization remains a concern.^18,47,56,68,69,72,100,113,115,122–124^ It is now recognized that adverse consequences can also negatively impact on informal caregiver well-being.^45,119^

### Ethics

The challenges identified in 2017 remain: authors are appealing for more research into the ethical implications of digital assistive technologies,^38,60^ and highlighting difficulties in obtaining truly informed consent from participants with poor digital literacy.^39,126,127^ However, previously the challenge was around lack of public awareness of ethical issues, now more authors of new reviews cited high awareness of and sensitivity to ethical issues amongst people living with dementia, their supporters and health and care professionals. In particular, increased attention was paid in recent reviews to the awareness of technology users of challenges regarding data privacy,^60,76,91,119,127^ balancing autonomy and safety for people living with dementia,^127,128^ equitable access to technology for all who may benefit from it,^71,110,127^ and the risk that dementia-specific assistive technologies may be stigmatizing.^56,60^ The use of AI is creating new concerns in particular over for example security and validity.^130^

## Discussion

This review is based upon a previous position paper, identifying challenges with the development, usability, (cost-)effectiveness, implementation and ethics of digital assistive technologies for community-dwelling people living with dementia. We included 112 systematic reviews, addressing a broad range of technologies and applications.

Challenges identified from the included reviews and our recommendations are listed in Table 3.

**Table 3.** Summary of main challenges identified and recommended actions for each theme.

Identified challenges	Recommended actions
Development	
inclusion of people with dementia in design processes financial challenges for developers when promoting adoption and implementation	use and report on robust co-design methodologies taking into account which methods work best for each type of technology facilitate tailored technologies to personalized and diverse needs and preferences, considering the role of adaptive designs and the use of AI to re-align with the needs and capabilities of people living with dementia
Usability	
need for more advanced and standardized designs and methodologies	use standardized quantitative and qualitative approaches, based on theories from human factors engineering (HFE) and human-computer interaction (HCI). establish interdisciplinary collaborations funding organizations should stress this interdisciplinary collaborations and the comparative and longitudinal usability studies policy makers and those purchasing digital assistive technologies should demand robust evidence of usability
Effectiveness and cost-effectiveness	
a dearth of high-quality primary research into the effectiveness, and especially the cost-effectiveness	researchers should adhere to high methodological standards RCTs have excellent explanatory power, whilst well executed. This should be supported by adequate funding alternative robust evaluative frameworks are needed relevant outcomes should be targeted on: biological, psychological and social; personalized for people with dementia and informal caregivers; cost-effectiveness from a societal perspective
Implementation	
unrealistically high expectations of technology unreliable and poor quality technologies lack of training or implementation support for users	realistic and evidence based expectations regarding benefits of technology should be communicated a national, easy access database with information about different digital assistive technology applications, can be helpful in choosing technologies, like the Dutch organization Vilans^135^ or the Alzheimer's Society in the UK.^136^ use evidence-based implementation frameworks when implementing technologies in care collaborations between technology developers, policy makers and funders are needed to anticipate on reimbursement and standardized purchasing procedures adequate integration of digital skills into curricula for health and social care professionals in training are needed
Ethical issues	
lack of inclusive research into ethical issues in this field concerns by researchers and technology users, regarding: o data privacy o equitable access to technologies o stigma related to dementia specific technologies o balancing autonomy and safety	more research on ethical implications of digital assistive technologies a balance between autonomy and safety must be continuously sought throughout the technology and research cycle together with people with dementia and their supporters specific ethical challenges related to AI and dementia should be addressed,^137^ given risks of, for example, (a) poor training of AI based on selective data leading to biased systems, (b) privacy and data security concerns, (c) overreliance on AI further reducing autonomy, (d) inequality through affordability and device availability, (e) need for user involvement in design (f) security and safeguarding systems regulatory systems need to also keep up with AI developments to ensure safety.^138^

Besides challenges identified, there were also positive developments since the previous position paper. The large number of included reviews regarding the topic usability demonstrates increasing interest and an understanding of this topic. This is also cited as an important factor impacting on implementation and more research has been published, specifying how other factors impacting implementation. Furthermore, results showed an increased awareness of ethical issues surrounding dementia and technology, particularly regarding data privacy. This could be related to the introduction of the EU General Data Protection Regulation in 2018.^131^

The large number of results identified highlights the increasing amount of research interest and activity in this field. However, the relatively small proportion of reviews included under the criteria seems to suggest little of this activity has productively advanced the field in line with the recommendations set-out in INTERDEM's 2017 position paper.^8^ It may be that adequate primary literature exists but reviewers have not prioritized the themes identified by INTERDEM. However, if reviews are broadly representative of underlying primary literature, the mismatch between INTERDEM's recommendations and research activity is concerning. The previous review and INTERDEM position paper have been well-disseminated, as demonstrated by the paper's field citation index, 61.85.^8^ This implies that researchers have been well aware of the challenges faced and priorities for future research and practice, meaning that structural barriers (such as lack of funding) or misaligned incentives (e.g., between technology developers bringing technologies to market, researchers trying to build an evidence base, and funders trying to limit spending) may therefore be responsible for any shortcoming in addressing these research priorities. For example, factors contributing to the ongoing absence of large-scale RCTs may be a lack of funding for these studies, or the criticism of this design's limitations when applied to digital technologies that may discourage funders from funding such studies or researchers from carrying them out.^132,133^ The COVID-19 pandemic, whilst a driver of accelerated uptake of digital assistive technologies,^14^ may have also hampered the ability to conduct such studies. Lack of RCT-level evidence may explain the notable absence of recommendations related to specific digital assistive technologies, identified by a recent review of European dementia guidelines.^134^ The lack of evidence-based consensus on appropriate alternative designs to RCTs may be due to lack of coordination and alignment between stakeholders. Particularly where there may be conflicting interests, between payers in health and social care, developers of technology, health and care professionals, researchers and people living with dementia and their supporters, strong leadership – for example, from national and international dementia organizations, and policy makers – will be necessary to achieve progress. In the included reviews and underlying primary literature, a wide-range of heterogeneous digital assistive technologies was addressed, although it was remarkable that none concerned applications of AI.

Given the results from all themes considered in this study, we identify three cross-cutting priorities for stakeholders (researchers, policy-makers, professionals, people with lived experience, technology developers and other stakeholders):

1. Increase the focus of research on innovative artificial intelligence solutions that may help improve personalization, accessibility, efficiency and effectiveness of digital assistive technologies for people being diagnosed and living with dementia, their supporters and health and care professionals.
2. Move away from researching, purchasing and implementing isolated digital assistive technologies, move towards integrated, person-centered digital health ecosystems and multi-level interventions; within this framework, establish consensus on meaningful outcome measures and appropriate evaluation methods that go beyond RCTs.
3. Reduce inequities in access and digital literacy, and strive for accessible and inclusive technology by engaging more diverse populations throughout design, research and implementation, and by making personalized training and implementation support integral components of digital assistive technologies. This includes people from less advantaged areas.

### Strengths and limitations

This review stands out for the broad range of topics addressed and the inclusion of a large number of reviews addressing an extensive number of technologies, research methods and settings. In addition, it builds on a previous position paper, ensuring the continuity of knowledge. Nonetheless, some limitations are identified. Only reviews in English were included, which may have limited the comprehensiveness of results. However, the primary literature underlying the systematic reviews included in this study included articles not written in English. Another limitation of this study was that the included systematic reviews did not undergo formal quality assessment. Tools such as AMSTAR 2 are available for assessing the quality of systematic reviews,^139^ and such assessments would be particularly relevant when the efficacy of an intervention was the primary focus of the study. However, as this was not the primary goal of this review and in light of the limited resources available for this unfunded research, quality assessment of the 112 included systematic reviews was not undertaken*.*^139^ Finally, there are two general limitations of reviews of reviews that are important. The focus is on technologies in research studies that may not be representative of technologies in practice, and the combined time delay between primary research and results being published, and included in systematic reviews means that results may not represent the most recent developments. To overcome such limitations, a Delphi study is being conducted, to capture the current challenges and priorities as experienced by people living with dementia and their supporters, health and care professionals and technology developers. The results of both studies will be consolidated and integrated into a white paper in 2025, to provide comprehensive insight into the current and future state of priorities in the field of dementia.

## Conclusion

Much research has been conducted since the previous position paper in 2017 but many of the challenges to the field of digital assistive technology for the support of people with dementia remain unresolved, whilst new challenges have also emerged. Future research priorities should focus research on the development, usability, effectiveness, implementation and ethics of AI applications; integration of digital assistive technologies in digital ecosystems; usability and inclusiveness of technologies; new research methods, including evaluation methodologies and outcome measures. Aside from more research, progress in the field will require continuing and better involvement of people with dementia and their supporters, and better engagement with funders of health and social care and of research and with commercial parties developing and bringing digital assistive technologies to market. Seeking alignment of stakeholders around priorities and evidence frameworks will require international leadership from civil society and policy-makers.

## Supplemental Material

sj-docx-1-dhj-10.1177_20552076251362353 - Supplemental material for Digital assistive technologies for community-dwelling people with dementia: A systematic review of systematic reviews by the INTERDEM AI & assistive technology taskforce Supplemental material, sj-docx-1-dhj-10.1177_20552076251362353 for Digital assistive technologies for community-dwelling people with dementia: A systematic review of systematic reviews by the INTERDEM AI & assistive technology taskforce by David Neal, Michael P Craven, Jane Cross, Shirley Evans, Christopher Fox, Laila Oksnebjerg, Isabel Alexandre, Aidin Aryankhesal, Arlene Astell, Ahmet Begde, Annabel Ditton, Thomas Engelsma, Rikke Gregersen, Pascale Heins, Eef Hogervorst, Aysegul Humeyra Kafadar, Jackie Poos, Louise Robinson, Duygu Sezgin, Hanneke JA Smaling, Dorota Szczesniak, Josephine Rose Orejana Tan, Marjolein de Vugt and Franka JM Meiland in DIGITAL HEALTH

sj-docx-2-dhj-10.1177_20552076251362353 - Supplemental material for Digital assistive technologies for community-dwelling people with dementia: A systematic review of systematic reviews by the INTERDEM AI & assistive technology taskforce Supplemental material, sj-docx-2-dhj-10.1177_20552076251362353 for Digital assistive technologies for community-dwelling people with dementia: A systematic review of systematic reviews by the INTERDEM AI & assistive technology taskforce by David Neal, Michael P Craven, Jane Cross, Shirley Evans, Christopher Fox, Laila Oksnebjerg, Isabel Alexandre, Aidin Aryankhesal, Arlene Astell, Ahmet Begde, Annabel Ditton, Thomas Engelsma, Rikke Gregersen, Pascale Heins, Eef Hogervorst, Aysegul Humeyra Kafadar, Jackie Poos, Louise Robinson, Duygu Sezgin, Hanneke JA Smaling, Dorota Szczesniak, Josephine Rose Orejana Tan, Marjolein de Vugt and Franka JM Meiland in DIGITAL HEALTH

sj-docx-3-dhj-10.1177_20552076251362353 - Supplemental material for Digital assistive technologies for community-dwelling people with dementia: A systematic review of systematic reviews by the INTERDEM AI & assistive technology taskforce Supplemental material, sj-docx-3-dhj-10.1177_20552076251362353 for Digital assistive technologies for community-dwelling people with dementia: A systematic review of systematic reviews by the INTERDEM AI & assistive technology taskforce by David Neal, Michael P Craven, Jane Cross, Shirley Evans, Christopher Fox, Laila Oksnebjerg, Isabel Alexandre, Aidin Aryankhesal, Arlene Astell, Ahmet Begde, Annabel Ditton, Thomas Engelsma, Rikke Gregersen, Pascale Heins, Eef Hogervorst, Aysegul Humeyra Kafadar, Jackie Poos, Louise Robinson, Duygu Sezgin, Hanneke JA Smaling, Dorota Szczesniak, Josephine Rose Orejana Tan, Marjolein de Vugt and Franka JM Meiland in DIGITAL HEALTH
