# Supplementary material for: Digital assistive technologies for community-dwelling people with dementia: A systematic review of systematic reviews by the INTERDEM AI & assistive technology taskforce
Source: Digit Health. 2025 Aug 3;11:20552076251362353. doi: 10.1177/20552076251362353 (PMC12319280; doi:10.1177/20552076251362353)
Supplement: sj-docx-1-dhj-10.1177_20552076251362353 - Supplemental material for Digital assistive technologies for community-dwelling people with dementia: A systematic review of systematic reviews by the INTERDEM AI & assistive technology taskforce [file sj-docx-1-dhj-10.1177_20552076251362353.docx]

Review title: New INTERDEM position paper on assistive technology: draft priorities for research and practice 2025-2032

Draft strategy

Concepts

- Dementia
- Assistive technology
- Reviews
- Development OR user experience OR implementation OR cost-effectiveness OR ethics

Explanation

For this strategy I have combined the four concepts listed above. I have used the search terms provided and added extra that we have identified for the concepts in previous searches. I have highlighted them in blue so you can review the terms, and let me know any you feel are inappropriate and need removing.

I have drafted this in Ovid Medline (the subscription version of PubMed). This allows me to use proximity searching, to search for terms within a specified number of words away from each other. This is beneficial for line 5. I can revert the strategy back to PubMed if this is more convenient for the rest of the team.

I have used the review filter created by the Health Sciences Library at the University of Pittsburgh, an approved filter for all review types, meta-analyses, and traditional literature (non-systematic) reviews (lines 25 to 52). This searches for not just systematic reviews, but all review types that generally follow SR methods, and includes scoping reviews, overview of reviews (aka umbrella reviews), rapid reviews, narrative reviews, as well as other reviews that follow the systematic review methodology. Link: [Systematic, scoping, and similar reviews and meta-analyses - Ovid Medline Search Filters - LibGuides at Health Sciences Library System](https://hsls.libguides.com/Ovid-Medline-search-filters#s-lg-box-32342483)

1. Development searches

Ovid MEDLINE(R) ALL <1946 to May 15, 2024>

| **#** | **Search term(s)** | **Result(s)** |
| --- | --- | --- |
| 1 | exp Dementia/ | 218816 |
| 2 | (dement* or alzheimer* or lewy or CJD or JCD or Creutzfeldt or binswanger or korsakoff or frontotemporal or FTD or VaD or "pick*1 disease").tw,kf. | 331409 |
| 3 | 1 or 2 | 367708 |
| 4 | exp Self-Help Devices/ or exp Internet/ or Information Technology/ or Mobile Applications/ or exp Therapy, Computer-Assisted/ or exp Microcomputers/ or exp Wearable Electronic Devices/ or exp Telemedicine/ or exp Virtual Reality/ or Augmented Reality/ | 266971 |
| 5 | ((assistiv* or orthotic* or supportiv* or electronic* or welfare or everyday) adj2 (technolog* or device*)).tw,kf. | 29843 |
| 6 | ("information communication technolog*" or ICT or Computer-assisted or computer-based or Web-based or "assistive technolog*").tw,kf. | 107844 |
| 7 | (E-health or ehealth* or "e health*" or mhealth or m-health or "m health" or "mobile health" or etherap* or e-therap* or (electronic adj therap*)).tw,kf. | 28102 |
| 8 | ("mobile phone*" or Smartphone* or "smart phone*" or "Mobile device*").tw,kf. | 46848 |
| 9 | (Tablet or iPad* or iPhone* or "handheld device*" or handheld-device* or "handheld computer*" or handheld-computer* or microcomputer* or palmtop* or laptop* or "personal digital assist*" or PDA or "telephone application*" or touch-screen* or touchscreen* or Robot*).tw,kf. | 150554 |
| 10 | ((mobile or software or electronic) adj1 (app or apps or application)).tw,kf. | 11809 |
| 11 | (wearable adj2 (device* or technolog*)).tw,kf. | 12950 |
| 12 | ((virtual or augmented) adj2 (realit* or environment* or platform* or world*)).tw,kf. | 30858 |
| 13 | ((mixed or extended) adj2 realit*).tw,kf. | 1627 |
| 14 | (Oculus or "Google Cardboard" or "Google Glass*" or "HTC Vive" or "Holo Lens" or "Samsung Gear" or "Magic Leap" or Wevr or NextVR or Pico or Varjo).tw,kf. | 6755 |
| 15 | ("head* up display*" or "head-up display*" or "head mount* display*" or "head-mount* display*" or "head* worn display*" or "head-worn* display*" or HUD* or "holographic display*" or "smart glass*").tw,kf. | 7893 |
| 16 | (telecare or "tele care" or telemedicine* or telerehabilitation* or telepsychiatry or telepsychology or teleconsultation* or remote consultation* or "tele therap*" or tele-therap* or teletherap* or videoconferenc* or "video conferenc*" or videoconsultation* or "video consultation*").tw,kf. | 40416 |
| 17 | (artificial adj2 intelligence).tw,kf. | 58144 |
| 18 | or/4-17 | 652459 |
| 19 | 3 and 18 | 5993 |
| 20 | User-Centered Design/ | 275 |
| 21 | (design* OR develop* OR create* OR creation).tw,kf. | 8040396 |
| 22 | (participat* OR involve* OR co-design OR codesign OR user-centered OR “user centered” OR human-centered OR “human centered” OR user-participatory OR “user participatory”).tw,kf. | 3060136 |
| 23 | or/20-22 | 10080003 |
| 24 | 19 and 23 | 3556 |
| 25 | ("systematic review" or "Meta-Analysis").pt. | 361190 |
| 26 | "Systematic Reviews as Topic"/ or "meta-analysis as topic"/ | 33346 |
| 27 | ("Cochrane Database of Systematic Reviews" or evidence report technology assessment or evidence report technology assessment summary).jn. | 16944 |
| 28 | (((comprehensive or comprehensively) adj (analysis or review or reviewed)) or ((literature or scoping) adj (search or searches))).ti,ab,kf. not "narrative review".ti. | 142280 |
| 29 | (database or databases or cinahl or cochrane or embase or psycinfo or pubmed or medline or scopus or (web adj1 science) or ((bibliographic or literature) adj (review or reviews)) or ((electronic adj (database or databases)) or (databases adj3 searched))).ti,ab,kf. | 1066343 |
| 30 | (eligibility or excluded or exclusion or included or inclusion).ti,ab,kf. | 2674835 |
| 31 | 28 and 29 and 30 | 40035 |
| 32 | ((comparative adj effectiveness) and (effectiveness adj review)).ti,ab,kf. | 134 |
| 33 | ((critical adj interpretive) and ((interpretive adj review) or (interpretive adj synthesis))).ti,ab,kf. | 233 |
| 34 | ((diagnostic adj test) and ((accuracy adj review) or (accuracy adj reviews) or (accuracy adj studies) or (accuracy adj study)) and (meta-analysis or scoping or systematic)).ti,ab,kf. | 673 |
| 35 | ((evidence adj assessment) and GRADE).ti,ab,kf. | 105 |
| 36 | ((evidence adj mapping) or (evidence adj review) or (exploratory adj review) or (framework adj synthesis) or (mapping adj review)).ti,ab,kf. | 4046 |
| 37 | ((meta adj (epidemiological or ethnographic or ethnography or interpretation or narrative or review or study or synthesis or summary or theory)) or metaethnographic or metaethnography or metasynthesis).ti,ab,kf. | 4414 |
| 38 | ((methodological or methodology) adj1 review).ti,ab,kf. | 3395 |
| 39 | ((mixed adj methods) and (methods adj1 (review or synthesis))).ti,ab,kf. | 499 |
| 40 | ((narrative adj1 synthesis) or (overview adj4 reviews) or ("PRISMA" adj4 (guideline or guidelines or preferred or reporting or requirements)) or (PRISMA adj "P")).ti,ab,kf. | 36440 |
| 41 | (((prognostic or psychometric) adj1 review) or ((qualitative adj (evidence or research)) and ((evidence or research) adj synthesis))).ti,ab,kf. | 930 |
| 42 | (((rapid adj evidence) and (evidence adj assessment)) or (rapid adj realist) or (rapid adj2 (review or reviews)) or (realist adj2 (review or reviews or syntheses or synthesis))).ti,ab,kf. | 4704 |
| 43 | (((review adj economic) and (economic adj1 (evaluation or evaluations))) or ((scoping or systematic) adj2 (review or reviews or studies or study))).ti,ab,kf. | 406540 |
| 44 | ((review adj1 reviews) or ((systematic adj evidence) and (evidence adj map)) or (systematic adj2 mapping) or (systematic adj2 literature) or (systematic adj2 (Embase or Medline or PsycInfo or PubMed)) or (systematic adj2 (review or reviews)) or ((systematical or systematically) adj2 (review or reviewed reviews)) or (systematically adj identified) or (systematized adj review) or (umbrella adj (review or reviews))).ti,ab,kf. | 377159 |
| 45 | (meta adj2 (analyse or analyser or analyses or analysis or analytic or analytical or analytics or analyze or analyzed or analyzes)).ti,ab,kf. | 319366 |
| 46 | (metaanalyse or Metaanalysen or metaanalyser or metaanalyses or metaanalysis* or metaanalytic or metaanalytical or metaanalytics or metaanalyze or metaanalyzed or metaanalyzes).ti,ab,kf. | 2862 |
| 47 | "network meta-analysis"/ | 6574 |
| 48 | (network adj1 (meta or metaanalyses or metaanalysis or metaregression)).ti,ab,kf. | 11524 |
| 49 | (systematic and ((meta adj regression) or metagression)).ti,ab,kf. | 10400 |
| 50 | review.pt. | 3382598 |
| 51 | (((integrated or integrative or narrative) adj (review or reviews)) or overview or ((state adj3 art) and (art adj (review or reviews)))).ti,ab,kf. not (systematic or scoping).ti. | 277760 |
| 52 | or/25-27,31-51 | 3796010 |
| 53 | 24 and 52 | 766 |

Ovid Embase(R) ALL <1947 to May 22, 2024>

| Embase Classic+Embase <1947 to 2024 May 28> | | |
| --- | --- | --- |
|  |  |  |
| 1 | exp Dementia/ | 476169 |
| 2 | (dement* or alzheimer* or lewy or CJD or JCD or Creutzfeldt or binswanger or korsakoff or frontotemporal or FTD or VaD or "pick* disease").tw,kf. | 461135 |
| 3 | 1 or 2 | 587722 |
| 4 | exp Self Help Device/ | 3541 |
| 5 | exp Internet/ | 136324 |
| 6 | Information Technology/ | 14492 |
| 7 | Mobile Application/ | 24936 |
| 8 | exp Computer Assisted therapy/ | 15534 |
| 9 | exp Microcomputer/ | 14972 |
| 10 | exp Wearable computer/ | 11362 |
| 11 | exp telemedicine robot/ or exp telemedicine/ | 79625 |
| 12 | exp Virtual Reality/ | 30324 |
| 13 | exp Augmented Reality/ | 3434 |
| 14 | ((assistiv* or orthotic* or supportiv* or electronic* or welfare or everyday) adj2 (technolog* or device*)).tw,kf. | 34074 |
| 15 | ("information communication technolog*" or ICT or Computer-assisted or computer-based or Web-based or "assistive technolog*").tw,kf. | 142369 |
| 16 | (E-health or ehealth* or "e health*" or mhealth or m-health or "m health" or "mobile health" or etherap* or e-therap* or (electronic adj therap*)).tw,kf. | 29032 |
| 17 | ("mobile phone*" or Smartphone* or "smart phone*" or "Mobile device*").tw,kf. | 56948 |
| 18 | (Tablet or iPad* or iPhone* or "handheld device*" or handheld-device* or "handheld computer*" or handheld-computer* or microcomputer* or palmtop* or laptop* or "personal digital assist*" or PDA or "telephone application*" or touch-screen* or touchscreen* or Robot*).tw,kf. | 223771 |
| 19 | ((mobile or software or electronic) adj1 (app or apps or application)).tw,kf. | 14982 |
| 20 | (wearable adj2 (device* or technolog*)).tw,kf. | 13911 |
| 21 | ((virtual or augmented) adj2 (realit* or environment* or platform* or world*)).tw,kf. | 37777 |
| 22 | ((mixed or extended) adj2 realit*).tw,kf. | 1686 |
| 23 | (Oculus or "Google Cardboard" or "Google Glass*" or "HTC Vive" or "Holo Lens" or "Samsung Gear" or "Magic Leap" or Wevr or NextVR or Pico or Varjo).tw,kf. | 9131 |
| 24 | ("head* up display*" or "head-up display*" or "head mount* display*" or "head-mount* display*" or "head* worn display*" or "head-worn* display*" or HUD* or "holographic display*" or "smart glass*").tw,kf. | 10941 |
| 25 | (telecare or "tele care" or telemedicine* or telerehabilitation* or telepsychiatry or telepsychology or teleconsultation* or remote consultation* or "tele therap*" or tele-therap* or teletherap* or videoconferenc* or "video conferenc*" or videoconsultation* or "video consultation*").tw,kf. | 52085 |
| 26 | (artificial adj2 intelligence).tw,kf. | 67187 |
| 27 | or/4-26 | 815682 |
| 28 | 3 and 27 | 10212 |
| 29 | Exp user-centred design/ | 655 |
| 30 | (design* OR develop* OR create* OR creation OR participat* OR involve* OR co-design OR codesign OR user-centered OR “user centered” OR human-centered OR “human centered” OR user-participatory OR “user participatory”).tw,kf. | 12721881 |
| 31 | 29 or 30 | 12721900 |
| 32 | 28 and 31 | 5854 |
| 33 | ("Cochrane Database of Systematic Reviews" or evidence report technology assessment or evidence report technology assessment summary).jn. | 18098 |
| 34 | (((comprehensive or comprehensively) adj (analysis or review or reviewed)) or ((literature or scoping) adj (search or searches))).ti,ab,kf. not "narrative review".ti. | 170712 |
| 35 | (database or databases or cinahl or cochrane or embase or psycinfo or pubmed or medline or scopus or (web adj1 science) or ((bibliographic or literature) adj (review or reviews)) or ((electronic adj (database or databases)) or (databases adj3 searched))).ti,ab,kf. | 1474362 |
| 36 | (eligibility or exclude d or exclusion or included or inclusion).ti,ab,kf. | 3951278 |
| 37 | systematic review/ or "Review"/ | 3325602 |
| 38 | Meta analysis/ | 220309 |
| 39 | 34 and 35 and 36 | 49612 |
| 40 | ((comparative adj effectiveness) and (effectiveness adj review)).ti,ab,kf. | 98 |
| 41 | ((critical adj interpretive) and ((interpretive adj review) or (interpretive adj synthesis))).ti,ab,kf. | 239 |
| 42 | ((diagnostic adj test) and ((accuracy adj review) or (accuracy adj reviews) or (accuracy adj studies) or (accuracy adj study)) and (meta-analysis or scoping or systematic)).ti,ab,kf. | 795 |
| 43 | ((evidence adj assessment) and GRADE).ti,ab,kf. | 125 |
| 44 | ((evidence adj mapping) or (evidence adj review) or (exploratory adj review) or (framework adj synthesis) or (mapping adj review)).ti,ab,kf. | 4601 |
| 45 | ((meta adj (epidemiological or ethnographic or ethnography or interpretation or narrative or review or study or synthesis or summary or theory)) or metaethnographic or metaethnography or metasynthesis).ti,ab,kf. | 4859 |
| 46 | ((methodological or methodology) adj1 review).ti,ab,kf. | 3897 |
| 47 | ((mixed adj methods) and (methods adj1 (review or synthesis))).ti,ab,kf. | 496 |
| 48 | ((narrative adj1 synthesis) or (overview adj4 reviews) or ("PRISMA" adj4 (guideline or guidelines or preferred or reporting or requirements)) or (PRISMA adj "P")).ti,ab,kf. | 42452 |
| 49 | (((prognostic or psychometric) adj1 review) or ((qualitative adj (evidence or research)) and ((evidence or research) adj synthesis))).ti,ab,kf. | 1094 |
| 50 | (((rapid adj evidence) and (evidence adj assessment)) or (rapid adj realist) or (rapid adj2 (review or reviews)) or (realist adj2 (review or reviews or syntheses or synthesis))).ti,ab,kf. | 5432 |
| 51 | (((review adj economic) and (economic adj1 (evaluation or evaluations))) or ((scoping or systematic) adj2 (review or reviews or studies or study))).ti,ab,kf. | 486394 |
| 52 | ((review adj1 reviews) or ((systematic adj evidence) and (evidence adj map)) or (systematic adj2 mapping) or (systematic adj2 literature) or (systematic adj2 (Embase or Medline or PsycInfo or PubMed)) or (systematic adj2 (review or reviews)) or ((systematical or systematically) adj2 (review or reviewed reviews)) or (systematically adj identified) or (systematized adj review) or (umbrella adj (review or reviews))).ti,ab,kf. | 457244 |
| 53 | (meta adj2 (analyse or analyser or analyses or analysis or analytic or analytical or analytics or analyze or analyzed or analyzes)).ti,ab,kf. | 402399 |
| 54 | (metaanalyse or Metaanalysen or metaanalyser or metaanalyses or metaanalysis* or metaanalytic or metaanalytical or metaanalytics or metaanalyze or metaanalyzed or metaanalyzes).ti,ab,kf. | 13859 |
| 55 | network meta-analysis/ | 9733 |
| 56 | (network adj1 (meta or metaanalyses or metaanalysis or metaregression)).ti,ab,kf. | 15981 |
| 57 | (systematic and ((meta adj regression) or metagression)).ti,ab,kf. | 12395 |
| 58 | (((integrated or integrative or narrative) adj (review or reviews)) or overview or ((state adj3 art) and (art adj (review or reviews)))).ti,ab,kf. not (systematic or scoping).ti. | 314888 |
| 59 | or/33,37-58 | 3762440 |
| 60 | 32 and 59 | 1079 |
| 61 | limit 60 to yr="2016 -Current" | 824 |
|  |  |  |

PsychInfo ALL <1806 to May 22, 2024>

| **#** | **Query** | **Results** |
| --- | --- | --- |
| S60 | S58 AND S32 | 193 |
|  |  |  |
|  |  |  |
| S59 | S58 AND S32 | 263 |
|  |  |  |
|  |  |  |
| S58 | S33 OR S34 OR S35 OR S39 OR S40 OR S41 OR S42 OR S43 OR S44 OR S45 OR S46 OR S47 OR S48 OR S49 OR S50 OR S51 OR S52 OR S53 OR S54 OR S55 OR S56 OR S57 | 352174 |
|  |  |  |
|  |  |  |
| S57 | TI (((integrated or integrative or narrative) n1 (review or reviews)) or overview or ((state n3 art) and (art n1 (review or reviews)))) or AB (((integrated or integrative or narrative) n1 (review or reviews)) or overview or ((state n3 art) and (art n1 (review or reviews)))) | 77452 |
|  |  |  |
|  |  |  |
| S56 | TI (systematic and ((meta n1 regression) or metagression)) or AB (systematic and ((meta n1 regression) or metagression)) | 2702 |
|  |  |  |
|  |  |  |
| S55 | TI (network n1 (meta or metaanalyses or metaanalysis or metaregression)) or AB (network n1 (meta or metaanalyses or metaanalysis or metaregression)) | 4729 |
|  |  |  |
|  |  |  |
| S54 | TI (metaanalyse or Metaanalysen or metaanalyser or metaanalyses or metaanalysis* or metaanalytic or metaanalytical or metaanalytics or metaanalyze or metaanalyzed or metaanalyzes) or AB (metaanalyse or Metaanalysen or metaanalyser or metaanalyses or metaanalysis* or metaanalytic or metaanalytical or metaanalytics or metaanalyze or metaanalyzed or metaanalyzes) | 1417 |
|  |  |  |
|  |  |  |
| S53 | TI (meta n2 (analyse or analyser or analyses or analysis or analytic or analytical or analytics or analyze or analyzed or analyzes)) or AB (meta n2 (analyse or analyser or analyses or analysis or analytic or analytical or analytics or analyze or analyzed or analyzes)) | 119824 |
|  |  |  |
|  |  |  |
| S52 | TI ((review n1 reviews) or ((systematic n1 evidence) and (evidence n1 map)) or (systematic n2 mapping) or (systematic n2 literature) or (systematic n2 (Embase or Medline or PsycInfo or PubMed)) or (systematic n2 (review or reviews)) or ((systematical or systematically) n2 (review or reviewed reviews)) or (systematically n1 identified) or (systematized n1 review) or (umbrella n1 (review or reviews))) or AB ((review n1 reviews) or ((systematic n1 evidence) and (evidence n1 map)) or (systematic n2 mapping) or (systematic n2 literature) or (systematic n2 (Embase or Medline or PsycInfo or PubMed)) or (systematic n2 (review or reviews)) or ((systematical or systematically) n2 (review or reviewed reviews)) or (systematically n1 identified) or (systematized n1 review) or (umbrella n1 (review or reviews))) | 171,176 |
|  |  |  |
|  |  |  |
| S51 | TI (((review n1 economic) and (economic n1 (evaluation or evaluations))) or ((scoping or systematic) n2 (review or reviews or studies or study))) or AB (((review n1 economic) and (economic n1 (evaluation or evaluations))) or ((scoping or systematic) n2 (review or reviews or studies or study))) | 182986 |
|  |  |  |
|  |  |  |
| S50 | TI (((rapid n1 evidence) and (evidence n1 assessment)) or (rapid n1 realist) or (rapid n2 (review or reviews)) or (realist n2 (review or reviews or syntheses or synthesis))) or AB (((rapid n1 evidence) and (evidence n1 assessment)) or (rapid n1 realist) or (rapid n2 (review or reviews)) or (realist n2 (review or reviews or syntheses or synthesis))) | 2,441 |
|  |  |  |
|  |  |  |
| S49 | TI (((prognostic or psychometric) n1 review) or ((qualitative n1 (evidence or research)) and ((evidence or research) n1 synthesis))) or AB (((prognostic or psychometric) n1 review) or ((qualitative n1 (evidence or research)) and ((evidence or research) n1 synthesis))) | 806 |
|  |  |  |
|  |  |  |
| S48 | TI ((narrative n1 synthesis) or (overview n4 reviews) or ("PRISMA" n4 (guideline or guidelines or preferred or reporting or requirements)) or (PRISMA n "P")) or AB ((narrative n1 synthesis) or (overview n4 reviews) or ("PRISMA" n4 (guideline or guidelines or preferred or reporting or requirements)) or (PRISMA n "P")) | 18295 |
|  |  |  |
|  |  |  |
| S47 | TI ((mixed n1 methods) and (methods n1 (review or synthesis))) or AB ((mixed n1 methods) and (methods n1 (review or synthesis))) | 1085 |
|  |  |  |
|  |  |  |
| S46 | TI ((methodological or methodology) n1 review) or AB ((methodological or methodology) n1 review) | 2620 |
|  |  |  |
|  |  |  |
| S45 | TI ((meta n1 (epidemiological or ethnographic or ethnography or interpretation or narrative or review or study or synthesis or summary or theory)) or metaethnographic or metaethnography or metasynthesis) or AB ((meta n1 (epidemiological or ethnographic or ethnography or interpretation or narrative or review or study or synthesis or summary or theory)) or metaethnographic or metaethnography or metasynthesis) | 68783 |
|  |  |  |
|  |  |  |
| S44 | TI ((evidence n1 mapping) or (evidence n1 review) or (exploratory n1 review) or (framework n1 synthesis) or (mapping n1 review)) or AB ((evidence n1 mapping) or (evidence n1 review) or (exploratory n1 review) or (framework n1 synthesis) or (mapping n1 review)) | 12838 |
|  |  |  |
|  |  |  |
| S43 | TI ((evidence n1 assessment) and GRADE) or AB ((evidence n1 assessment) and GRADE) | 74 |
|  |  |  |
|  |  |  |
| S42 | TI ((diagnostic n1 test) and ((accuracy n1 review) or (accuracy n1 reviews) or (accuracy n1 studies) or (accuracy n1 study)) and (meta-analysis or scoping or systematic)) OR AB ((diagnostic n1 test) and ((accuracy n1 review) or (accuracy n1 reviews) or (accuracy n1 studies) or (accuracy n1 study)) and (meta-analysis or scoping or systematic)) | 253 |
|  |  |  |
|  |  |  |
| S41 | TI ((critical n1 interpretive) and ((interpretive n1 review) or (interpretive n1 synthesis))) or AB ((critical n1 interpretive) and ((interpretive n1 review) or (interpretive n1 synthesis))) | 162 |
|  |  |  |
|  |  |  |
| S40 | TI ((comparative n1 effectiveness) and (effectiveness n1 review)) or AB ((comparative n1 effectiveness) and (effectiveness n1 review)) | 68 |
|  |  |  |
|  |  |  |
| S39 | S36 AND S37 AND S38 | 31 |
|  |  |  |
|  |  |  |
| S38 | TI (eligibility or exclude d or exclusion or included or inclusion) or AB (eligibility or exclude d or exclusion or included or inclusion) | 714451 |
|  |  |  |
|  |  |  |
| S37 | (database or databases or cinahl or cochrane or embase or psycinfo or pubmed or medline or scopus or (web n1 science) or ((bibliographic or literature) n (review or reviews)) or ((electronic adj (database or databases)) or (databases n3 searched))) | 339999 |
|  |  |  |
|  |  |  |
| S36 | (((comprehensive or comprehensively) n (analysis or review or reviewed)) or ((literature or scoping) n (search or searches))) | 116 |
|  |  |  |
|  |  |  |
| S35 | ("Cochrane Database of Systematic Reviews" or evidence report technology assessment or evidence report technology assessment summary) | 3122 |
|  |  |  |
|  |  |  |
| S34 | MH “Meta analysis” | 75370 |
|  |  |  |
|  |  |  |
| S33 | MH "systematic review" | 137110 |
|  |  |  |
|  |  |  |
| S32 | S28 AND S31 | 2305 |
|  |  |  |
|  |  |  |
| S31 | S29 OR S30 | 2304158 |
|  |  |  |
|  |  |  |
| S30 | (design* OR develop* OR create* OR creation) | 1983903 |
|  |  |  |
|  |  |  |
| S29 | (participat* OR involve* OR co-design OR codesign OR user-centered OR “user centered” OR human-centered OR “human centered” OR user-participatory OR “user participatory”) | 585163 |
|  |  |  |
|  |  |  |
| S28 | S3 AND S27 | 5537 |
|  |  |  |
|  |  |  |
| S27 | S4 OR S5 OR S6 OR S7 OR S8 OR S9 OR S10 OR S11 OR S12 OR S13 OR S14 OR S15 OR S16 OR S17 OR S18 OR S19 OR S20 OR S21 OR S22 OR S23 OR S24 OR S25 OR S26 | 454513 |
|  |  |  |
|  |  |  |
| S26 | (artificial n2 intelligence) | 18485 |
|  |  |  |
|  |  |  |
| S25 | (telecare or "tele care" or telemedicine* or telerehabilitation* or telepsychiatry or telepsychology or teleconsultation* or remote consultation* or "tele therap*" or tele-therap* or teletherap* or videoconferenc* or "video conferenc*" or videoconsultation* or "video consultation*") | 38581 |
|  |  |  |
|  |  |  |
| S24 | ("head* up display*" or "head-up display*" or "head mount* display*" or "head-mount* display*" or "head* worn display*" or "head-worn* display*" or HUD* or "holographic display*" or "smart glass*") | 2318 |
|  |  |  |
|  |  |  |
| S23 | (Oculus or "Google Cardboard" or "Google Glass*" or "HTC Vive" or "Holo Lens" or "Samsung Gear" or "Magic Leap" or Wevr or NextVR or Pico or Varjo) | 2381 |
|  |  |  |
|  |  |  |
| S22 | ((mixed or extended) n2 realit*) | 353 |
|  |  |  |
|  |  |  |
| S21 | ((virtual or augmented) n2 (realit* or environment* or platform* or world*)) | 14598 |
|  |  |  |
|  |  |  |
| S20 | (wearable n2 (device* or technolog*)) | 2074 |
|  |  |  |
|  |  |  |
| S19 | ((mobile or software or electronic) n1 (app or apps or application)) | 17308 |
|  |  |  |
|  |  |  |
| S18 | (Tablet or iPad* or iPhone* or "handheld device*" or handheld-device* or "handheld computer*" or handheld-computer* or microcomputer* or palmtop* or laptop* or "personal digital assist*" or PDA or "telephone application*" or touch-screen* or touchscreen* or Robot*) | 45321 |
|  |  |  |
|  |  |  |
| S17 | ("mobile phone*" or Smartphone* or "smart phone*" or "Mobile device*") | 19678 |
|  |  |  |
|  |  |  |
| S16 | (E-health or ehealth* or "e health*" or mhealth or m-health or "m health" or "mobile health" or etherap* or e-therap* or (electronic adj therap*)) | 32134 |
|  |  |  |
|  |  |  |
| S15 | ("information communication technolog*" or ICT or Computer-assisted or computer-based or Web-based or "assistive technolog*") | 116794 |
|  |  |  |
|  |  |  |
| S14 | ((assistiv* or orthotic* or supportiv* or electronic* or welfare or everyday) n2 (technolog* or device*)) | 18122 |
|  |  |  |
|  |  |  |
| S13 | MH “Augmented Reality” | 738 |
|  |  |  |
|  |  |  |
| S12 | MH “Virtual Reality+” | 6810 |
|  |  |  |
|  |  |  |
| S11 | MH “Telemedicine+” or MH “telehealth+” | 39427 |
|  |  |  |
|  |  |  |
| S10 | MH “Wearable sensors+” | 8371 |
|  |  |  |
|  |  |  |
| S9 | MH “Microcomputers+” | 12,474 |
|  |  |  |
|  |  |  |
| S8 | MH “Computer Assisted therapy+” | 876 |
|  |  |  |
|  |  |  |
| S7 | MH “Mobile Applications” | 14140 |
|  |  |  |
|  |  |  |
| S6 | MH “Information technology+” | 24256 |
|  |  |  |
|  |  |  |
| S5 | MH “Internet+” | 167279 |
|  |  |  |
|  |  |  |
| S4 | MH “Assistive technology devices+” | 40396 |
|  |  |  |
|  |  |  |
| S3 | S1 OR S2 | 116143 |
|  |  |  |
|  |  |  |
| S2 | (dement* or alzheimer* or lewy or CJD or JCD or Creutzfeldt or binswanger or korsakoff or frontotemporal or FTD or VaD or "pick* disease") | 115939 |
|  |  |  |
|  |  |  |
| S1 | (MH "Dementia+") | 83447 |
|  |  |  |
|  |  |  |

CINAHL Plus <1806 to May 22, 2024>

| **#** | **Query** | **Results** |
| --- | --- | --- |
| S60 | S58 AND S32 | 143 |
|  |  |  |
|  |  |  |
| S59 | S58 AND S32 | 263 |
|  |  |  |
|  |  |  |
| S58 | S33 OR S34 OR S35 OR S39 OR S40 OR S41 OR S42 OR S43 OR S44 OR S45 OR S46 OR S47 OR S48 OR S49 OR S50 OR S51 OR S52 OR S53 OR S54 OR S55 OR S56 OR S57 | 352174 |
|  |  |  |
|  |  |  |
| S57 | TI (((integrated or integrative or narrative) n1 (review or reviews)) or overview or ((state n3 art) and (art n1 (review or reviews)))) or AB (((integrated or integrative or narrative) n1 (review or reviews)) or overview or ((state n3 art) and (art n1 (review or reviews)))) | 77452 |
|  |  |  |
|  |  |  |
| S56 | TI (systematic and ((meta n1 regression) or metagression)) or AB (systematic and ((meta n1 regression) or metagression)) | 2702 |
|  |  |  |
|  |  |  |
| S55 | TI (network n1 (meta or metaanalyses or metaanalysis or metaregression)) or AB (network n1 (meta or metaanalyses or metaanalysis or metaregression)) | 4729 |
|  |  |  |
|  |  |  |
| S54 | TI (metaanalyse or Metaanalysen or metaanalyser or metaanalyses or metaanalysis* or metaanalytic or metaanalytical or metaanalytics or metaanalyze or metaanalyzed or metaanalyzes) or AB (metaanalyse or Metaanalysen or metaanalyser or metaanalyses or metaanalysis* or metaanalytic or metaanalytical or metaanalytics or metaanalyze or metaanalyzed or metaanalyzes) | 1417 |
|  |  |  |
|  |  |  |
| S53 | TI (meta n2 (analyse or analyser or analyses or analysis or analytic or analytical or analytics or analyze or analyzed or analyzes)) or AB (meta n2 (analyse or analyser or analyses or analysis or analytic or analytical or analytics or analyze or analyzed or analyzes)) | 119824 |
|  |  |  |
|  |  |  |
| S52 | TI ((review n1 reviews) or ((systematic n1 evidence) and (evidence n1 map)) or (systematic n2 mapping) or (systematic n2 literature) or (systematic n2 (Embase or Medline or PsycInfo or PubMed)) or (systematic n2 (review or reviews)) or ((systematical or systematically) n2 (review or reviewed reviews)) or (systematically n1 identified) or (systematized n1 review) or (umbrella n1 (review or reviews))) or AB ((review n1 reviews) or ((systematic n1 evidence) and (evidence n1 map)) or (systematic n2 mapping) or (systematic n2 literature) or (systematic n2 (Embase or Medline or PsycInfo or PubMed)) or (systematic n2 (review or reviews)) or ((systematical or systematically) n2 (review or reviewed reviews)) or (systematically n1 identified) or (systematized n1 review) or (umbrella n1 (review or reviews))) | 171,176 |
|  |  |  |
|  |  |  |
| S51 | TI (((review n1 economic) and (economic n1 (evaluation or evaluations))) or ((scoping or systematic) n2 (review or reviews or studies or study))) or AB (((review n1 economic) and (economic n1 (evaluation or evaluations))) or ((scoping or systematic) n2 (review or reviews or studies or study))) | 182986 |
|  |  |  |
|  |  |  |
| S50 | TI (((rapid n1 evidence) and (evidence n1 assessment)) or (rapid n1 realist) or (rapid n2 (review or reviews)) or (realist n2 (review or reviews or syntheses or synthesis))) or AB (((rapid n1 evidence) and (evidence n1 assessment)) or (rapid n1 realist) or (rapid n2 (review or reviews)) or (realist n2 (review or reviews or syntheses or synthesis))) | 2,441 |
|  |  |  |
|  |  |  |
| S49 | TI (((prognostic or psychometric) n1 review) or ((qualitative n1 (evidence or research)) and ((evidence or research) n1 synthesis))) or AB (((prognostic or psychometric) n1 review) or ((qualitative n1 (evidence or research)) and ((evidence or research) n1 synthesis))) | 806 |
|  |  |  |
|  |  |  |
| S48 | TI ((narrative n1 synthesis) or (overview n4 reviews) or ("PRISMA" n4 (guideline or guidelines or preferred or reporting or requirements)) or (PRISMA n "P")) or AB ((narrative n1 synthesis) or (overview n4 reviews) or ("PRISMA" n4 (guideline or guidelines or preferred or reporting or requirements)) or (PRISMA n "P")) | 18295 |
|  |  |  |
|  |  |  |
| S47 | TI ((mixed n1 methods) and (methods n1 (review or synthesis))) or AB ((mixed n1 methods) and (methods n1 (review or synthesis))) | 1085 |
|  |  |  |
|  |  |  |
| S46 | TI ((methodological or methodology) n1 review) or AB ((methodological or methodology) n1 review) | 2620 |
|  |  |  |
|  |  |  |
| S45 | TI ((meta n1 (epidemiological or ethnographic or ethnography or interpretation or narrative or review or study or synthesis or summary or theory)) or metaethnographic or metaethnography or metasynthesis) or AB ((meta n1 (epidemiological or ethnographic or ethnography or interpretation or narrative or review or study or synthesis or summary or theory)) or metaethnographic or metaethnography or metasynthesis) | 68783 |
|  |  |  |
|  |  |  |
| S44 | TI ((evidence n1 mapping) or (evidence n1 review) or (exploratory n1 review) or (framework n1 synthesis) or (mapping n1 review)) or AB ((evidence n1 mapping) or (evidence n1 review) or (exploratory n1 review) or (framework n1 synthesis) or (mapping n1 review)) | 12838 |
|  |  |  |
|  |  |  |
| S43 | TI ((evidence n1 assessment) and GRADE) or AB ((evidence n1 assessment) and GRADE) | 74 |
|  |  |  |
|  |  |  |
| S42 | TI ((diagnostic n1 test) and ((accuracy n1 review) or (accuracy n1 reviews) or (accuracy n1 studies) or (accuracy n1 study)) and (meta-analysis or scoping or systematic)) OR AB ((diagnostic n1 test) and ((accuracy n1 review) or (accuracy n1 reviews) or (accuracy n1 studies) or (accuracy n1 study)) and (meta-analysis or scoping or systematic)) | 253 |
|  |  |  |
|  |  |  |
| S41 | TI ((critical n1 interpretive) and ((interpretive n1 review) or (interpretive n1 synthesis))) or AB ((critical n1 interpretive) and ((interpretive n1 review) or (interpretive n1 synthesis))) | 162 |
|  |  |  |
|  |  |  |
| S40 | TI ((comparative n1 effectiveness) and (effectiveness n1 review)) or AB ((comparative n1 effectiveness) and (effectiveness n1 review)) | 68 |
|  |  |  |
|  |  |  |
| S39 | S36 AND S37 AND S38 | 31 |
|  |  |  |
|  |  |  |
| S38 | TI (eligibility or exclude d or exclusion or included or inclusion) or AB (eligibility or exclude d or exclusion or included or inclusion) | 714451 |
|  |  |  |
|  |  |  |
| S37 | (database or databases or cinahl or cochrane or embase or psycinfo or pubmed or medline or scopus or (web n1 science) or ((bibliographic or literature) n (review or reviews)) or ((electronic adj (database or databases)) or (databases n3 searched))) | 339999 |
|  |  |  |
|  |  |  |
| S36 | (((comprehensive or comprehensively) n (analysis or review or reviewed)) or ((literature or scoping) n (search or searches))) | 116 |
|  |  |  |
|  |  |  |
| S35 | ("Cochrane Database of Systematic Reviews" or evidence report technology assessment or evidence report technology assessment summary) | 3122 |
|  |  |  |
|  |  |  |
| S34 | MH “Meta analysis” | 75370 |
|  |  |  |
|  |  |  |
| S33 | MH "systematic review" | 137110 |
|  |  |  |
|  |  |  |
| S32 | S28 AND S31 | 2305 |
|  |  |  |
|  |  |  |
| S31 | S29 OR S30 | 2304158 |
|  |  |  |
|  |  |  |
| S30 | (design* OR develop* OR create* OR creation) | 1983903 |
|  |  |  |
|  |  |  |
| S29 | (participat* OR involve* OR co-design OR codesign OR user-centered OR “user centered” OR human-centered OR “human centered” OR user-participatory OR “user participatory”) | 585163 |
|  |  |  |
|  |  |  |
| S28 | S3 AND S27 | 5537 |
|  |  |  |
|  |  |  |
| S27 | S4 OR S5 OR S6 OR S7 OR S8 OR S9 OR S10 OR S11 OR S12 OR S13 OR S14 OR S15 OR S16 OR S17 OR S18 OR S19 OR S20 OR S21 OR S22 OR S23 OR S24 OR S25 OR S26 | 454513 |
|  |  |  |
|  |  |  |
| S26 | (artificial n2 intelligence) | 18485 |
|  |  |  |
|  |  |  |
| S25 | (telecare or "tele care" or telemedicine* or telerehabilitation* or telepsychiatry or telepsychology or teleconsultation* or remote consultation* or "tele therap*" or tele-therap* or teletherap* or videoconferenc* or "video conferenc*" or videoconsultation* or "video consultation*") | 38581 |
|  |  |  |
|  |  |  |
| S24 | ("head* up display*" or "head-up display*" or "head mount* display*" or "head-mount* display*" or "head* worn display*" or "head-worn* display*" or HUD* or "holographic display*" or "smart glass*") | 2318 |
|  |  |  |
|  |  |  |
| S23 | (Oculus or "Google Cardboard" or "Google Glass*" or "HTC Vive" or "Holo Lens" or "Samsung Gear" or "Magic Leap" or Wevr or NextVR or Pico or Varjo) | 2381 |
|  |  |  |
|  |  |  |
| S22 | ((mixed or extended) n2 realit*) | 353 |
|  |  |  |
|  |  |  |
| S21 | ((virtual or augmented) n2 (realit* or environment* or platform* or world*)) | 14598 |
|  |  |  |
|  |  |  |
| S20 | (wearable n2 (device* or technolog*)) | 2074 |
|  |  |  |
|  |  |  |
| S19 | ((mobile or software or electronic) n1 (app or apps or application)) | 17308 |
|  |  |  |
|  |  |  |
| S18 | (Tablet or iPad* or iPhone* or "handheld device*" or handheld-device* or "handheld computer*" or handheld-computer* or microcomputer* or palmtop* or laptop* or "personal digital assist*" or PDA or "telephone application*" or touch-screen* or touchscreen* or Robot*) | 45321 |
|  |  |  |
|  |  |  |
| S17 | ("mobile phone*" or Smartphone* or "smart phone*" or "Mobile device*") | 19678 |
|  |  |  |
|  |  |  |
| S16 | (E-health or ehealth* or "e health*" or mhealth or m-health or "m health" or "mobile health" or etherap* or e-therap* or (electronic adj therap*)) | 32134 |
|  |  |  |
|  |  |  |
| S15 | ("information communication technolog*" or ICT or Computer-assisted or computer-based or Web-based or "assistive technolog*") | 116794 |
|  |  |  |
|  |  |  |
| S14 | ((assistiv* or orthotic* or supportiv* or electronic* or welfare or everyday) n2 (technolog* or device*)) | 18122 |
|  |  |  |
|  |  |  |
| S13 | MH “Augmented Reality” | 738 |
|  |  |  |
|  |  |  |
| S12 | MH “Virtual Reality+” | 6810 |
|  |  |  |
|  |  |  |
| S11 | MH “Telemedicine+” or MH “telehealth+” | 39427 |
|  |  |  |
|  |  |  |
| S10 | MH “Wearable sensors+” | 8371 |
|  |  |  |
|  |  |  |
| S9 | MH “Microcomputers+” | 12,474 |
|  |  |  |
|  |  |  |
| S8 | MH “Computer Assisted therapy+” | 876 |
|  |  |  |
|  |  |  |
| S7 | MH “Mobile Applications” | 14140 |
|  |  |  |
|  |  |  |
| S6 | MH “Information technology+” | 24256 |
|  |  |  |
|  |  |  |
| S5 | MH “Internet+” | 167279 |
|  |  |  |
|  |  |  |
| S4 | MH “Assistive technology devices+” | 40396 |
|  |  |  |
|  |  |  |
| S3 | S1 OR S2 | 116143 |
|  |  |  |
|  |  |  |
| S2 | (dement* or alzheimer* or lewy or CJD or JCD or Creutzfeldt or binswanger or korsakoff or frontotemporal or FTD or VaD or "pick* disease") | 115939 |
|  |  |  |
|  |  |  |
| S1 | (MH "Dementia+") | 83447 |
|  |  |  |
|  |  |  |

1. Usability searches

| **Platform and database**: Ovid MEDLINE® ALL <1946 to May 23, 2024> | | |
| --- | --- | --- |
| 1 | exp Dementia/ | 215188 |
| 2 | (dement* or alzheimer* or lewy or CJD or JCD or Creutzfeldt or binswanger or korsakoff or frontotemporal or FTD or VaD or "pick*1 disease").tw,kf. | 324042 |
| 3 | 1 or 2 | 360161 |
| 4 | exp Self-Help Devices/ or exp Internet/ or Information Technology/ or Mobile Applications/ or exp Therapy, Computer-Assisted/ or exp Microcomputers/ or exp Wearable Electronic Devices/ or exp Telemedicine/ or exp Virtual Reality/ or Augmented Reality/ | 260678 |
| 5 | ((assistiv* or orthotic* or supportiv* or electronic* or welfare or everyday) adj2 (technolog* or device*)).tw,kf. | 28796 |
| 6 | ("information communication technolog*" or ICT or Computer-assisted or computer-based or Web-based or "assistive technolog*").tw,kf. | 105753 |
| 7 | (E-health or ehealth* or "e health*" or mhealth or m-health or "m health" or "mobile health" or etherap* or e-therap* or (electronic adj therap*)).tw,kf. | 27132 |
| 8 | ("mobile phone*" or Smartphone* or "smart phone*" or "Mobile device*").tw,kf. | 44967 |
| 9 | (Tablet or iPad* or iPhone* or "handheld device*" or handheld-device* or "handheld computer*" or handheld-computer* or microcomputer* or palmtop* or laptop* or "personal digital assist*" or PDA or "telephone application*" or touch-screen* or touchscreen* or Robot*).tw,kf. | 146027 |
| 10 | ((mobile or software or electronic) adj1 (app or apps or application)).tw,kf. | 11322 |
| 11 | (wearable adj2 (device* or technolog*)).tw,kf. | 12168 |
| 12 | ((virtual or augmented) adj2 (realit* or environment* or platform* or world*)).tw,kf. | 29436 |
| 13 | ((mixed or extended) adj2 realit*).tw,kf. | 1474 |
| 14 | (Oculus or "Google Cardboard" or "Google Glass*" or "HTC Vive" or "Holo Lens" or "Samsung Gear" or "Magic Leap" or Wevr or NextVR or Pico or Varjo).tw,kf. | 6477 |
| 15 | ("head* up display*" or "head-up display*" or "head mount* display*" or "head-mount* display*" or "head* worn display*" or "head-worn* display*" or HUD* or "holographic display*" or "smart glass*").tw,kf. | 7703 |
| 16 | (telecare or "tele care" or telemedicine* or telerehabilitation* or telepsychiatry or telepsychology or teleconsultation* or remote consultation* or "tele therap*" or tele-therap* or teletherap* or videoconferenc* or "video conferenc*" or videoconsultation* or "video consultation*").tw,kf. | 39029 |
| 17 | (artificial adj2 intelligence).tw,kf. | 51689 |
| 18 | or/4-17 | 630503 |
| 19 | 3 and 18 | 5688 |
| 20 | User-Centered Design/ | 242 |
| 21 | (usability or "user experience*" or UX or "user-cent* design" or "user cent* design" or "human computer interaction*" or HCI or "human factor*" or "user feedback" or "user satisfaction").tw,kf. | 44321 |
| 22 | (acceptability or acceptance or accessibility or "ability to access" or "ease of access" or "ease of use" or "ability to use" or "easy to use" or user-friendl* or trust).tw,kf. | 327511 |
| 23 | or/20-22 | 363047 |
| 24 | 19 and 23 | 860 |
| 25 | ("systematic review" or "Meta-Analysis").pt. | 348470 |
| 26 | "Systematic Reviews as Topic"/ or "meta-analysis as topic"/ | 32500 |
| 27 | ("Cochrane Database of Systematic Reviews" or evidence report technology assessment or evidence report technology assessment summary).jn. | 16865 |
| 28 | (((comprehensive or comprehensively) adj (analysis or review or reviewed)) or ((literature or scoping) adj (search or searches))).ti,ab,kf. not "narrative review".ti. | 134609 |
| 29 | (database or databases or cinahl or cochrane or embase or psycinfo or pubmed or medline or scopus or (web adj1 science) or ((bibliographic or literature) adj (review or reviews)) or ((electronic adj (database or databases)) or (databases adj3 searched))).ti,ab,kf. | 1030378 |
| 30 | (eligibility or excluded or exclusion or included or inclusion).ti,ab,kf. | 2600968 |
| 31 | 28 and 29 and 30 | 38069 |
| 32 | ((comparative adj effectiveness) and (effectiveness adj review)).ti,ab,kf. | 134 |
| 33 | ((critical adj interpretive) and ((interpretive adj review) or (interpretive adj synthesis))).ti,ab,kf. | 227 |
| 34 | ((diagnostic adj test) and ((accuracy adj review) or (accuracy adj reviews) or (accuracy adj studies) or (accuracy adj study)) and (meta-analysis or scoping or systematic)).ti,ab,kf. | 650 |
| 35 | ((evidence adj assessment) and GRADE).ti,ab,kf. | 102 |
| 36 | ((evidence adj mapping) or (evidence adj review) or (exploratory adj review) or (framework adj synthesis) or (mapping adj review)).ti,ab,kf. | 3892 |
| 37 | ((meta adj (epidemiological or ethnographic or ethnography or interpretation or narrative or review or study or synthesis or summary or theory)) or metaethnographic or metaethnography or metasynthesis).ti,ab,kf. | 4257 |
| 38 | ((methodological or methodology) adj1 review).ti,ab,kf. | 3251 |
| 39 | ((mixed adj methods) and (methods adj1 (review or synthesis))).ti,ab,kf. | 474 |
| 40 | ((narrative adj1 synthesis) or (overview adj4 reviews) or ("PRISMA" adj4 (guideline or guidelines or preferred or reporting or requirements)) or (PRISMA adj "P")).ti,ab,kf. | 33714 |
| 41 | (((prognostic or psychometric) adj1 review) or ((qualitative adj (evidence or research)) and ((evidence or research) adj synthesis))).ti,ab,kf. | 868 |
| 42 | (((rapid adj evidence) and (evidence adj assessment)) or (rapid adj realist) or (rapid adj2 (review or reviews)) or (realist adj2 (review or reviews or syntheses or synthesis))).ti,ab,kf. | 4498 |
| 43 | (((review adj economic) and (economic adj1 (evaluation or evaluations))) or ((scoping or systematic) adj2 (review or reviews or studies or study))).ti,ab,kf. | 388644 |
| 44 | ((review adj1 reviews) or ((systematic adj evidence) and (evidence adj map)) or (systematic adj2 mapping) or (systematic adj2 literature) or (systematic adj2 (Embase or Medline or PsycInfo or PubMed)) or (systematic adj2 (review or reviews)) or ((systematical or systematically) adj2 (review or reviewed reviews)) or (systematically adj identified) or (systematized adj review) or (umbrella adj (review or reviews))).ti,ab,kf. | 360983 |
| 45 | (meta adj2 (analyse or analyser or analyses or analysis or analytic or analytical or analytics or analyze or analyzed or analyzes)).ti,ab,kf. | 307038 |
| 46 | (metaanalyse or Metaanalysen or metaanalyser or metaanalyses or metaanalysis* or metaanalytic or metaanalytical or metaanalytics or metaanalyze or metaanalyzed or metaanalyzes).ti,ab,kf. | 2801 |
| 47 | "network meta-analysis"/ | 6164 |
| 48 | (network adj1 (meta or metaanalyses or metaanalysis or metaregression)).ti,ab,kf. | 10856 |
| 49 | (systematic and ((meta adj regression) or metagression)).ti,ab,kf. | 9927 |
| 50 | review.pt. | 3326312 |
| 51 | (((integrated or integrative or narrative) adj (review or reviews)) or overview or ((state adj3 art) and (art adj (review or reviews)))).ti,ab,kf. not (systematic or scoping).ti. | 268898 |
| 52 | or/25-27,31-51 | 3726787 |
| 53 | 24 and 52 | 161 |
| **Platform and database**: Ovid Embase <1974 to 2024 May 23> | | |
| 1 | exp Dementia/ | 465773 |
| 2 | (dement* or alzheimer* or lewy or CJD or JCD or Creutzfeldt or binswanger or korsakoff or frontotemporal or FTD or VaD or "pick*1 disease").tw,kf. | 451002 |
| 3 | 1 or 2 | 575368 |
| 4 | exp Self Help Device/ or exp Internet/ or Information Technology/ or exp Mobile Application/ or exp Computer Assisted Therapy/ or Microcomputer/ or exp Wearable Computer/ or exp Telemedicine/ or Virtual Reality/ or Augmented Reality/ or Assistive Technology/ or exp Mobile Phone/ | 343947 |
| 5 | ((assistiv* or orthotic* or supportiv* or electronic* or welfare or everyday) adj2 (technolog* or device*)).tw,kf. | 33009 |
| 6 | ("information communication technolog*" or ICT or Computer-assisted or computer-based or Web-based or "assistive technolog*").tw,kf. | 139847 |
| 7 | (E-health or ehealth* or "e health*" or mhealth or m-health or "m health" or "mobile health" or etherap* or e-therap* or (electronic adj therap*)).tw,kf. | 28011 |
| 8 | ("mobile phone*" or Smartphone* or "smart phone*" or "Mobile device*").tw,kf. | 54964 |
| 9 | (Tablet or iPad* or iPhone* or "handheld device*" or handheld-device* or "handheld computer*" or handheld-computer* or microcomputer* or palmtop* or laptop* or "personal digital assist*" or PDA or "telephone application*" or touch-screen* or touchscreen* or Robot*).tw,kf. | 218298 |
| 10 | ((mobile or software or electronic) adj1 (app or apps or application)).tw,kf. | 14403 |
| 11 | (wearable adj2 (device* or technolog*)).tw,kf. | 13100 |
| 12 | ((virtual or augmented) adj2 (realit* or environment* or platform* or world*)).tw,kf. | 36303 |
| 13 | ((mixed or extended) adj2 realit*).tw,kf. | 1561 |
| 14 | (Oculus or "Google Cardboard" or "Google Glass*" or "HTC Vive" or "Holo Lens" or "Samsung Gear" or "Magic Leap" or Wevr or NextVR or Pico or Varjo).tw,kf. | 8781 |
| 15 | ("head* up display*" or "head-up display*" or "head mount* display*" or "head-mount* display*" or "head* worn display*" or "head-worn* display*" or HUD* or "holographic display*" or "smart glass*").tw,kf. | 10701 |
| 16 | (telecare or "tele care" or telemedicine* or telerehabilitation* or telepsychiatry or telepsychology or teleconsultation* or remote consultation* or "tele therap*" or tele-therap* or teletherap* or videoconferenc* or "video conferenc*" or videoconsultation* or "video consultation*").tw,kf. | 50239 |
| 17 | (artificial adj2 intelligence).tw,kf. | 59947 |
| 18 | or/4-17 | 800493 |
| 19 | 3 and 18 | 9930 |
| 20 | User-Centered Design/ or Usability/ or Human Computer Interaction/ | 12365 |
| 21 | (usability or "user experience*" or UX or "user-cent* design" or "user cent* design" or "human computer interaction*" or HCI or "human factor*" or "user feedback" or "user satisfaction").tw,kf. | 54340 |
| 22 | (acceptability or acceptance or accessibility or "ability to access" or "ease of access" or "ease of use" or "ability to use" or "easy to use" or user-friendl* or trust).tw,kf. | 430254 |
| 23 | or/20-22 | 479448 |
| 24 | 19 and 23 | 1265 |
| 25 | Systematic Review/ or Meta Analysis/ | 602291 |
| 26 | ("Cochrane Database of Systematic Reviews" or evidence report technology assessment or evidence report technology assessment summary).jn. | 17915 |
| 27 | (((comprehensive or comprehensively) adj (analysis or review or reviewed)) or ((literature or scoping) adj (search or searches))).ti,ab,kf. not "narrative review".ti. | 162142 |
| 28 | (database or databases or cinahl or cochrane or embase or psycinfo or pubmed or medline or scopus or (web adj1 science) or ((bibliographic or literature) adj (review or reviews)) or ((electronic adj (database or databases)) or (databases adj3 searched))).ti,ab,kf. | 1426904 |
| 29 | (eligibility or excluded or exclusion or included or inclusion).ti,ab,kf. | 4096790 |
| 30 | 27 and 28 and 29 | 48142 |
| 31 | ((comparative adj effectiveness) and (effectiveness adj review)).ti,ab,kf. | 95 |
| 32 | ((critical adj interpretive) and ((interpretive adj review) or (interpretive adj synthesis))).ti,ab,kf. | 231 |
| 33 | ((diagnostic adj test) and ((accuracy adj review) or (accuracy adj reviews) or (accuracy adj studies) or (accuracy adj study)) and (meta-analysis or scoping or systematic)).ti,ab,kf. | 759 |
| 34 | ((evidence adj assessment) and GRADE).ti,ab,kf. | 122 |
| 35 | ((evidence adj mapping) or (evidence adj review) or (exploratory adj review) or (framework adj synthesis) or (mapping adj review)).ti,ab,kf. | 4413 |
| 36 | ((meta adj (epidemiological or ethnographic or ethnography or interpretation or narrative or review or study or synthesis or summary or theory)) or metaethnographic or metaethnography or metasynthesis).ti,ab,kf. | 4693 |
| 37 | ((methodological or methodology) adj1 review).ti,ab,kf. | 3728 |
| 38 | ((mixed adj methods) and (methods adj1 (review or synthesis))).ti,ab,kf. | 470 |
| 39 | ((narrative adj1 synthesis) or (overview adj4 reviews) or ("PRISMA" adj4 (guideline or guidelines or preferred or reporting or requirements)) or (PRISMA adj "P")).ti,ab,kf. | 39486 |
| 40 | (((prognostic or psychometric) adj1 review) or ((qualitative adj (evidence or research)) and ((evidence or research) adj synthesis))).ti,ab,kf. | 1036 |
| 41 | (((rapid adj evidence) and (evidence adj assessment)) or (rapid adj realist) or (rapid adj2 (review or reviews)) or (realist adj2 (review or reviews or syntheses or synthesis))).ti,ab,kf. | 5171 |
| 42 | (((review adj economic) and (economic adj1 (evaluation or evaluations))) or ((scoping or systematic) adj2 (review or reviews or studies or study))).ti,ab,kf. | 465387 |
| 43 | ((review adj1 reviews) or ((systematic adj evidence) and (evidence adj map)) or (systematic adj2 mapping) or (systematic adj2 literature) or (systematic adj2 (Embase or Medline or PsycInfo or PubMed)) or (systematic adj2 (review or reviews)) or ((systematical or systematically) adj2 (review or reviewed reviews)) or (systematically adj identified) or (systematized adj review) or (umbrella adj (review or reviews))).ti,ab,kf. | 437994 |
| 44 | (meta adj2 (analyse or analyser or analyses or analysis or analytic or analytical or analytics or analyze or analyzed or analyzes)).ti,ab,kf. | 386618 |
| 45 | (metaanalyse or Metaanalysen or metaanalyser or metaanalyses or metaanalysis* or metaanalytic or metaanalytical or metaanalytics or metaanalyze or metaanalyzed or metaanalyzes).ti,ab,kf. | 13676 |
| 46 | Network Meta-Analysis/ | 9012 |
| 47 | (network adj1 (meta or metaanalyses or metaanalysis or metaregression)).ti,ab,kf. | 15040 |
| 48 | (systematic and ((meta adj regression) or metagression)).ti,ab,kf. | 11844 |
| 49 | review.pt. | 3229179 |
| 50 | (((integrated or integrative or narrative) adj (review or reviews)) or overview or ((state adj3 art) and (art adj (review or reviews)))).ti,ab,kf. not (systematic or scoping).ti. | 305117 |
| 51 | or/25-26,30-50 | 3853995 |
| 52 | 24 and 51 | 207 |
| **Platform and database**: Ovid APA PsycInfo <1806 to May Week 4 2024> | | |
| 1 | exp Dementia/ | 96022 |
| 2 | (dement* or alzheimer* or lewy or CJD or JCD or Creutzfeldt or binswanger or korsakoff or frontotemporal or FTD or VaD or "pick*1 disease").tw,hw. | 132026 |
| 3 | 1 or 2 | 132057 |
| 4 | exp Internet/ or exp "Information and Communication Technology"/ or exp Mobile Applications/ or exp Computer Assisted Therapy/ or Microcomputers/ or exp Wearable Devices/ or exp Telemedicine/ or exp Virtual Reality/ | 217872 |
| 5 | ((assistiv* or orthotic* or supportiv* or electronic* or welfare or everyday) adj2 (technolog* or device*)).tw,hw. | 7209 |
| 6 | ("information communication technolog*" or ICT or Computer-assisted or computer-based or Web-based or "assistive technolog*").tw,hw. | 59568 |
| 7 | (E-health or ehealth* or "e health*" or mhealth or m-health or "m health" or "mobile health" or etherap* or e-therap* or (electronic adj therap*)).tw,hw. | 6763 |
| 8 | ("mobile phone*" or Smartphone* or "smart phone*" or "Mobile device*").tw,hw. | 19026 |
| 9 | (Tablet or iPad* or iPhone* or "handheld device*" or handheld-device* or "handheld computer*" or handheld-computer* or microcomputer* or palmtop* or laptop* or "personal digital assist*" or PDA or "telephone application*" or touch-screen* or touchscreen* or Robot*).tw,hw. | 24367 |
| 10 | ((mobile or software or electronic) adj1 (app or apps or application)).tw,hw. | 3165 |
| 11 | (wearable adj2 (device* or technolog*)).tw,hw. | 1687 |
| 12 | ((virtual or augmented) adj2 (realit* or environment* or platform* or world*)).tw,hw. | 20120 |
| 13 | ((mixed or extended) adj2 realit*).tw,hw. | 472 |
| 14 | (Oculus or "Google Cardboard" or "Google Glass*" or "HTC Vive" or "Holo Lens" or "Samsung Gear" or "Magic Leap" or Wevr or NextVR or Pico or Varjo).tw,hw. | 543 |
| 15 | ("head* up display*" or "head-up display*" or "head mount* display*" or "head-mount* display*" or "head* worn display*" or "head-worn* display*" or HUD* or "holographic display*" or "smart glass*").tw,hw. | 3244 |
| 16 | (telecare or "tele care" or telemedicine* or telerehabilitation* or telepsychiatry or telepsychology or teleconsultation* or remote consultation* or "tele therap*" or tele-therap* or teletherap* or videoconferenc* or "video conferenc*" or videoconsultation* or "video consultation*").tw,hw. | 13648 |
| 17 | (artificial adj2 intelligence).tw,hw. | 15366 |
| 18 | or/4-17 | 274011 |
| 19 | 3 and 18 | 3137 |
| 20 | Human Factors Engineering/ or exp Human Computer Interaction/ or Technology Acceptance/ | 37270 |
| 21 | (usability or "user experience*" or UX or "user-cent* design" or "user cent* design" or "human computer interaction*" or HCI or "human factor*" or "user feedback" or "user satisfaction").tw,hw. | 35226 |
| 22 | (acceptability or acceptance or accessibility or "ability to access" or "ease of access" or "ease of use" or "ability to use" or "easy to use" or user-friendl* or trust).tw,hw. | 161729 |
| 23 | or/20-22 | 204820 |
| 24 | 19 and 23 | 526 |
| 25 | Systematic Review/ or Meta Analysis/ | 6174 |
| 26 | ("Cochrane Database of Systematic Reviews" or evidence report technology assessment or evidence report technology assessment summary).jn. | 0 |
| 27 | (((comprehensive or comprehensively) adj (analysis or review or reviewed)) or ((literature or scoping) adj (search or searches))).ti,ab,hw. not "narrative review".ti. | 18499 |
| 28 | (database or databases or cinahl or cochrane or embase or psycinfo or pubmed or medline or scopus or (web adj1 science) or ((bibliographic or literature) adj (review or reviews)) or ((electronic adj (database or databases)) or (databases adj3 searched))).ti,ab,hw. | 5326266 |
| 29 | (eligibility or excluded or exclusion or included or inclusion).ti,ab,hw. | 477312 |
| 30 | 27 and 28 and 29 | 6772 |
| 31 | ((comparative adj effectiveness) and (effectiveness adj review)).ti,ab,hw. | 7 |
| 32 | ((critical adj interpretive) and ((interpretive adj review) or (interpretive adj synthesis))).ti,ab,hw. | 117 |
| 33 | ((diagnostic adj test) and ((accuracy adj review) or (accuracy adj reviews) or (accuracy adj studies) or (accuracy adj study)) and (meta-analysis or scoping or systematic)).ti,ab,hw. | 33 |
| 34 | ((evidence adj assessment) and GRADE).ti,ab,hw. | 8 |
| 35 | ((evidence adj mapping) or (evidence adj review) or (exploratory adj review) or (framework adj synthesis) or (mapping adj review)).ti,ab,hw. | 604 |
| 36 | ((meta adj (epidemiological or ethnographic or ethnography or interpretation or narrative or review or study or synthesis or summary or theory)) or metaethnographic or metaethnography or metasynthesis).ti,ab,hw. | 2696 |
| 37 | ((methodological or methodology) adj1 review).ti,ab,hw. | 1140 |
| 38 | ((mixed adj methods) and (methods adj1 (review or synthesis))).ti,ab,hw. | 174 |
| 39 | ((narrative adj1 synthesis) or (overview adj4 reviews) or ("PRISMA" adj4 (guideline or guidelines or preferred or reporting or requirements)) or (PRISMA adj "P")).ti,ab,hw. | 5870 |
| 40 | (((prognostic or psychometric) adj1 review) or ((qualitative adj (evidence or research)) and ((evidence or research) adj synthesis))).ti,ab,hw. | 301 |
| 41 | (((rapid adj evidence) and (evidence adj assessment)) or (rapid adj realist) or (rapid adj2 (review or reviews)) or (realist adj2 (review or reviews or syntheses or synthesis))).ti,ab,hw. | 973 |
| 42 | (((review adj economic) and (economic adj1 (evaluation or evaluations))) or ((scoping or systematic) adj2 (review or reviews or studies or study))).ti,ab,hw. | 66421 |
| 43 | ((review adj1 reviews) or ((systematic adj evidence) and (evidence adj map)) or (systematic adj2 mapping) or (systematic adj2 literature) or (systematic adj2 (Embase or Medline or PsycInfo or PubMed)) or (systematic adj2 (review or reviews)) or ((systematical or systematically) adj2 (review or reviewed reviews)) or (systematically adj identified) or (systematized adj review) or (umbrella adj (review or reviews))).ti,ab,hw. | 60322 |
| 44 | (meta adj2 (analyse or analyser or analyses or analysis or analytic or analytical or analytics or analyze or analyzed or analyzes)).ti,ab,hw. | 55452 |
| 45 | (metaanalyse or Metaanalysen or metaanalyser or metaanalyses or metaanalysis* or metaanalytic or metaanalytical or metaanalytics or metaanalyze or metaanalyzed or metaanalyzes).ti,ab,hw. | 831 |
| 46 | (network adj1 (meta or metaanalyses or metaanalysis or metaregression)).ti,ab,hw. | 800 |
| 47 | (systematic and ((meta adj regression) or metagression)).ti,ab,hw. | 1423 |
| 48 | (((integrated or integrative or narrative) adj (review or reviews)) or overview or ((state adj3 art) and (art adj (review or reviews)))).ti,ab,hw. not (systematic or scoping).ti. | 90873 |
| 49 | or/25-26,30-48 | 198992 |
| 50 | 24 and 49 | 60 |
| **Platform and database**: EBSCOhost Research Databases CINAHL Plus with Full Text | | |
| S1 | (MH "Dementia+") | 85,933 |
| S2 | TI ( dement* or alzheimer* or lewy or CJD or JCD or Creutzfeldt or binswanger or korsakoff or frontotemporal or FTD or VaD or "pick*1 disease" ) OR AB ( dement* or alzheimer* or lewy or CJD or JCD or Creutzfeldt or binswanger or korsakoff or frontotemporal or FTD or VaD or "pick*1 disease" ) | 98,402 |
| S3 | S1 OR S2 | 117,314 |
| S4 | (MH "Assistive Technology Devices+") OR (MH "Internet+") OR (MH "Information Technology+") OR (MH "Mobile Applications") OR (MH "Therapy, Computer Assisted+") OR (MH "Microcomputers+") OR (MH "Wearable Sensors+") OR (MH "Telemedicine+") OR (MH "Augmented Reality") OR (MH "Virtual Reality+") | 294,022 |
| S5 | TI ( (assistiv* or orthotic* or supportiv* or electronic* or welfare or everyday) N2 (technolog* or device*) ) OR AB ( (assistiv* or orthotic* or supportiv* or electronic* or welfare or everyday) N2 (technolog* or device*) ) | 10,005 |
| S6 | TI ( "information communication technolog*" or ICT or Computer-assisted or computer-based or Web-based or "assistive technolog*" ) OR AB ( "information communication technolog*" or ICT or Computer-assisted or computer-based or Web-based or "assistive technolog*" ) | 36,325 |
| S7 | TI ( E-health or ehealth* or "e health*" or mhealth or m-health or "m health" or "mobile health" or etherap* or e-therap* or (electronic N1 therap*) ) OR AB ( E-health or ehealth* or "e health*" or mhealth or m-health or "m health" or "mobile health" or etherap* or e-therap* or (electronic N1 therap*) ) | 9,361 |
| S8 | TI ( "mobile phone*" or Smartphone* or "smart phone*" or "Mobile device*" ) OR AB ( "mobile phone*" or Smartphone* or "smart phone*" or "Mobile device*" ) | 14,765 |
| S9 | TI ( Tablet or iPad* or iPhone* or "handheld device*" or handheld-device* or "handheld computer*" or handheld-computer* or microcomputer* or palmtop* or laptop* or "personal digital assist*" or PDA or "telephone application*" or touch-screen* or touchscreen* or Robot* ) OR AB ( Tablet or iPad* or iPhone* or "handheld device*" or handheld-device* or "handheld computer*" or handheld-computer* or microcomputer* or palmtop* or laptop* or "personal digital assist*" or PDA or "telephone application*" or touch-screen* or touchscreen* or Robot* ) | 35,244 |
| S10 | TI ( (mobile or software or electronic) N1 (app or apps or application) ) OR AB ( (mobile or software or electronic) N1 (app or apps or application) ) | 6,562 |
| S11 | TI ( wearable N2 (device* or technolog*) ) OR AB ( wearable N2 (device* or technolog*) ) | 2,006 |
| S12 | TI ( (virtual or augmented) N2 (realit* or environment* or platform* or world*) ) OR AB ( (virtual or augmented) N2 (realit* or environment* or platform* or world*) ) | 11,300 |
| S13 | TI ( (mixed or extended) N2 realit* ) OR AB ( (mixed or extended) N2 realit* ) | 325 |
| S14 | TI ( Oculus or "Google Cardboard" or "Google Glass*" or "HTC Vive" or "Holo Lens" or "Samsung Gear" or "Magic Leap" or Wevr or NextVR or Pico or Varjo ) OR AB ( Oculus or "Google Cardboard" or "Google Glass*" or "HTC Vive" or "Holo Lens" or "Samsung Gear" or "Magic Leap" or Wevr or NextVR or Pico or Varjo ) | 2,264 |
| S15 | TI ( "head* up display*" or "head-up display*" or "head mount* display*" or "head-mount* display*" or "head* worn display*" or "head-worn* display*" or HUD* or "holographic display*" or "smart glass*" ) OR AB ( "head* up display*" or "head-up display*" or "head mount* display*" or "head-mount* display*" or "head* worn display*" or "head-worn* display*" or HUD* or "holographic display*" or "smart glass*" ) | 2,262 |
| S16 | TI ( telecare or "tele care" or telemedicine* or telerehabilitation* or telepsychiatry or telepsychology or teleconsultation* or remote consultation* or "tele therap*" or tele-therap* or teletherap* or videoconferenc* or "video conferenc*" or videoconsultation* or "video consultation*" ) OR AB ( telecare or "tele care" or telemedicine* or telerehabilitation* or telepsychiatry or telepsychology or teleconsultation* or remote consultation* or "tele therap*" or tele-therap* or teletherap* or videoconferenc* or "video conferenc*" or videoconsultation* or "video consultation*" ) | 13,803 |
| S17 | TI artificial N2 intelligence OR AB artificial N2 intelligence | 9,618 |
| S18 | S4 OR S5 OR S6 OR S7 OR S8 OR S9 OR S10 OR S11 OR S12 OR S13 OR S14 OR S15 OR S16 OR S17 | 382,857 |
| S19 | S3 AND S18 | 4,076 |
| S20 | (MH "User-Computer Interface+") OR (MH "Consumer Satisfaction+") | 93,163 |
| S21 | TI ( usability or "user experience*" or UX or "user-cent* design" or "user cent* design" or "human computer interaction*" or HCI or "human factor*" or "user feedback" or "user satisfaction" ) OR AB ( usability or "user experience*" or UX or "user-cent* design" or "user cent* design" or "human computer interaction*" or HCI or "human factor*" or "user feedback" or "user satisfaction" ) | 15,057 |
| S22 | TI ( acceptability or acceptance or accessibility or "ability to access" or "ease of access" or "ease of use" or "ability to use" or "easy to use" or user-friendl* or trust ) OR AB ( acceptability or acceptance or accessibility or "ability to access" or "ease of access" or "ease of use" or "ability to use" or "easy to use" or user-friendl* or trust ) | 114,494 |
| S23 | S20 OR S21 OR S22 | 210,722 |
| S24 | S19 AND S23 | 499 |
| S25 | (MH "Meta Analysis") OR (MH "Systematic Review") | 165,011 |
| S26 | PT (Meta Analysis OR Review OR Systematic Review) | 539,251 |
| S27 | SO ("Cochrane Database of Systematic Reviews" or "evidence report technology assessment" or "evidence report technology assessment summary") | 6,023 |
| S28 | ( TI (((comprehensive or comprehensively) N1 (analysis or review or reviewed)) or ((literature or scoping) N1 (search or searches))) OR AB (((comprehensive or comprehensively) N1 (analysis or review or reviewed)) or ((literature or scoping) N1 (search or searches))) ) NOT TI "narrative review" | 41,115 |
| S29 | TI ( database or databases or cinahl or cochrane or embase or psycinfo or pubmed or medline or scopus or (web N1 science) or ((bibliographic or literature) N1 (review or reviews)) or ((electronic N1 (database or databases)) or (databases N3 searched)) ) OR AB ( database or databases or cinahl or cochrane or embase or psycinfo or pubmed or medline or scopus or (web N1 science) or ((bibliographic or literature) N1 (review or reviews)) or ((electronic N1 (database or databases)) or (databases N3 searched)) ) | 314,248 |
| S30 | TI ( eligibility or excluded or exclusion or included or inclusion ) OR AB ( eligibility or excluded or exclusion or included or inclusion ) | 729,074 |
| S31 | S28 AND S29 AND S30 | 15,189 |
| S32 | TI ( (comparative N1 effectiveness) and (effectiveness N1 review) ) OR AB ( (comparative N1 effectiveness) and (effectiveness N1 review) ) | 68 |
| S33 | TI ( (critical N1 interpretive) and ((interpretive N1 review) or (interpretive N1 synthesis)) ) OR AB ( (critical N1 interpretive) and ((interpretive N1 review) or (interpretive N1 synthesis)) ) | 158 |
| S34 | TI ( (diagnostic N1 test) and ((accuracy N1 review) or (accuracy N1 reviews) or (accuracy N1 studies) or (accuracy N1 study)) and (meta-analysis or scoping or systematic)) ) OR AB ( (diagnostic N1 test) and ((accuracy N1 review) or (accuracy N1 reviews) or (accuracy N1 studies) or (accuracy N1 study)) and (meta-analysis or scoping or systematic)) ) | 247 |
| S35 | TI ( (evidence N1 assessment) and GRADE) ) OR AB ( (evidence N1 assessment) and GRADE) ) | 72 |
| S36 | TI ( (evidence N1 mapping) or (evidence N1 review) or (exploratory N1 review) or (framework N1 synthesis) or (mapping N1 review) ) OR AB ( (evidence N1 mapping) or (evidence N1 review) or (exploratory N1 review) or (framework N1 synthesis) or (mapping N1 review) ) | 12,669 |
| S37 | TI ( (meta N1 (epidemiological or ethnographic or ethnography or interpretation or narrative or review or study or synthesis or summary or theory)) or metaethnographic or metaethnography or metasynthesis) ) OR AB ( (meta N1 (epidemiological or ethnographic or ethnography or interpretation or narrative or review or study or synthesis or summary or theory)) or metaethnographic or metaethnography or metasynthesis) ) | 66,089 |
| S38 | TI ( (methodological or methodology) N1 review) ) OR AB ( (methodological or methodology) N1 review) ) | 2,556 |
| S39 | TI ( (mixed N1 methods) and (methods N1 (review or synthesis)) ) OR AB ( (mixed N1 methods) and (methods N1 (review or synthesis)) ) | 1,027 |
| S40 | TI ( (narrative N1 synthesis) or (overview N4 reviews) or ("PRISMA" N4 (guideline or guidelines or preferred or reporting or requirements)) or (PRISMA N1 "P")) ) OR AB ( (narrative N1 synthesis) or (overview N4 reviews) or ("PRISMA" N4 (guideline or guidelines or preferred or reporting or requirements)) or (PRISMA N1 "P")) ) | 17,819 |
| S41 | TI ( ((prognostic or psychometric) N1 review) or ((qualitative N1 (evidence or research)) and ((evidence or research) N1 synthesis)) ) OR AB ( ((prognostic or psychometric) N1 review) or ((qualitative N1 (evidence or research)) and ((evidence or research) N1 synthesis)) ) | 781 |
| S42 | TI ( ((rapid N1 evidence) and (evidence N1 assessment)) or (rapid N1 realist) or (rapid N2 (review or reviews)) or (realist N2 (review or reviews or syntheses or synthesis)) ) OR AB ( ((rapid N1 evidence) and (evidence N1 assessment)) or (rapid N1 realist) or (rapid N2 (review or reviews)) or (realist N2 (review or reviews or syntheses or synthesis)) ) | 2,361 |
| S43 | TI ( ((review N1 economic) and (economic N1 (evaluation or evaluations))) or ((scoping or systematic) N2 (review or reviews or studies or study)) ) OR AB ( ((review N1 economic) and (economic N1 (evaluation or evaluations))) or ((scoping or systematic) N2 (review or reviews or studies or study)) ) | 177,006 |
| S44 | TI ( (review N1 reviews) or ((systematic N1 evidence) and (evidence N1 map)) or (systematic N2 mapping) or (systematic N2 literature) or (systematic N2 (Embase or Medline or PsycInfo or PubMed)) or (systematic N2 (review or reviews)) or ((systematical or systematically) N2 (review or reviewed reviews)) or (systematically N1 identified) or (systematized N1 review) or (umbrella N1 (review or reviews)) ) OR AB ( (review N1 reviews) or ((systematic N1 evidence) and (evidence N1 map)) or (systematic N2 mapping) or (systematic N2 literature) or (systematic N2 (Embase or Medline or PsycInfo or PubMed)) or (systematic N2 (review or reviews)) or ((systematical or systematically) N2 (review or reviewed reviews)) or (systematically N1 identified) or (systematized N1 review) or (umbrella N1 (review or reviews)) ) | 170,991 |
| S45 | TI ( (meta N2 (analyse or analyser or analyses or analysis or analytic or analytical or analytics or analyze or analyzed or analyzes) ) OR AB ( (meta N2 (analyse or analyser or analyses or analysis or analytic or analytical or analytics or analyze or analyzed or analyzes) ) | 116,223 |
| S46 | TI ( (metaanalyse or Metaanalysen or metaanalyser or metaanalyses or metaanalysis* or metaanalytic or metaanalytical or metaanalytics or metaanalyze or metaanalyzed or metaanalyzes) ) OR AB ( (metaanalyse or Metaanalysen or metaanalyser or metaanalyses or metaanalysis* or metaanalytic or metaanalytical or metaanalytics or metaanalyze or metaanalyzed or metaanalyzes) ) | 1,395 |
| S47 | TI ( network N1 (meta or metaanalyses or metaanalysis or metaregression) ) OR AB ( network N1 (meta or metaanalyses or metaanalysis or metaregression) ) | 4,527 |
| S48 | TI ( systematic and ((meta N1 regression) or metagression) ) OR AB ( systematic and ((meta N1 regression) or metagression) ) | 2,619 |
| S49 | ( TI ((integrated or integrative or narrative) N1 (review or reviews)) or overview or ((state N3 art) and (art N1 (review or reviews))) OR AB ((integrated or integrative or narrative) N1 (review or reviews)) or overview or ((state N3 art) and (art N1 (review or reviews)))) not TI (systematic or scoping) | 73,281 |
| S50 | S25 OR S26 OR S27 OR S31 OR S32 OR S33 OR S34 OR S35 OR S36 OR S37 OR S38 OR S39 OR S40 OR S41 OR S42 OR S43 OR S44 OR S45 OR S46 OR S47 OR S48 OR S49 | 679,018 |
| S51 | S24 AND S50 | 60 |

1. Implementation searches

*Please note the missing data for searches in MEDLINE, Embase and Psych Info. The line by line search results were lost due to an administrative error.

Ovid MEDLINE ® ALL <1946 to August 5, 2024>

| **#** | **Search term(s)** | **Result(s)** |
| --- | --- | --- |
| 1 | exp Dementia/ | * |
| 2 | (dement* or alzheimer* or lewy or CJD or JCD or Creutzfeldt or binswanger or korsakoff or frontotemporal or FTD or VaD or "pick*1 disease").tw,kf. | * |
| 3 | 1 or 2 | * |
| 4 | exp Self-Help Devices/ or exp Internet/ or Information Technology/ or Mobile Applications/ or exp Therapy, Computer-Assisted/ or exp Microcomputers/ or exp Wearable Electronic Devices/ or exp Telemedicine/ or exp Virtual Reality/ or Augmented Reality/ | * |
| 5 | ((assistiv* or orthotic* or supportiv* or electronic* or welfare or everyday) adj2 (technolog* or device*)).tw,kf. | * |
| 6 | ("information communication technolog*" or ICT or Computer-assisted or computer-based or Web-based or "assistive technolog*").tw,kf. | * |
| 7 | (E-health or ehealth* or "e health*" or mhealth or m-health or "m health" or "mobile health" or etherap* or e-therap* or (electronic adj therap*)).tw,kf. | * |
| 8 | ("mobile phone*" or Smartphone* or "smart phone*" or "Mobile device*").tw,kf. | * |
| 9 | (Tablet or iPad* or iPhone* or "handheld device*" or handheld-device* or "handheld computer*" or handheld-computer* or microcomputer* or palmtop* or laptop* or "personal digital assist*" or PDA or "telephone application*" or touch-screen* or touchscreen* or Robot*).tw,kf. | * |
| 10 | ((mobile or software or electronic) adj1 (app or apps or application)).tw,kf. | * |
| 11 | (wearable adj2 (device* or technolog*)).tw,kf. | * |
| 12 | ((virtual or augmented) adj2 (realit* or environment* or platform* or world*)).tw,kf. | * |
| 13 | ((mixed or extended) adj2 realit*).tw,kf. | * |
| 14 | (Oculus or "Google Cardboard" or "Google Glass*" or "HTC Vive" or "Holo Lens" or "Samsung Gear" or "Magic Leap" or Wevr or NextVR or Pico or Varjo).tw,kf. | * |
| 15 | ("head* up display*" or "head-up display*" or "head mount* display*" or "head-mount* display*" or "head* worn display*" or "head-worn* display*" or HUD* or "holographic display*" or "smart glass*").tw,kf. | * |
| 16 | (telecare or "tele care" or telemedicine* or telerehabilitation* or telepsychiatry or telepsychology or teleconsultation* or remote consultation* or "tele therap*" or tele-therap* or teletherap* or videoconferenc* or "video conferenc*" or videoconsultation* or "video consultation*").tw,kf. | * |
| 17 | (artificial adj2 intelligence).tw,kf. | * |
| 18 | or/4-17 | * |
| 19 | 3 and 18 | * |
| 20 | "Treatment Adherence and Compliance"/ | * |
| 21 | (((technology or intervention) adj2 ("use" or "using")) or (adher* or adopt* or implement* or barrier or facilitator or enabl* or optimise)).kf,tw. | * |
| 22 | 20 or 21 | * |
| 23 | ("systematic review" or "Meta-Analysis").pt. | * |
| 24 | "Systematic Reviews as Topic"/ or "meta-analysis as topic"/ | * |
| 25 | ("Cochrane Database of Systematic Reviews" or evidence report technology assessment or evidence report technology assessment summary).jn. | * |
| 26 | (((comprehensive or comprehensively) adj (analysis or review or reviewed)) or ((literature or scoping) adj (search or searches))).ti,ab,kf. not "narrative review".ti. | * |
| 27 | (database or databases or cinahl or cochrane or embase or psycinfo or pubmed or medline or scopus or (web adj1 science) or ((bibliographic or literature) adj (review or reviews)) or ((electronic adj (database or databases)) or (databases adj3 searched))).ti,ab,kf. | * |
| 28 | (eligibility or excluded or exclusion or included or inclusion).ti,ab,kf. | * |
| 29 | 26 and 27 and 28 | * |
| 30 | ((comparative adj effectiveness) and (effectiveness adj review)).ti,ab,kf. | * |
| 31 | ((critical adj interpretive) and ((interpretive adj review) or (interpretive adj synthesis))).ti,ab,kf. | * |
| 32 | ((diagnostic adj test) and ((accuracy adj review) or (accuracy adj reviews) or (accuracy adj studies) or (accuracy adj study)) and (meta-analysis or scoping or systematic)).ti,ab,kf. | * |
| 33 | ((evidence adj assessment) and GRADE).ti,ab,kf. | * |
| 34 | ((evidence adj mapping) or (evidence adj review) or (exploratory adj review) or (framework adj synthesis) or (mapping adj review)).ti,ab,kf. | * |
| 35 | ((meta adj (epidemiological or ethnographic or ethnography or interpretation or narrative or review or study or synthesis or summary or theory)) or metaethnographic or metaethnography or metasynthesis).ti,ab,kf. | * |
| 36 | ((methodological or methodology) adj1 review).ti,ab,kf. | * |
| 37 | ((mixed adj methods) and (methods adj1 (review or synthesis))).ti,ab,kf. | * |
| 38 | ((narrative adj1 synthesis) or (overview adj4 reviews) or ("PRISMA" adj4 (guideline or guidelines or preferred or reporting or requirements)) or (PRISMA adj "P")).ti,ab,kf. | * |
| 39 | (((prognostic or psychometric) adj1 review) or ((qualitative adj (evidence or research)) and ((evidence or research) adj synthesis))).ti,ab,kf. | * |
| 40 | (((rapid adj evidence) and (evidence adj assessment)) or (rapid adj realist) or (rapid adj2 (review or reviews)) or (realist adj2 (review or reviews or syntheses or synthesis))).ti,ab,kf. | * |
| 41 | (((review adj economic) and (economic adj1 (evaluation or evaluations))) or ((scoping or systematic) adj2 (review or reviews or studies or study))).ti,ab,kf. | * |
| 42 | ((review adj1 reviews) or ((systematic adj evidence) and (evidence adj map)) or (systematic adj2 mapping) or (systematic adj2 literature) or (systematic adj2 (Embase or Medline or PsycInfo or PubMed)) or (systematic adj2 (review or reviews)) or ((systematical or systematically) adj2 (review or reviewed reviews)) or (systematically adj identified) or (systematized adj review) or (umbrella adj (review or reviews))).ti,ab,kf. | * |
| 43 | (meta adj2 (analyse or analyser or analyses or analysis or analytic or analytical or analytics or analyze or analyzed or analyzes)).ti,ab,kf. | * |
| 44 | (metaanalyse or Metaanalysen or metaanalyser or metaanalyses or metaanalysis* or metaanalytic or metaanalytical or metaanalytics or metaanalyze or metaanalyzed or metaanalyzes).ti,ab,kf. | * |
| 45 | "network meta-analysis"/ | * |
| 46 | (network adj1 (meta or metaanalyses or metaanalysis or metaregression)).ti,ab,kf. | * |
| 47 | (systematic and ((meta adj regression) or metagression)).ti,ab,kf. | * |
| 48 | review.pt. | * |
| 49 | (((integrated or integrative or narrative) adj (review or reviews)) or overview or ((state adj3 art) and (art adj (review or reviews)))).ti,ab,kf. not (systematic or scoping).ti. | * |
| 50 | 23 or 24 or 25 or 29 or 30 or 31 or 32 or 33 or 34 or 35 or 36 or 37 or 38 or 39 or 40 or 41 or 42 or 43 or 44 or 45 or 46 or 47 or 48 or 49 | * |
| 51 | 19 and 22 and 50 | 369 |

Ovid Embase(R) All + PsychInfo All <1947 to August 5, 2024>

| **#** | **Search term(s)** | **Result(s)** |
| --- | --- | --- |
| 1 | exp Dementia/ | * |
| 2 | (dement* or alzheimer* or lewy or CJD or JCD or Creutzfeldt or binswanger or korsakoff or frontotemporal or FTD or VaD or "pick*1 disease").tw,kf. | * |
| 3 | 1 or 2 | * |
| 4 | exp Self-Help Devices/ or exp Internet/ or Information Technology/ or Mobile Applications/ or exp Therapy, Computer-Assisted/ or exp Microcomputers/ or exp Wearable Electronic Devices/ or exp Telemedicine/ or exp Virtual Reality/ or Augmented Reality/ | * |
| 5 | ((assistiv* or orthotic* or supportiv* or electronic* or welfare or everyday) adj2 (technolog* or device*)).tw,kf. | * |
| 6 | ("information communication technolog*" or ICT or Computer-assisted or computer-based or Web-based or "assistive technolog*").tw,kf. | * |
| 7 | (E-health or ehealth* or "e health*" or mhealth or m-health or "m health" or "mobile health" or etherap* or e-therap* or (electronic adj therap*)).tw,kf. | * |
| 8 | ("mobile phone*" or Smartphone* or "smart phone*" or "Mobile device*").tw,kf. | * |
| 9 | (Tablet or iPad* or iPhone* or "handheld device*" or handheld-device* or "handheld computer*" or handheld-computer* or microcomputer* or palmtop* or laptop* or "personal digital assist*" or PDA or "telephone application*" or touch-screen* or touchscreen* or Robot*).tw,kf. | * |
| 10 | ((mobile or software or electronic) adj1 (app or apps or application)).tw,kf. | * |
| 11 | (wearable adj2 (device* or technolog*)).tw,kf. | * |
| 12 | ((virtual or augmented) adj2 (realit* or environment* or platform* or world*)).tw,kf. | * |
| 13 | ((mixed or extended) adj2 realit*).tw,kf. | * |
| 14 | (Oculus or "Google Cardboard" or "Google Glass*" or "HTC Vive" or "Holo Lens" or "Samsung Gear" or "Magic Leap" or Wevr or NextVR or Pico or Varjo).tw,kf. | * |
| 15 | ("head* up display*" or "head-up display*" or "head mount* display*" or "head-mount* display*" or "head* worn display*" or "head-worn* display*" or HUD* or "holographic display*" or "smart glass*").tw,kf. | * |
| 16 | (telecare or "tele care" or telemedicine* or telerehabilitation* or telepsychiatry or telepsychology or teleconsultation* or remote consultation* or "tele therap*" or tele-therap* or teletherap* or videoconferenc* or "video conferenc*" or videoconsultation* or "video consultation*").tw,kf. | * |
| 17 | (artificial adj2 intelligence).tw,kf. | * |
| 18 | or/4-17 | * |
| 19 | 3 and 18 | * |
| 20 | "Treatment Adherence and Compliance"/ | * |
| 21 | (((technology or intervention) adj2 ("use" or "using")) or (adher* or adopt* or implement* or barrier or facilitator or enabl* or optimise)).kf,tw. | * |
| 22 | 20 or 21 | * |
| 23 | ("systematic review" or "Meta-Analysis").pt. | * |
| 24 | "Systematic Reviews as Topic"/ or "meta-analysis as topic"/ | * |
| 25 | ("Cochrane Database of Systematic Reviews" or evidence report technology assessment or evidence report technology assessment summary).jn. | * |
| 26 | (((comprehensive or comprehensively) adj (analysis or review or reviewed)) or ((literature or scoping) adj (search or searches))).ti,ab,kf. not "narrative review".ti. | * |
| 27 | (database or databases or cinahl or cochrane or embase or psycinfo or pubmed or medline or scopus or (web adj1 science) or ((bibliographic or literature) adj (review or reviews)) or ((electronic adj (database or databases)) or (databases adj3 searched))).ti,ab,kf. | * |
| 28 | (eligibility or excluded or exclusion or included or inclusion).ti,ab,kf. | * |
| 29 | 26 and 27 and 28 | * |
| 30 | ((comparative adj effectiveness) and (effectiveness adj review)).ti,ab,kf. | * |
| 31 | ((critical adj interpretive) and ((interpretive adj review) or (interpretive adj synthesis))).ti,ab,kf. | * |
| 32 | ((diagnostic adj test) and ((accuracy adj review) or (accuracy adj reviews) or (accuracy adj studies) or (accuracy adj study)) and (meta-analysis or scoping or systematic)).ti,ab,kf. | * |
| 33 | ((evidence adj assessment) and GRADE).ti,ab,kf. | * |
| 34 | ((evidence adj mapping) or (evidence adj review) or (exploratory adj review) or (framework adj synthesis) or (mapping adj review)).ti,ab,kf. | * |
| 35 | ((meta adj (epidemiological or ethnographic or ethnography or interpretation or narrative or review or study or synthesis or summary or theory)) or metaethnographic or metaethnography or metasynthesis).ti,ab,kf. | * |
| 36 | ((methodological or methodology) adj1 review).ti,ab,kf. | * |
| 37 | ((mixed adj methods) and (methods adj1 (review or synthesis))).ti,ab,kf. | * |
| 38 | ((narrative adj1 synthesis) or (overview adj4 reviews) or ("PRISMA" adj4 (guideline or guidelines or preferred or reporting or requirements)) or (PRISMA adj "P")).ti,ab,kf. | * |
| 39 | (((prognostic or psychometric) adj1 review) or ((qualitative adj (evidence or research)) and ((evidence or research) adj synthesis))).ti,ab,kf. | * |
| 40 | (((rapid adj evidence) and (evidence adj assessment)) or (rapid adj realist) or (rapid adj2 (review or reviews)) or (realist adj2 (review or reviews or syntheses or synthesis))).ti,ab,kf. | * |
| 41 | (((review adj economic) and (economic adj1 (evaluation or evaluations))) or ((scoping or systematic) adj2 (review or reviews or studies or study))).ti,ab,kf. | * |
| 42 | ((review adj1 reviews) or ((systematic adj evidence) and (evidence adj map)) or (systematic adj2 mapping) or (systematic adj2 literature) or (systematic adj2 (Embase or Medline or PsycInfo or PubMed)) or (systematic adj2 (review or reviews)) or ((systematical or systematically) adj2 (review or reviewed reviews)) or (systematically adj identified) or (systematized adj review) or (umbrella adj (review or reviews))).ti,ab,kf. | * |
| 43 | (meta adj2 (analyse or analyser or analyses or analysis or analytic or analytical or analytics or analyze or analyzed or analyzes)).ti,ab,kf. | * |
| 44 | (metaanalyse or Metaanalysen or metaanalyser or metaanalyses or metaanalysis* or metaanalytic or metaanalytical or metaanalytics or metaanalyze or metaanalyzed or metaanalyzes).ti,ab,kf. | * |
| 45 | "network meta-analysis"/ | * |
| 46 | (network adj1 (meta or metaanalyses or metaanalysis or metaregression)).ti,ab,kf. | * |
| 47 | (systematic and ((meta adj regression) or metagression)).ti,ab,kf. | * |
| 48 | review.pt. | * |
| 49 | (((integrated or integrative or narrative) adj (review or reviews)) or overview or ((state adj3 art) and (art adj (review or reviews)))).ti,ab,kf. not (systematic or scoping).ti. | * |
| 50 | 23 or 24 or 25 or 29 or 30 or 31 or 32 or 33 or 34 or 35 or 36 or 37 or 38 or 39 or 40 or 41 or 42 or 43 or 44 or 45 or 46 or 47 or 48 or 49 | * |
| 51 | 19 and 22 and 50 | 1832 |

CINAHL Plus <1806 to August 5, 2024>

| **#** | **Search term(s)** | **Result(s)** |
| --- | --- | --- |
| **1** | TI ( dement* or alzheimer* or lewy or CJD or JCD or Creutzfeldt or binswanger or korsakoff or frontotemporal or FTD or VaD or "pick*1 disease" ) OR SU dementia | 91,578 |
| **2** | AB ( ((assistiv* or orthotic* or supportiv* or electronic* or welfare or everyday) adj2 (technolog* or device*)) ) OR AB ( ("information communication technolog*" or ICT or Computer-assisted or computer-based or Web-based or "assistive technolog*") ) OR AB ( (E-health or ehealth* or "e health*" or mhealth or m-health or "m health" or "mobile health" or etherap* or e-therap* or (electronic adj therap*)) ) OR AB ( ("mobile phone*" or Smartphone* or "smart phone*" or "Mobile device*") ) OR AB ( (Tablet or iPad* or iPhone* or "handheld device*" or handheld-device* or "handheld computer*" or handheld-computer* or microcomputer* or palmtop* or laptop* or "personal digital assist*" or PDA or "telephone application*" or touch-screen* or touchscreen* or Robot*) ) OR AB ( ((mobile or software or electronic) adj1 (app or apps or application)) ) OR AB ( (wearable adj2 (device* or technolog*)) ) OR AB (((virtual or augmented) adj2 (realit* or environment* or platform* or world*)) ) OR AB ( ((mixed or extended) adj2 realit*) ) OR AB ((Oculus or "Google Cardboard" or "Google Glass*" or "HTC Vive" or "Holo Lens" or "Samsung Gear" or "Magic Leap" or Wevr or NextVR or Pico or Varjo) ) OR AB ( ("head* up display*" or "head-up display*" or "head mount* display*" or "head-mount* display*" or "head* worn display*" or "head-worn* display*" or HUD* or "holographic display*" or "smart glass*") ) OR AB ((telecare or "tele care" or telemedicine* or telerehabilitation* or telepsychiatry or telepsychology or teleconsultation* or remote consultation* or "tele therap*" or tele-therap* or teletherap* or videoconferenc* or "video conferenc*" or videoconsultation* or "video consultation*") ) OR AB ((artificial adj2 intelligence) ) | 82,280 |
| **3** | AB ( ((assistiv* or orthotic* or supportiv* or electronic* or welfare or everyday) adj2 (technolog* or device*)) ) OR AB ( ("information communication technolog*" or ICT or Computer-assisted or computer-based or Web-based or "assistive technolog*") ) OR AB ( (E-health or ehealth* or "e health*" or mhealth or m-health or "m health" or "mobile health" or etherap* or e-therap* or (electronic adj therap*)) ) OR AB ( ("mobile phone*" or Smartphone* or "smart phone*" or "Mobile device*") ) OR AB ( (Tablet or iPad* or iPhone* or "handheld device*" or handheld-device* or "handheld computer*" or handheld-computer* or microcomputer* or palmtop* or laptop* or "personal digital assist*" or PDA or "telephone application*" or touch-screen* or touchscreen* or Robot*) ) OR AB ( ((mobile or software or electronic) adj1 (app or apps or application)) ) OR AB ( (wearable adj2 (device* or technolog*)) ) OR AB (((virtual or augmented) adj2 (realit* or environment* or platform* or world*)) ) OR AB ( ((mixed or extended) adj2 realit*) ) OR AB ((Oculus or "Google Cardboard" or "Google Glass*" or "HTC Vive" or "Holo Lens" or "Samsung Gear" or "Magic Leap" or Wevr or NextVR or Pico or Varjo) ) OR AB ( ("head* up display*" or "head-up display*" or "head mount* display*" or "head-mount* display*" or "head* worn display*" or "head-worn* display*" or HUD* or "holographic display*" or "smart glass*") ) OR AB ((telecare or "tele care" or telemedicine* or telerehabilitation* or telepsychiatry or telepsychology or teleconsultation* or remote consultation* or "tele therap*" or tele-therap* or teletherap* or videoconferenc* or "video conferenc*" or videoconsultation* or "video consultation*") ) OR AB ((artificial adj2 intelligence) ) AND (S1 AND S2) | 593 |
| **4** | MM ( treatment adherence and compliance ) OR TX ( (((technology or intervention) adj2 ("use" or "using")) or (adher* or adopt* or implement* or barrier or facilitator or enabl* or optimise)) ) | 1,609,185 |
| **5** | (MM ( treatment adherence and compliance ) OR TX ( (((technology or intervention) adj2 ("use" or "using")) or (adher* or adopt* or implement* or barrier or facilitator or enabl* or optimise)) )) AND (S3 AND S4) | 355 |
| **6** | MM ( systematic review or meta analysis ) OR PT ( systematic review or meta analysis or literature review or narrative review or mixed methods review ) OR AB ( systematic review or meta analysis or literature review or narrative review or mixed methods review ) OR TI ( systematic review or meta analysis or literature review or narrative review or mixed methods review ) | 361,170 |
| **7** | (MM ( systematic review or meta analysis ) OR PT ( systematic review or meta analysis or literature review or narrative review or mixed methods review ) OR AB ( systematic review or meta analysis or literature review or narrative review or mixed methods review ) OR TI ( systematic review or meta analysis or literature review or narrative review or mixed methods review )) AND (S5 AND S6) | 59 |
| **8** | (MM ( systematic review or meta analysis ) OR PT ( systematic review or meta analysis or literature review or narrative review or mixed methods review ) OR AB ( systematic review or meta analysis or literature review or narrative review or mixed methods review ) OR TI ( systematic review or meta analysis or literature review or narrative review or mixed methods review )) AND (S5 AND S6)  Publication Date Limiters: 20160101-20241231 | 55 |

1. Cost-effectiveness searches

Ovid MEDLINE(R) ALL <1946 to May 22, 2024>

| **#** | **Search term(s)** | **Result(s)** |
| --- | --- | --- |
| 1 | exp Dementia/ | 215148 |
| 2 | (dement* or alzheimer* or lewy or CJD or JCD or Creutzfeldt or binswanger or korsakoff or frontotemporal or FTD or VaD or "pick*1 disease").tw,kf. | 323935 |
| 3 | 1 or 2 | 360051 |
| 4 | exp Self-Help Devices/ or exp Internet/ or Information Technology/ or Mobile Applications/ or exp Therapy, Computer-Assisted/ or exp Microcomputers/ or exp Wearable Electronic Devices/ or exp Telemedicine/ or exp Virtual Reality/ or Augmented Reality/ | 260618 |
| 5 | ((assistiv* or orthotic* or supportiv* or electronic* or welfare or everyday) adj2 (technolog* or device*)).tw,kf. | 28787 |
| 6 | ("information communication technolog*" or ICT or Computer-assisted or computer-based or Web-based or "assistive technolog*").tw,kf. | 105718 |
| 7 | (E-health or ehealth* or "e health*" or mhealth or m-health or "m health" or "mobile health" or etherap* or e-therap* or (electronic adj therap*)).tw,kf. | 27127 |
| 8 | ("mobile phone*" or Smartphone* or "smart phone*" or "Mobile device*").tw,kf. | 44946 |
| 9 | (Tablet or iPad* or iPhone* or "handheld device*" or handheld-device* or "handheld computer*" or handheld-computer* or microcomputer* or palmtop* or laptop* or "personal digital assist*" or PDA or "telephone application*" or touch-screen* or touchscreen* or Robot*).tw,kf. | 145977 |
| 10 | ((mobile or software or electronic) adj1 (app or apps or application)).tw,kf. | 11316 |
| 11 | (wearable adj2 (device* or technolog*)).tw,kf. | 12160 |
| 12 | ((virtual or augmented) adj2 (realit* or environment* or platform* or world*)).tw,kf. | 29416 |
| 13 | ((mixed or extended) adj2 realit*).tw,kf. | 1473 |
| 14 | (Oculus or "Google Cardboard" or "Google Glass*" or "HTC Vive" or "Holo Lens" or "Samsung Gear" or "Magic Leap" or Wevr or NextVR or Pico or Varjo).tw,kf. | 6473 |
| 15 | ("head* up display*" or "head-up display*" or "head mount* display*" or "head-mount* display*" or "head* worn display*" or "head-worn* display*" or HUD* or "holographic display*" or "smart glass*").tw,kf. | 7698 |
| 16 | (telecare or "tele care" or telemedicine* or telerehabilitation* or telepsychiatry or telepsychology or teleconsultation* or remote consultation* or "tele therap*" or tele-therap* or teletherap* or videoconferenc* or "video conferenc*" or videoconsultation* or "video consultation*").tw,kf. | 39010 |
| 17 | (artificial adj2 intelligence).tw,kf. | 51604 |
| 18 | or/4-17 | 630233 |
| 19 | 3 and 18 | 5685 |
| 20 | (effective* or efficacy* or superior* or benefi*).tw,kf. | 4801468 |
| 21 | (cost adj2 (effective* or benefit or utility or saving or minimization)) .tw,kf. | 212667 |
| 22 | (economic adj2 (evaluation or evaluations or analys*)).tw,kf. | 26568 |
| 23 | Randomized controlled trial.pt. | 613458 |
| 24 | Controlled clinical trial.pt. | 95535 |
| 25 | Randomized.ab. | 646284 |
| 26 | Placebo.ab. | 248440 |
| 27 | Clinical trials as topic.sh. | 202417 |
| 28 | Trial.ti. | 309700 |
| 29 | Or/20-28 | 5547324 |
| 30 | 19 and 29 | 2288 |
| 31 | ("systematic review" or "Meta-Analysis").pt. | 348225 |
| 32 | "Systematic Reviews as Topic"/ or "meta-analysis as topic"/ | 32494 |
| 33 | ("Cochrane Database of Systematic Reviews" or evidence report technology assessment or evidence report technology assessment summary).jn. | 16858 |
| 34 | (((comprehensive or comprehensively) adj (analysis or review or reviewed)) or ((literature or scoping) adj (search or searches))).ti,ab,kf. not "narrative review".ti. | 134497 |
| 35 | (database or databases or cinahl or cochrane or embase or psycinfo or pubmed or medline or scopus or (web adj1 science) or ((bibliographic or literature) adj (review or reviews)) or ((electronic adj (database or databases)) or (databases adj3 searched))).ti,ab,kf. | 1029758 |
| 36 | (eligibility or excluded or exclusion or included or inclusion).ti,ab,kf. | 2463977 |
| 37 | 34 and 35 and 36 | 37505 |
| 38 | ((comparative adj effectiveness) and (effectiveness adj review)).ti,ab,kf. | 134 |
| 39 | ((critical adj interpretive) and ((interpretive adj review) or (interpretive adj synthesis))).ti,ab,kf. | 227 |
| 40 | ((diagnostic adj test) and ((accuracy adj review) or (accuracy adj reviews) or (accuracy adj studies) or (accuracy adj study)) and (meta-analysis or scoping or systematic)).ti,ab,kf. | 650 |
| 41 | ((evidence adj assessment) and GRADE).ti,ab,kf. | 102 |
| 42 | ((evidence adj mapping) or (evidence adj review) or (exploratory adj review) or (framework adj synthesis) or (mapping adj review)).ti,ab,kf. | 3889 |
| 43 | ((meta adj (epidemiological or ethnographic or ethnography or interpretation or narrative or review or study or synthesis or summary or theory)) or metaethnographic or metaethnography or metasynthesis).ti,ab,kf. | 4253 |
| 44 | ((methodological or methodology) adj1 review).ti,ab,kf. | 3247 |
| 45 | ((mixed adj methods) and (methods adj1 (review or synthesis))).ti,ab,kf. | 474 |
| 46 | ((narrative adj1 synthesis) or (overview adj4 reviews) or ("PRISMA" adj4 (guideline or guidelines or preferred or reporting or requirements)) or (PRISMA adj "P")).ti,ab,kf. | 33679 |
| 47 | (((prognostic or psychometric) adj1 review) or ((qualitative adj (evidence or research)) and ((evidence or research) adj synthesis))).ti,ab,kf. | 867 |
| 48 | (((rapid adj evidence) and (evidence adj assessment)) or (rapid adj realist) or (rapid adj2 (review or reviews)) or (realist adj2 (review or reviews or syntheses or synthesis))).ti,ab,kf. | 4490 |
| 49 | (((review adj economic) and (economic adj1 (evaluation or evaluations))) or ((scoping or systematic) adj2 (review or reviews or studies or study))).ti,ab,kf. | 388345 |
| 50 | ((review adj1 reviews) or ((systematic adj evidence) and (evidence adj map)) or (systematic adj2 mapping) or (systematic adj2 literature) or (systematic adj2 (Embase or Medline or PsycInfo or PubMed)) or (systematic adj2 (review or reviews)) or ((systematical or systematically) adj2 (review or reviewed reviews)) or (systematically adj identified) or (systematized adj review) or (umbrella adj (review or reviews))).ti,ab,kf. | 360723 |
| 51 | (meta adj2 (analyse or analyser or analyses or analysis or analytic or analytical or analytics or analyze or analyzed or analyzes)).ti,ab,kf. | 306811 |
| 52 | (metaanalyse or Metaanalysen or metaanalyser or metaanalyses or metaanalysis* or metaanalytic or metaanalytical or metaanalytics or metaanalyze or metaanalyzed or metaanalyzes).ti,ab,kf. | 2799 |
| 53 | "network meta-analysis"/ | 6152 |
| 54 | (network adj1 (meta or metaanalyses or metaanalysis or metaregression)).ti,ab,kf. | 10844 |
| 55 | (systematic and ((meta adj regression) or metagression)).ti,ab,kf. | 9912 |
| 56 | review.pt. | 3325301 |
| 57 | (((integrated or integrative or narrative) adj (review or reviews)) or overview or ((state adj3 art) and (art adj (review or reviews)))).ti,ab,kf. not (systematic or scoping).ti. | 268749 |
| 58 | or/31-33,37-57 | 3724011 |
| 59 | 30 and 58 | 542 |

Ovid Embase(R) ALL <1947 to May 22, 2024>

| **#** | **Search term(s)** | **Result(s)** |
| --- | --- | --- |
| 1 | Exp Dementia/ | 473975 |
| 2 | (dement* or alzheimer* or lewy or CJD or JCD or Creutzfeldt or binswanger or korsakoff or frontotemporal or FTD or VaD or "pick* disease").tw,kf. | 455570 |
| 3 | 1 or 2 | 585038 |
| 4 | exp Self Help Device/ | 260618 |
| 5 | exp Internet/ | 134287 |
| 6 | Information Technology/ | 14232 |
| 7 | Mobile Application/ | 23935 |
| 8 | exp Computer Assisted therapy/ | 15512 |
| 9 | exp Microcomputer/ | 14969 |
| 10 | exp Wearable computer/ | 10455 |
| 11 | exp Telemedicine robot/ or exp/telemedicine | 77319 |
| 12 | exp Virtual Reality/ | 29138 |
| 13 | exp Augmented Reality/ | 3140 |
| 14 | ((assistiv* or orthotic* or supportiv* or electronic* or welfare or everyday) adj2 (technolog* or device*)).tw,kf. | 33422 |
| 15 | ("information communication technolog*" or ICT or Computer-assisted or computer-based or Web-based or "assistive technolog*").tw,kf. | 140490 |
| 16 | (E-health or ehealth* or "e health*" or mhealth or m-health or "m health" or "mobile health" or etherap* or e-therap* or (electronic adj therap*)).tw,kf. | 28174 |
| 17 | ("mobile phone*" or Smartphone* or "smart phone*" or "Mobile device*").tw,kf. | 54959 |
| 18 | (Tablet or iPad* or iPhone* or "handheld device*" or handheld-device* or "handheld computer*" or handheld-computer* or microcomputer* or palmtop* or laptop* or "personal digital assist*" or PDA or "telephone application*" or touch-screen* or touchscreen* or Robot*).tw,kf. | 220385 |
| 19 | ((mobile or software or electronic) adj1 (app or apps or application)).tw,kf. | 14404 |
| 20 | (wearable adj2 (device* or technolog*)).tw,kf. | 13095 |
| 21 | ((virtual or augmented) adj2 (realit* or environment* or platform* or world*)).tw,kf. | 36297 |
| 22 | ((mixed or extended) adj2 realit*).tw,kf. | 1558 |
| 23 | (Oculus or "Google Cardboard" or "Google Glass*" or "HTC Vive" or "Holo Lens" or "Samsung Gear" or "Magic Leap" or Wevr or NextVR or Pico or Varjo).tw,kf. | 8808 |
| 24 | ("head* up display*" or "head-up display*" or "head mount* display*" or "head-mount* display*" or "head* worn display*" or "head-worn* display*" or HUD* or "holographic display*" or "smart glass*").tw,kf. | 11326 |
| 25 | (telecare or "tele care" or telemedicine* or telerehabilitation* or telepsychiatry or telepsychology or teleconsultation* or remote consultation* or "tele therap*" or tele-therap* or teletherap* or videoconferenc* or "video conferenc*" or videoconsultation* or "video consultation*").tw,kf. | 51049 |
| 26 | (artificial adj2 intelligence).tw,kf. | 59881 |
| 27 | or/4-26 | 794927 |
| 28 | 3 and 27 | 9784 |
| 29 | (effective* or efficacy* or superior* or benefi*).tw,kf. | 6525467 |
| 30 | (cost adj2 (effective* or benefit or utility or saving or minimization)) .tw,kf. | 291165 |
| 31 | (economic adj2 (evaluation or evaluations or analys*)).tw,kf. | 37207 |
| 32 | Randomized controlled trial/ | 826330 |
| 33 | Controlled clinical trial/ | 473592 |
| 34 | Single blind procedure/ or double blind procedure/ | 274466 |
| 35 | Crossover procedure/ | 78516 |
| 36 | Random*.tw. | 2084714 |
| 37 | Placebo.tw. | 382620 |
| 38 | ((singl* or doubl*) adj (blind* or mask*)).tw. | 294411 |
| 39 | (crossover or cross over or factorial* or latin square).tw. | 184574 |
| 40 | (assign* or allocat* or volunteer*).tw. | 1018933 |
| 41 | Or/29-40 | 8613972 |
| 42 | 28 and 41 | 4083 |
| 43 | "systematic review"/ or "Review"/ | 348225 |
| 44 | Meta analysis/ | 32494 |
| 45 | “Systematic review (topic)”/ or “meta analysis (topic)”/ | 76470 |
| 46 | ("Cochrane Database of Systematic Reviews" or evidence report technology assessment or evidence report technology assessment summary).jn. | 17915 |
| 47 | (((comprehensive or comprehensively) adj (analysis or review or reviewed)) or ((literature or scoping) adj (search or searches))).ti,ab,kf. not "narrative review".ti. | 163061 |
| 48 | (database or databases or cinahl or cochrane or embase or psycinfo or pubmed or medline or scopus or (web adj1 science) or ((bibliographic or literature) adj (review or reviews)) or ((electronic adj (database or databases)) or (databases adj3 searched))).ti,ab,kf. | 1427383 |
| 49 | (eligibility or exclude d or exclusion or included or inclusion).ti,ab,kf. | 3881217 |
| 50 | 47 and 48 and 49 | 47327 |
| 51 | ((comparative adj effectiveness) and (effectiveness adj review)).ti,ab,kf. | 95 |
| 52 | ((critical adj interpretive) and ((interpretive adj review) or (interpretive adj synthesis))).ti,ab,kf. | 231 |
| 53 | ((diagnostic adj test) and ((accuracy adj review) or (accuracy adj reviews) or (accuracy adj studies) or (accuracy adj study)) and (meta-analysis or scoping or systematic)).ti,ab,kf. | 759 |
| 54 | ((evidence adj assessment) and GRADE).ti,ab,kf. | 122 |
| 55 | ((evidence adj mapping) or (evidence adj review) or (exploratory adj review) or (framework adj synthesis) or (mapping adj review)).ti,ab,kf. | 4412 |
| 56 | ((meta adj (epidemiological or ethnographic or ethnography or interpretation or narrative or review or study or synthesis or summary or theory)) or metaethnographic or metaethnography or metasynthesis).ti,ab,kf. | 4688 |
| 57 | ((methodological or methodology) adj1 review).ti,ab,kf. | 3732 |
| 58 | ((mixed adj methods) and (methods adj1 (review or synthesis))).ti,ab,kf. | 469 |
| 59 | ((narrative adj1 synthesis) or (overview adj4 reviews) or ("PRISMA" adj4 (guideline or guidelines or preferred or reporting or requirements)) or (PRISMA adj "P")).ti,ab,kf. | 39456 |
| 60 | (((prognostic or psychometric) adj1 review) or ((qualitative adj (evidence or research)) and ((evidence or research) adj synthesis))).ti,ab,kf. | 1040 |
| 61 | (((rapid adj evidence) and (evidence adj assessment)) or (rapid adj realist) or (rapid adj2 (review or reviews)) or (realist adj2 (review or reviews or syntheses or synthesis))).ti,ab,kf. | 5213 |
| 62 | (((review adj economic) and (economic adj1 (evaluation or evaluations))) or ((scoping or systematic) adj2 (review or reviews or studies or study))).ti,ab,kf. | 466517 |
| 63 | ((review adj1 reviews) or ((systematic adj evidence) and (evidence adj map)) or (systematic adj2 mapping) or (systematic adj2 literature) or (systematic adj2 (Embase or Medline or PsycInfo or PubMed)) or (systematic adj2 (review or reviews)) or ((systematical or systematically) adj2 (review or reviewed reviews)) or (systematically adj identified) or (systematized adj review) or (umbrella adj (review or reviews))).ti,ab,kf. | 437938 |
| 64 | (meta adj2 (analyse or analyser or analyses or analysis or analytic or analytical or analytics or analyze or analyzed or analyzes)).ti,ab,kf. | 386517 |
| 65 | (metaanalyse or Metaanalysen or metaanalyser or metaanalyses or metaanalysis* or metaanalytic or metaanalytical or metaanalytics or metaanalyze or metaanalyzed or metaanalyzes).ti,ab,kf. | 13679 |
| 66 | "network meta-analysis"/ | 9000 |
| 67 | (network adj1 (meta or metaanalyses or metaanalysis or metaregression)).ti,ab,kf. | 15030 |
| 68 | (systematic and ((meta adj regression) or metagression)).ti,ab,kf. | 11839 |
| 69 | (((integrated or integrative or narrative) adj (review or reviews)) or overview or ((state adj3 art) and (art adj (review or reviews)))).ti,ab,kf. not (systematic or scoping).ti. | 305757 |
| 70 | or/43-46,50-69 | 3775946 |
| 71 | 42 and 70 | 810 |

PsychInfo ALL <1806 to May 22, 2024>

| **#** | **Search term(s)** | **Result(s)** |
| --- | --- | --- |
| 1 | Exp Dementia/ | 96022 |
| 2 | (dement* or alzheimer* or lewy or CJD or JCD or Creutzfeldt or binswanger or korsakoff or frontotemporal or FTD or VaD or "pick* disease").tw. | 131111 |
| 3 | 1 or 2 | 131863 |
| 4 | exp Assistive technology/ | 13201 |
| 5 | exp Internet/ | 34309 |
| 6 | “Information and communication technology”/ | 11520 |
| 7 | Exp Mobile Applications/ | 3048 |
| 8 | exp Computer Assisted therapy/ | 16317 |
| 9 | exp Microcomputers/ | 1349 |
| 10 | exp Wearable devices/ | 1033 |
| 11 | exp Telemedicine/ | 15268 |
| 12 | exp Virtual Reality/ | 13114 |
| 13 | Augmented Reality/ | 1237 |
| 14 | ((assistiv* or orthotic* or supportiv* or electronic* or welfare or everyday) adj2 (technolog* or device*)).tw. | 5949 |
| 15 | ("information communication technolog*" or ICT or Computer-assisted or computer-based or Web-based or "assistive technolog*").tw. | 42238 |
| 16 | (E-health or ehealth* or "e health*" or mhealth or m-health or "m health" or "mobile health" or etherap* or e-therap* or (electronic adj therap*)).tw. | 6442 |
| 17 | ("mobile phone*" or Smartphone* or "smart phone*" or "Mobile device*").tw. | 15904 |
| 18 | (Tablet or iPad* or iPhone* or "handheld device*" or handheld-device* or "handheld computer*" or handheld-computer* or microcomputer* or palmtop* or laptop* or "personal digital assist*" or PDA or "telephone application*" or touch-screen* or touchscreen* or Robot*).tw. | 23404 |
| 19 | ((mobile or software or electronic) adj1 (app or apps or application)).tw. | 3165 |
| 20 | (wearable adj2 (device* or technolog*)).tw. | 1270 |
| 21 | ((virtual or augmented) adj2 (realit* or environment* or platform* or world*)).tw. | 17885 |
| 22 | ((mixed or extended) adj2 realit*).tw. | 472 |
| 23 | (Oculus or "Google Cardboard" or "Google Glass*" or "HTC Vive" or "Holo Lens" or "Samsung Gear" or "Magic Leap" or Wevr or NextVR or Pico or Varjo).tw. | 543 |
| 24 | ("head* up display*" or "head-up display*" or "head mount* display*" or "head-mount* display*" or "head* worn display*" or "head-worn* display*" or HUD* or "holographic display*" or "smart glass*").tw. | 3244 |
| 25 | (telecare or "tele care" or telemedicine* or telerehabilitation* or telepsychiatry or telepsychology or teleconsultation* or remote consultation* or "tele therap*" or tele-therap* or teletherap* or videoconferenc* or "video conferenc*" or videoconsultation* or "video consultation*").tw. | 8637 |
| 26 | (artificial adj2 intelligence).tw. | 9759 |
| 27 | or/4-26 | 173747 |
| 28 | 3 and 27 | 2435 |
| 29 | (effective* or efficacy* or superior* or benefi*).tw. | 937546 |
| 30 | (cost adj2 (effective* or benefit or utility or saving or minimization)).tw. | 23909 |
| 31 | (economic adj2 (evaluation or evaluations or analys*)).tw. | 4134 |
| 32 | Random*.tw. | 253729 |
| 33 | Trial*.tw. | 226075 |
| 34 | Controlled stud*.tw. | 14316 |
| 35 | Placebo.tw. | 45164 |
| 36 | ((singl* or doubl* or trebl* or tripl*) and (blind* or mask*)).tw. | 33573 |
| 37 | (cross over or crossover or factorial* or latin square).tw. | 36817 |
| 38 | (assign* or allocat* or volunteer*).tw. | 195641 |
| 39 | treatment effectiveness evaluation/ | 29735 |
| 40 | mental health program evaluation/ | 2493 |
| 41 | exp experimental design/ | 64379 |
| 42 | Or/29-41 | 1366424 |
| 43 | 28 and 42 | 1194 |
| 44 | Exp "systematic review"/ or exp "Literature review"/ | 23894 |
| 45 | Meta analysis/ | 5456 |
| 46 | ("Cochrane Database of Systematic Reviews" or evidence report technology assessment or evidence report technology assessment summary) | 587 |
| 47 | (((comprehensive or comprehensively) adj (analysis or review or reviewed)) or ((literature or scoping) adj (search or searches))).ti,ab. not "narrative review".ti. | 18499 |
| 48 | (database or databases or cinahl or cochrane or embase or psycinfo or pubmed or medline or scopus or (web adj1 science) or ((bibliographic or literature) adj (review or reviews)) or ((electronic adj (database or databases)) or (databases adj3 searched))).ti,ab. | 5326075 |
| 49 | (eligibility or exclude d or exclusion or included or inclusion).ti,ab. | 462960 |
| 50 | 47 and 48 and 49 | 6675 |
| 51 | ((comparative adj effectiveness) and (effectiveness adj review)).ti,ab. | 7 |
| 52 | ((critical adj interpretive) and ((interpretive adj review) or (interpretive adj synthesis))).ti,ab. | 117 |
| 53 | ((diagnostic adj test) and ((accuracy adj review) or (accuracy adj reviews) or (accuracy adj studies) or (accuracy adj study)) and (meta-analysis or scoping or systematic)).ti,ab. | 33 |
| 54 | ((evidence adj assessment) and GRADE).ti,ab. | 8 |
| 55 | ((evidence adj mapping) or (evidence adj review) or (exploratory adj review) or (framework adj synthesis) or (mapping adj review)).ti,ab. | 604 |
| 56 | ((meta adj (epidemiological or ethnographic or ethnography or interpretation or narrative or review or study or synthesis or summary or theory)) or metaethnographic or metaethnography or metasynthesis).ti,ab. | 2696 |
| 57 | ((methodological or methodology) adj1 review).ti,ab. | 1140 |
| 58 | ((mixed adj methods) and (methods adj1 (review or synthesis))).ti,ab. | 173 |
| 59 | ((narrative adj1 synthesis) or (overview adj4 reviews) or ("PRISMA" adj4 (guideline or guidelines or preferred or reporting or requirements)) or (PRISMA adj "P")).ti,ab. | 5870 |
| 60 | (((prognostic or psychometric) adj1 review) or ((qualitative adj (evidence or research)) and ((evidence or research) adj synthesis))).ti,ab. | 301 |
| 61 | (((rapid adj evidence) and (evidence adj assessment)) or (rapid adj realist) or (rapid adj2 (review or reviews)) or (realist adj2 (review or reviews or syntheses or synthesis))).ti,ab. | 973 |
| 62 | (((review adj economic) and (economic adj1 (evaluation or evaluations))) or ((scoping or systematic) adj2 (review or reviews or studies or study))).ti,ab. | 66377 |
| 63 | ((review adj1 reviews) or ((systematic adj evidence) and (evidence adj map)) or (systematic adj2 mapping) or (systematic adj2 literature) or (systematic adj2 (Embase or Medline or PsycInfo or PubMed)) or (systematic adj2 (review or reviews)) or ((systematical or systematically) adj2 (review or reviewed reviews)) or (systematically adj identified) or (systematized adj review) or (umbrella adj (review or reviews))).ti,ab. | 60278 |
| 64 | (meta adj2 (analyse or analyser or analyses or analysis or analytic or analytical or analytics or analyze or analyzed or analyzes)).ti,ab. | 54887 |
| 65 | (metaanalyse or Metaanalysen or metaanalyser or metaanalyses or metaanalysis* or metaanalytic or metaanalytical or metaanalytics or metaanalyze or metaanalyzed or metaanalyzes).ti,ab. | 831 |
| 66 | (network adj1 (meta or metaanalyses or metaanalysis or metaregression)).ti,ab. | 800 |
| 67 | (systematic and ((meta adj regression) or metagression)).ti,ab. | 1422 |
| 68 | (((integrated or integrative or narrative) adj (review or reviews)) or overview or ((state adj3 art) and (art adj (review or reviews)))).ti,ab. not (systematic or scoping).ti. | 90873 |
| 69 | or/44-46,50-68 | 40292 |
| 70 | 43 and 69 | 21 |

CINAHL Plus <1806 to May 22, 2024>

| **#** | **Search term(s)** | **Result(s)** |
| --- | --- | --- |
| 1 | Exp Dementia/ | 85911 |
| 2 | (dement* or alzheimer* or lewy or CJD or JCD or Creutzfeldt or binswanger or korsakoff or frontotemporal or FTD or VaD or "pick* disease") | 174537 |
| 3 | S1 or S2 | 174712 |
| 4 | MH “Assistive technology devices+” | 40102 |
| 5 | MH “Internet+” | 165961 |
| 6 | MH “Information technology+” | 23523 |
| 7 | MH “Mobile Applications” | 13650 |
| 8 | MH “Computer Assisted therapy+” | 22285 |
| 9 | MH “Microcomputers+” | 12317 |
| 10 | MH “Wearable sensors+” | 8118 |
| 11 | MH “Telemedicine+” or MH “telehealth+” | 38266 |
| 12 | MH “Virtual Reality+” | 8125 |
| 13 | MH “Augmented Reality” | 669 |
| 14 | ((assistiv* or orthotic* or supportiv* or electronic* or welfare or everyday) n2 (technolog* or device*)) | 40370 |
| 15 | ("information communication technolog*" or ICT or Computer-assisted or computer-based or Web-based or "assistive technolog*") | 165032 |
| 16 | (E-health or ehealth* or "e health*" or mhealth or m-health or "m health" or "mobile health" or etherap* or e-therap* or (electronic adj therap*)) | 46899 |
| 17 | ("mobile phone*" or Smartphone* or "smart phone*" or "Mobile device*") | 34973 |
| 18 | (Tablet or iPad* or iPhone* or "handheld device*" or handheld-device* or "handheld computer*" or handheld-computer* or microcomputer* or palmtop* or laptop* or "personal digital assist*" or PDA or "telephone application*" or touch-screen* or touchscreen* or Robot*) | 97208 |
| 19 | ((mobile or software or electronic) n1 (app or apps or application)) | 24067 |
| 20 | (wearable n2 (device* or technolog*)) | 3575 |
| 21 | ((virtual or augmented) n2 (realit* or environment* or platform* or world*)) | 22295 |
| 22 | ((mixed or extended) n2 realit*) | 612 |
| 23 | (Oculus or "Google Cardboard" or "Google Glass*" or "HTC Vive" or "Holo Lens" or "Samsung Gear" or "Magic Leap" or Wevr or NextVR or Pico or Varjo) | 10075 |
| 24 | ("head* up display*" or "head-up display*" or "head mount* display*" or "head-mount* display*" or "head* worn display*" or "head-worn* display*" or HUD* or "holographic display*" or "smart glass*") | 47723 |
| 25 | (telecare or "tele care" or telemedicine* or telerehabilitation* or telepsychiatry or telepsychology or teleconsultation* or remote consultation* or "tele therap*" or tele-therap* or teletherap* or videoconferenc* or "video conferenc*" or videoconsultation* or "video consultation*") | 50026 |
| 26 | (artificial n2 intelligence) | 24808 |
| 27 | S4 OR S5 OR S6 OR S7 OR S8 OR S9 OR S10 OR S11 OR S12 OR S13 OR S14 OR S15 OR S16 OR S17 OR S18 OR S19 OR S20 OR S21 OR S22 OR S23 OR S24 OR S25 OR S26 | 617147 |
| 28 | S3 AND S27 | 21684 |
| 29 | TI (effective* or efficacy* or superior* or benefi*) | 224474 |
| 30 | TI (cost n2 (effective* or benefit or utility or saving or minimization)) | 21352 |
| 31 | TI (economic n2 (evaluation or evaluations or analys*)) | 4363 |
| 32 | "randomi?ed controlled trial" or PT randomized controlled trial | 246991 |
| 33 | PT Clinical Trial | 114171 |
| 34 | MH Clinical Trials+ | 356063 |
| 35 | MH Random Assignment | 85131 |
| 36 | MH placebos | 14414 |
| 37 | AB (random* or trial or placebo*) or TI (random* or trial or placebo*) | 646437 |
| 38 | AB (singl* or doubl* or tripl* or trebl*) and AB (blind* or mask*) | 56103 |
| 39 | TI (singl* or doubl* or tripl* or trebl*) and TI (blind* or mask*) | 21026 |
| 40 | MH “Treatment outcomes+” | 468446 |
| 41 | S29 OR S30 OR S31 OR S32 OR S33 OR S34 OR S35 OR S36 OR S37 OR S38 OR S39 OR S40 | 1280455 |
| 42 | S28 and S41 | 5807 |
| 43 | MH "systematic review" | 134241 |
| 44 | MH “Meta analysis” | 73939 |
| 45 | ("Cochrane Database of Systematic Reviews" or evidence report technology assessment or evidence report technology assessment summary) | 18773 |
| 46 | (((comprehensive or comprehensively) n (analysis or review or reviewed)) or ((literature or scoping) n (search or searches))) | 611 |
| 47 | (database or databases or cinahl or cochrane or embase or psycinfo or pubmed or medline or scopus or (web n1 science) or ((bibliographic or literature) n (review or reviews)) or ((electronic adj (database or databases)) or (databases n3 searched))) | 482414 |
| 48 | TI (eligibility or exclude d or exclusion or included or inclusion) or AB (eligibility or exclude d or exclusion or included or inclusion) | 704178 |
| 49 | S46 and S47 and S48 | 169 |
| 50 | TI ((comparative n1 effectiveness) and (effectiveness n1 review)) or AB ((comparative n1 effectiveness) and (effectiveness n1 review)) | 68 |
| 51 | TI ((critical n1 interpretive) and ((interpretive n1 review) or (interpretive n1 synthesis))) or AB ((critical n1 interpretive) and ((interpretive n1 review) or (interpretive n1 synthesis))) | 158 |
| 52 | TI ((diagnostic n1 test) and ((accuracy n1 review) or (accuracy n1 reviews) or (accuracy n1 studies) or (accuracy n1 study)) and (meta-analysis or scoping or systematic)) OR AB ((diagnostic n1 test) and ((accuracy n1 review) or (accuracy n1 reviews) or (accuracy n1 studies) or (accuracy n1 study)) and (meta-analysis or scoping or systematic)) | 247 |
| 53 | TI ((evidence n1 assessment) and GRADE) or AB ((evidence n1 assessment) and GRADE) | 141 |
| 54 | TI ((evidence n1 mapping) or (evidence n1 review) or (exploratory n1 review) or (framework n1 synthesis) or (mapping n1 review)) or AB ((evidence n1 mapping) or (evidence n1 review) or (exploratory n1 review) or (framework n1 synthesis) or (mapping n1 review)) | 12668 |
| 55 | TI ((meta n1 (epidemiological or ethnographic or ethnography or interpretation or narrative or review or study or synthesis or summary or theory)) or metaethnographic or metaethnography or metasynthesis) or AB ((meta n1 (epidemiological or ethnographic or ethnography or interpretation or narrative or review or study or synthesis or summary or theory)) or metaethnographic or metaethnography or metasynthesis) | 66037 |
| 56 | TI ((methodological or methodology) n1 review) or AB ((methodological or methodology) n1 review) | 2556 |
| 57 | TI ((mixed n1 methods) and (methods n1 (review or synthesis))) or AB ((mixed n1 methods) and (methods n1 (review or synthesis))) | 1026 |
| 58 | TI ((narrative n1 synthesis) or (overview n4 reviews) or ("PRISMA" n4 (guideline or guidelines or preferred or reporting or requirements)) or (PRISMA n "P")) or AB ((narrative n1 synthesis) or (overview n4 reviews) or ("PRISMA" n4 (guideline or guidelines or preferred or reporting or requirements)) or (PRISMA n "P")) | 17678 |
| 59 | TI (((prognostic or psychometric) n1 review) or ((qualitative n1 (evidence or research)) and ((evidence or research) n1 synthesis))) or AB (((prognostic or psychometric) n1 review) or ((qualitative n1 (evidence or research)) and ((evidence or research) n1 synthesis))) | 781 |
| 60 | TI (((rapid n1 evidence) and (evidence n1 assessment)) or (rapid n1 realist) or (rapid n2 (review or reviews)) or (realist n2 (review or reviews or syntheses or synthesis))) or AB (((rapid n1 evidence) and (evidence n1 assessment)) or (rapid n1 realist) or (rapid n2 (review or reviews)) or (realist n2 (review or reviews or syntheses or synthesis))) | 2358 |
| 61 | TI (((review n1 economic) and (economic n1 (evaluation or evaluations))) or ((scoping or systematic) n2 (review or reviews or studies or study))) or AB (((review n1 economic) and (economic n1 (evaluation or evaluations))) or ((scoping or systematic) n2 (review or reviews or studies or study))) | 176878 |
| 62 | TI ((review n1 reviews) or ((systematic n1 evidence) and (evidence n1 map)) or (systematic n2 mapping) or (systematic n2 literature) or (systematic n2 (Embase or Medline or PsycInfo or PubMed)) or (systematic n2 (review or reviews)) or ((systematical or systematically) n2 (review or reviewed reviews)) or (systematically n1 identified) or (systematized n1 review) or (umbrella n1 (review or reviews))) or AB ((review n1 reviews) or ((systematic n1 evidence) and (evidence n1 map)) or (systematic n2 mapping) or (systematic n2 literature) or (systematic n2 (Embase or Medline or PsycInfo or PubMed)) or (systematic n2 (review or reviews)) or ((systematical or systematically) n2 (review or reviewed reviews)) or (systematically n1 identified) or (systematized n1 review) or (umbrella n1 (review or reviews))) | 170889 |
| 63 | TI (meta n2 (analyse or analyser or analyses or analysis or analytic or analytical or analytics or analyze or analyzed or analyzes)) or AB (meta n2 (analyse or analyser or analyses or analysis or analytic or analytical or analytics or analyze or analyzed or analyzes)) | 116146 |
| 64 | TI (metaanalyse or Metaanalysen or metaanalyser or metaanalyses or metaanalysis* or metaanalytic or metaanalytical or metaanalytics or metaanalyze or metaanalyzed or metaanalyzes) or AB (metaanalyse or Metaanalysen or metaanalyser or metaanalyses or metaanalysis* or metaanalytic or metaanalytical or metaanalytics or metaanalyze or metaanalyzed or metaanalyzes) | 1395 |
| 65 | TI (network n1 (meta or metaanalyses or metaanalysis or metaregression)) or AB (network n1 (meta or metaanalyses or metaanalysis or metaregression)) | 4521 |
| 66 | TI (systematic and ((meta n1 regression) or metagression)) or AB (systematic and ((meta n1 regression) or metagression)) | 2617 |
| 67 | TI (((integrated or integrative or narrative) n1 (review or reviews)) or overview or ((state n3 art) and (art n1 (review or reviews)))) or AB (((integrated or integrative or narrative) n1 (review or reviews)) or overview or ((state n3 art) and (art n1 (review or reviews)))) | 75960 |
| 68 | S43 OR S44 OR S45 OR S49 OR S50 OR S51 OR S52 OR S53 OR S54 OR S55 OR S56 OR S57 OR S58 OR S59 OR S60 OR S61 OR S62 OR S63 OR S64 OR S65 OR S66 OR S67 | 351128 |
| 69 | 42 and 68 | 1331 |
|  |  |  |
|  |  |  |
|  |  |  |
|  |  |  |
|  |  |  |

1. Ethics searches

| Ovid MEDLINE(R) ALL <1946 to May 28, 2024> | | |
| --- | --- | --- |
|  |  |  |
| 1 | exp Dementia/ | 215386 |
| 2 | (dement* or alzheimer* or lewy or CJD or JCD or Creutzfeldt or binswanger or korsakoff or frontotemporal or FTD or VaD or "pick*1 disease").tw,kf. | 324501 |
| 3 | 1 or 2 | 360627 |
| 4 | exp Self-Help Devices/ or exp Internet/ or Information Technology/ or Mobile Applications/ or exp Therapy, Computer-Assisted/ or exp Microcomputers/ or exp Wearable Electronic Devices/ or exp Telemedicine/ or exp Virtual Reality/ or Augmented Reality/ | 260974 |
| 5 | ((assistiv* or orthotic* or supportiv* or electronic* or welfare or everyday) adj2 (technolog* or device*)).tw,kf. | 28857 |
| 6 | ("information communication technolog*" or ICT or Computer-assisted or computer-based or Web-based or "assistive technolog*").tw,kf. | 105880 |
| 7 | (E-health or ehealth* or "e health*" or mhealth or m-health or "m health" or "mobile health" or etherap* or e-therap* or (electronic adj therap*)).tw,kf. | 27191 |
| 8 | ("mobile phone*" or Smartphone* or "smart phone*" or "Mobile device*").tw,kf. | 45063 |
| 9 | (Tablet or iPad* or iPhone* or "handheld device*" or handheld-device* or "handheld computer*" or handheld-computer* or microcomputer* or palmtop* or laptop* or "personal digital assist*" or PDA or "telephone application*" or touch-screen* or touchscreen* or Robot*).tw,kf. | 146275 |
| 10 | ((mobile or software or electronic) adj1 (app or apps or application)).tw,kf. | 11348 |
| 11 | (wearable adj2 (device* or technolog*)).tw,kf. | 12215 |
| 12 | ((virtual or augmented) adj2 (realit* or environment* or platform* or world*)).tw,kf. | 29516 |
| 13 | ((mixed or extended) adj2 realit*).tw,kf. | 1486 |
| 14 | (Oculus or "Google Cardboard" or "Google Glass*" or "HTC Vive" or "Holo Lens" or "Samsung Gear" or "Magic Leap" or Wevr or NextVR or Pico or Varjo).tw,kf. | 6495 |
| 15 | ("head* up display*" or "head-up display*" or "head mount* display*" or "head-mount* display*" or "head* worn display*" or "head-worn* display*" or HUD* or "holographic display*" or "smart glass*").tw,kf. | 7713 |
| 16 | (telecare or "tele care" or telemedicine* or telerehabilitation* or telepsychiatry or telepsychology or teleconsultation* or remote consultation* or "tele therap*" or tele-therap* or teletherap* or videoconferenc* or "video conferenc*" or videoconsultation* or "video consultation*").tw,kf. | 39099 |
| 17 | (artificial adj2 intelligence).tw,kf. | 52042 |
| 18 | or/4-17 | 631683 |
| 19 | 3 and 18 | 5704 |
| 20 | Ethics/ | 9952 |
| 21 | (autonomy or empower* or beneficence or non-maleficence or equity or justice or discrimination or dignity or personhood or paternalis* or identity or freedom or restraint or coercive or risk or stigma or privacy or confidential* deception or (social adj (inclusion or exclusion))).tw,kf. | 3475139 |
| 22 | 20 or 21 | 3483963 |
| 23 | 19 and 22 | 1309 |
| 24 | ("systematic review" or "Meta-Analysis").pt. | 349194 |
| 25 | Systematic Reviews as Topic/ or "meta-analysis as topic"/ | 32529 |
| 26 | ("Cochrane Database of Systematic Reviews" or evidence report technology assessment or evidence report technology assessment summary).jn. | 16875 |
| 27 | (((comprehensive or comprehensively) adj (analysis or review or reviewed)) or ((literature or scoping) adj (search or searches))).ti,ab,kf. not "narrative review".ti. | 135092 |
| 28 | (database or databases or cinahl or cochrane or embase or psycinfo or pubmed or medline or scopus or (web adj1 science) or ((bibliographic or literature) adj (review or reviews)) or ((electronic adj (database or databases)) or (databases adj3 searched))).ti,ab,kf. | 1032390 |
| 29 | (eligibility or exclude d or exclusion or included or inclusion).ti,ab,kf. | 2469191 |
| 30 | 27 and 28 and 29 | 37648 |
| 31 | ((comparative adj effectiveness) and (effectiveness adj review)).ti,ab,kf. | 134 |
| 32 | ((critical adj interpretive) and ((interpretive adj review) or (interpretive adj synthesis))).ti,ab,kf. | 227 |
| 33 | ((diagnostic adj test) and ((accuracy adj review) or (accuracy adj reviews) or (accuracy adj studies) or (accuracy adj study)) and (meta-analysis or scoping or systematic)).ti,ab,kf. | 650 |
| 34 | ((evidence adj assessment) and GRADE).ti,ab,kf. | 102 |
| 35 | ((evidence adj mapping) or (evidence adj review) or (exploratory adj review) or (framework adj synthesis) or (mapping adj review)).ti,ab,kf. | 3904 |
| 36 | ((meta adj (epidemiological or ethnographic or ethnography or interpretation or narrative or review or study or synthesis or summary or theory)) or metaethnographic or metaethnography or metasynthesis).ti,ab,kf. | 4262 |
| 37 | ((methodological or methodology) adj1 review).ti,ab,kf. | 3257 |
| 38 | ((mixed adj methods) and (methods adj1 (review or synthesis))).ti,ab,kf. | 475 |
| 39 | ((narrative adj1 synthesis) or (overview adj4 reviews) or ("PRISMA" adj4 (guideline or guidelines or preferred or reporting or requirements)) or (PRISMA adj "P")).ti,ab,kf. | 33865 |
| 40 | (((prognostic or psychometric) adj1 review) or ((qualitative adj (evidence or research)) and ((evidence or research) adj synthesis))).ti,ab,kf. | 871 |
| 41 | (((rapid adj evidence) and (evidence adj assessment)) or (rapid adj realist) or (rapid adj2 (review or reviews)) or (realist adj2 (review or reviews or syntheses or synthesis))).ti,ab,kf. | 4513 |
| 42 | (((review adj economic) and (economic adj1 (evaluation or evaluations))) or ((scoping or systematic) adj2 (review or reviews or studies or study))).ti,ab,kf. | 389711 |
| 43 | ((review adj1 reviews) or ((systematic adj evidence) and (evidence adj map)) or (systematic adj2 mapping) or (systematic adj2 literature) or (systematic adj2 (Embase or Medline or PsycInfo or PubMed)) or (systematic adj2 (review or reviews)) or ((systematical or systematically) adj2 (review or reviewed reviews)) or (systematically adj identified) or (systematized adj review) or (umbrella adj (review or reviews))).ti,ab,kf. | 361911 |
| 44 | (meta adj2 (analyse or analyser or analyses or analysis or analytic or analytical or analytics or analyze or analyzed or analyzes)).ti,ab,kf. | 307705 |
| 45 | (metaanalyse or Metaanalysen or metaanalyser or metaanalyses or metaanalysis* or metaanalytic or metaanalytical or metaanalytics or metaanalyze or metaanalyzed or metaanalyzes).ti,ab,kf. | 2804 |
| 46 | network meta-analysis/ | 6192 |
| 47 | (network adj1 (meta or metaanalyses or metaanalysis or metaregression)).ti,ab,kf. | 10888 |
| 48 | (systematic and ((meta adj regression) or metagression)).ti,ab,kf. | 9959 |
| 49 | review.pt. | 3330262 |
| 50 | (((integrated or integrative or narrative) adj (review or reviews)) or overview or ((state adj3 art) and (art adj (review or reviews)))).ti,ab,kf. not (systematic or scoping).ti. | 269522 |
| 51 | or/24-26,30-50 | 3731331 |
| 52 | 23 and 51 | 336 |
| 53 | limit 52 to yr="2016 -Current" | 294 |

Ovid Embase(R) ALL <1947 to May 22, 2024>

| Embase Classic+Embase <1947 to 2024 May 28> | | |
| --- | --- | --- |
|  |  |  |
| 1 | exp Dementia/ | 474355 |
| 2 | (dement* or alzheimer* or lewy or CJD or JCD or Creutzfeldt or binswanger or korsakoff or frontotemporal or FTD or VaD or "pick* disease").tw,kf. | 455948 |
| 3 | 1 or 2 | 585525 |
| 4 | exp Self Help Device/ | 3373 |
| 5 | exp Internet/ | 134395 |
| 6 | Information Technology/ | 14253 |
| 7 | Mobile Application/ | 23984 |
| 8 | exp Computer Assisted therapy/ | 15513 |
| 9 | exp Microcomputer/ | 14969 |
| 10 | exp Wearable computer/ | 10501 |
| 11 | exp telemedicine robot/ or exp telemedicine/ | 77476 |
| 12 | exp Virtual Reality/ | 29181 |
| 13 | exp Augmented Reality/ | 3157 |
| 14 | ((assistiv* or orthotic* or supportiv* or electronic* or welfare or everyday) adj2 (technolog* or device*)).tw,kf. | 33442 |
| 15 | ("information communication technolog*" or ICT or Computer-assisted or computer-based or Web-based or "assistive technolog*").tw,kf. | 140598 |
| 16 | (E-health or ehealth* or "e health*" or mhealth or m-health or "m health" or "mobile health" or etherap* or e-therap* or (electronic adj therap*)).tw,kf. | 28218 |
| 17 | ("mobile phone*" or Smartphone* or "smart phone*" or "Mobile device*").tw,kf. | 55060 |
| 18 | (Tablet or iPad* or iPhone* or "handheld device*" or handheld-device* or "handheld computer*" or handheld-computer* or microcomputer* or palmtop* or laptop* or "personal digital assist*" or PDA or "telephone application*" or touch-screen* or touchscreen* or Robot*).tw,kf. | 220738 |
| 19 | ((mobile or software or electronic) adj1 (app or apps or application)).tw,kf. | 14440 |
| 20 | (wearable adj2 (device* or technolog*)).tw,kf. | 13132 |
| 21 | ((virtual or augmented) adj2 (realit* or environment* or platform* or world*)).tw,kf. | 36364 |
| 22 | ((mixed or extended) adj2 realit*).tw,kf. | 1562 |
| 23 | (Oculus or "Google Cardboard" or "Google Glass*" or "HTC Vive" or "Holo Lens" or "Samsung Gear" or "Magic Leap" or Wevr or NextVR or Pico or Varjo).tw,kf. | 8820 |
| 24 | ("head* up display*" or "head-up display*" or "head mount* display*" or "head-mount* display*" or "head* worn display*" or "head-worn* display*" or HUD* or "holographic display*" or "smart glass*").tw,kf. | 11340 |
| 25 | (telecare or "tele care" or telemedicine* or telerehabilitation* or telepsychiatry or telepsychology or teleconsultation* or remote consultation* or "tele therap*" or tele-therap* or teletherap* or videoconferenc* or "video conferenc*" or videoconsultation* or "video consultation*").tw,kf. | 51153 |
| 26 | (artificial adj2 intelligence).tw,kf. | 60210 |
| 27 | or/4-26 | 796254 |
| 28 | 3 and 27 | 9801 |
| 29 | ethics/ | 92536 |
| 30 | (autonomy or empower* or beneficence or non-maleficence or equity or justice or discrimination or dignity or personhood or paternalis* or identity or freedom or restraint or coercive or risk or stigma or privacy or confidential* or deception or (social adj (inclusion or exclusion))).tw,kf. | 4930693 |
| 31 | 29 or 30 | 5006096 |
| 32 | 28 and 31 | 2256 |
| 33 | ("Cochrane Database of Systematic Reviews" or evidence report technology assessment or evidence report technology assessment summary).jn. | 17928 |
| 34 | (((comprehensive or comprehensively) adj (analysis or review or reviewed)) or ((literature or scoping) adj (search or searches))).ti,ab,kf. not "narrative review".ti. | 163380 |
| 35 | (database or databases or cinahl or cochrane or embase or psycinfo or pubmed or medline or scopus or (web adj1 science) or ((bibliographic or literature) adj (review or reviews)) or ((electronic adj (database or databases)) or (databases adj3 searched))).ti,ab,kf. | 1429396 |
| 36 | (eligibility or exclude d or exclusion or included or inclusion).ti,ab,kf. | 3886020 |
| 37 | systematic review/ or "Review"/ | 3327176 |
| 38 | Meta analysis/ | 317357 |
| 39 | Systematic review (topic)/ or "meta analysis (topic)"/ | 76528 |
| 40 | 34 and 35 and 36 | 47398 |
| 41 | ((comparative adj effectiveness) and (effectiveness adj review)).ti,ab,kf. | 95 |
| 42 | ((critical adj interpretive) and ((interpretive adj review) or (interpretive adj synthesis))).ti,ab,kf. | 231 |
| 43 | ((diagnostic adj test) and ((accuracy adj review) or (accuracy adj reviews) or (accuracy adj studies) or (accuracy adj study)) and (meta-analysis or scoping or systematic)).ti,ab,kf. | 762 |
| 44 | ((evidence adj assessment) and GRADE).ti,ab,kf. | 122 |
| 45 | ((evidence adj mapping) or (evidence adj review) or (exploratory adj review) or (framework adj synthesis) or (mapping adj review)).ti,ab,kf. | 4420 |
| 46 | ((meta adj (epidemiological or ethnographic or ethnography or interpretation or narrative or review or study or synthesis or summary or theory)) or metaethnographic or metaethnography or metasynthesis).ti,ab,kf. | 4702 |
| 47 | ((methodological or methodology) adj1 review).ti,ab,kf. | 3740 |
| 48 | ((mixed adj methods) and (methods adj1 (review or synthesis))).ti,ab,kf. | 471 |
| 49 | ((narrative adj1 synthesis) or (overview adj4 reviews) or ("PRISMA" adj4 (guideline or guidelines or preferred or reporting or requirements)) or (PRISMA adj "P")).ti,ab,kf. | 39560 |
| 50 | (((prognostic or psychometric) adj1 review) or ((qualitative adj (evidence or research)) and ((evidence or research) adj synthesis))).ti,ab,kf. | 1040 |
| 51 | (((rapid adj evidence) and (evidence adj assessment)) or (rapid adj realist) or (rapid adj2 (review or reviews)) or (realist adj2 (review or reviews or syntheses or synthesis))).ti,ab,kf. | 5230 |
| 52 | (((review adj economic) and (economic adj1 (evaluation or evaluations))) or ((scoping or systematic) adj2 (review or reviews or studies or study))).ti,ab,kf. | 467262 |
| 53 | ((review adj1 reviews) or ((systematic adj evidence) and (evidence adj map)) or (systematic adj2 mapping) or (systematic adj2 literature) or (systematic adj2 (Embase or Medline or PsycInfo or PubMed)) or (systematic adj2 (review or reviews)) or ((systematical or systematically) adj2 (review or reviewed reviews)) or (systematically adj identified) or (systematized adj review) or (umbrella adj (review or reviews))).ti,ab,kf. | 438603 |
| 54 | (meta adj2 (analyse or analyser or analyses or analysis or analytic or analytical or analytics or analyze or analyzed or analyzes)).ti,ab,kf. | 387086 |
| 55 | (metaanalyse or Metaanalysen or metaanalyser or metaanalyses or metaanalysis* or metaanalytic or metaanalytical or metaanalytics or metaanalyze or metaanalyzed or metaanalyzes).ti,ab,kf. | 13683 |
| 56 | network meta-analysis/ | 9034 |
| 57 | (network adj1 (meta or metaanalyses or metaanalysis or metaregression)).ti,ab,kf. | 15081 |
| 58 | (systematic and ((meta adj regression) or metagression)).ti,ab,kf. | 11858 |
| 59 | (((integrated or integrative or narrative) adj (review or reviews)) or overview or ((state adj3 art) and (art adj (review or reviews)))).ti,ab,kf. not (systematic or scoping).ti. | 306067 |
| 60 | or/33,36-59 | 7130153 |
| 61 | 32 and 60 | 936 |
| 62 | limit 61 to yr="2016 -Current" | 744 |

PsychInfo ALL <1806 to May 22, 2024>

| APA PsycInfo <1806 to May Week 4 2024> | | |
| --- | --- | --- |
|  |  |  |
| 1 | exp Dementia/ | 96022 |
| 2 | (dement* or alzheimer* or lewy or CJD or JCD or Creutzfeldt or binswanger or korsakoff or frontotemporal or FTD or VaD or "pick* disease").tw. | 131111 |
| 3 | 1 or 2 | 131863 |
| 4 | exp Assistive technology/ | 13201 |
| 5 | exp Internet/ | 34309 |
| 6 | Information and communication technology/ | 11520 |
| 7 | exp Mobile Applications/ | 3048 |
| 8 | exp Computer Assisted therapy/ | 16317 |
| 9 | exp Microcomputers/ | 1349 |
| 10 | exp Wearable devices/ | 1033 |
| 11 | exp Telemedicine/ | 15268 |
| 12 | exp Virtual Reality/ | 13114 |
| 13 | Augmented Reality/ | 1237 |
| 14 | ((assistiv* or orthotic* or supportiv* or electronic* or welfare or everyday) adj2 (technolog* or device*)).tw. | 5949 |
| 15 | ("information communication technolog*" or ICT or Computer-assisted or computer-based or Web-based or "assistive technolog*").tw. | 42238 |
| 16 | (E-health or ehealth* or "e health*" or mhealth or m-health or "m health" or "mobile health" or etherap* or e-therap* or (electronic adj therap*)).tw. | 6442 |
| 17 | ("mobile phone*" or Smartphone* or "smart phone*" or "Mobile device*").tw. | 15904 |
| 18 | (Tablet or iPad* or iPhone* or "handheld device*" or handheld-device* or "handheld computer*" or handheld-computer* or microcomputer* or palmtop* or laptop* or "personal digital assist*" or PDA or "telephone application*" or touch-screen* or touchscreen* or Robot*).tw. | 23404 |
| 19 | ((mobile or software or electronic) adj1 (app or apps or application)).tw. | 3165 |
| 20 | (wearable adj2 (device* or technolog*)).tw. | 1270 |
| 21 | ((virtual or augmented) adj2 (realit* or environment* or platform* or world*)).tw. | 17885 |
| 22 | ((mixed or extended) adj2 realit*).tw. | 472 |
| 23 | (Oculus or "Google Cardboard" or "Google Glass*" or "HTC Vive" or "Holo Lens" or "Samsung Gear" or "Magic Leap" or Wevr or NextVR or Pico or Varjo).tw. | 543 |
| 24 | ("head* up display*" or "head-up display*" or "head mount* display*" or "head-mount* display*" or "head* worn display*" or "head-worn* display*" or HUD* or "holographic display*" or "smart glass*").tw. | 3244 |
| 25 | (telecare or "tele care" or telemedicine* or telerehabilitation* or telepsychiatry or telepsychology or teleconsultation* or remote consultation* or "tele therap*" or tele-therap* or teletherap* or videoconferenc* or "video conferenc*" or videoconsultation* or "video consultation*").tw. | 8637 |
| 26 | (artificial adj2 intelligence).tw. | 9759 |
| 27 | or/4-26 | 173747 |
| 28 | 3 and 27 | 2435 |
| 29 | Ethics/ | 19816 |
| 30 | (autonomy or empower* or beneficence or non-maleficence or equity or justice or discrimination or dignity or personhood or paternalis* or identity or freedom or restraint or coercive or risk or stigma or privacy or confidential* or deception or (social adj (inclusion or exclusion))).tw. | 884124 |
| 31 | 29 or 30 | 898636 |
| 32 | 28 and 31 | 612 |
| 33 | exp "systematic review"/ or exp "Literature review"/ | 23894 |
| 34 | Meta analysis/ | 5456 |
| 35 | ("Cochrane Database of Systematic Reviews" or evidence report technology assessment or evidence report technology assessment summary).mp. [mp=title, abstract, heading word, table of contents, key concepts, original title, tests & measures, mesh word] | 587 |
| 36 | (((comprehensive or comprehensively) adj (analysis or review or reviewed)) or ((literature or scoping) adj (search or searches))).ti,ab. not "narrative review".ti. | 18499 |
| 37 | (database or databases or cinahl or cochrane or embase or psycinfo or pubmed or medline or scopus or (web adj1 science) or ((bibliographic or literature) adj (review or reviews)) or ((electronic adj (database or databases)) or (databases adj3 searched))).ti,ab. | 5326075 |
| 38 | (eligibility or exclude d or exclusion or included or inclusion).ti,ab. | 462960 |
| 39 | 36 and 37 and 38 | 6675 |
| 40 | ((comparative adj effectiveness) and (effectiveness adj review)).ti,ab. | 7 |
| 41 | ((critical adj interpretive) and ((interpretive adj review) or (interpretive adj synthesis))).ti,ab. | 117 |
| 42 | ((diagnostic adj test) and ((accuracy adj review) or (accuracy adj reviews) or (accuracy adj studies) or (accuracy adj study)) and (meta-analysis or scoping or systematic)).ti,ab. | 33 |
| 43 | ((evidence adj assessment) and GRADE).ti,ab. | 8 |
| 44 | ((evidence adj mapping) or (evidence adj review) or (exploratory adj review) or (framework adj synthesis) or (mapping adj review)).ti,ab. | 604 |
| 45 | ((meta adj (epidemiological or ethnographic or ethnography or interpretation or narrative or review or study or synthesis or summary or theory)) or metaethnographic or metaethnography or metasynthesis).ti,ab. | 2696 |
| 46 | ((methodological or methodology) adj1 review).ti,ab. | 1140 |
| 47 | ((mixed adj methods) and (methods adj1 (review or synthesis))).ti,ab. | 173 |
| 48 | ((narrative adj1 synthesis) or (overview adj4 reviews) or ("PRISMA" adj4 (guideline or guidelines or preferred or reporting or requirements)) or (PRISMA adj "P")).ti,ab. | 5870 |
| 49 | (((prognostic or psychometric) adj1 review) or ((qualitative adj (evidence or research)) and ((evidence or research) adj synthesis))).ti,ab. | 301 |
| 50 | (((rapid adj evidence) and (evidence adj assessment)) or (rapid adj realist) or (rapid adj2 (review or reviews)) or (realist adj2 (review or reviews or syntheses or synthesis))).ti,ab. | 973 |
| 51 | (((review adj economic) and (economic adj1 (evaluation or evaluations))) or ((scoping or systematic) adj2 (review or reviews or studies or study))).ti,ab. | 66377 |
| 52 | ((review adj1 reviews) or ((systematic adj evidence) and (evidence adj map)) or (systematic adj2 mapping) or (systematic adj2 literature) or (systematic adj2 (Embase or Medline or PsycInfo or PubMed)) or (systematic adj2 (review or reviews)) or ((systematical or systematically) adj2 (review or reviewed reviews)) or (systematically adj identified) or (systematized adj review) or (umbrella adj (review or reviews))).ti,ab. | 60278 |
| 53 | (meta adj2 (analyse or analyser or analyses or analysis or analytic or analytical or analytics or analyze or analyzed or analyzes)).ti,ab. | 54887 |
| 54 | (metaanalyse or Metaanalysen or metaanalyser or metaanalyses or metaanalysis* or metaanalytic or metaanalytical or metaanalytics or metaanalyze or metaanalyzed or metaanalyzes).ti,ab. | 831 |
| 55 | (network adj1 (meta or metaanalyses or metaanalysis or metaregression)).ti,ab. | 800 |
| 56 | (systematic and ((meta adj regression) or metagression)).ti,ab. | 1422 |
| 57 | (((integrated or integrative or narrative) adj (review or reviews)) or overview or ((state adj3 art) and (art adj (review or reviews)))).ti,ab. not (systematic or scoping).ti. | 90873 |
| 58 | or/33-35,39-57 | 219908 |
| 59 | 32 and 58 | 94 |

CINAHL Plus <1806 to May 22, 2024>

| **#** | **Query** | **Results** |
| --- | --- | --- |
| S60 | S58 AND S32 | 446 |
|  |  |  |
|  |  |  |
| S59 | S58 AND S32 | 595 |
|  |  |  |
|  |  |  |
| S58 | S33 OR S34 OR S35 OR S39 OR S40 OR S41 OR S42 OR S43 OR S44 OR S45 OR S46 OR S47 OR S48 OR S49 OR S50 OR S51 OR S52 OR S53 OR S54 OR S55 OR S56 OR S57 | 351,607 |
|  |  |  |
|  |  |  |
| S57 | TI (((integrated or integrative or narrative) n1 (review or reviews)) or overview or ((state n3 art) and (art n1 (review or reviews)))) or AB (((integrated or integrative or narrative) n1 (review or reviews)) or overview or ((state n3 art) and (art n1 (review or reviews)))) | 76,021 |
|  |  |  |
|  |  |  |
| S56 | TI (systematic and ((meta n1 regression) or metagression)) or AB (systematic and ((meta n1 regression) or metagression)) | 2,619 |
|  |  |  |
|  |  |  |
| S55 | TI (network n1 (meta or metaanalyses or metaanalysis or metaregression)) or AB (network n1 (meta or metaanalyses or metaanalysis or metaregression)) | 4,535 |
|  |  |  |
|  |  |  |
| S54 | TI (metaanalyse or Metaanalysen or metaanalyser or metaanalyses or metaanalysis* or metaanalytic or metaanalytical or metaanalytics or metaanalyze or metaanalyzed or metaanalyzes) or AB (metaanalyse or Metaanalysen or metaanalyser or metaanalyses or metaanalysis* or metaanalytic or metaanalytical or metaanalytics or metaanalyze or metaanalyzed or metaanalyzes) | 1,396 |
|  |  |  |
|  |  |  |
| S53 | TI (meta n2 (analyse or analyser or analyses or analysis or analytic or analytical or analytics or analyze or analyzed or analyzes)) or AB (meta n2 (analyse or analyser or analyses or analysis or analytic or analytical or analytics or analyze or analyzed or analyzes)) | 116,337 |
|  |  |  |
|  |  |  |
| S52 | TI ((review n1 reviews) or ((systematic n1 evidence) and (evidence n1 map)) or (systematic n2 mapping) or (systematic n2 literature) or (systematic n2 (Embase or Medline or PsycInfo or PubMed)) or (systematic n2 (review or reviews)) or ((systematical or systematically) n2 (review or reviewed reviews)) or (systematically n1 identified) or (systematized n1 review) or (umbrella n1 (review or reviews))) or AB ((review n1 reviews) or ((systematic n1 evidence) and (evidence n1 map)) or (systematic n2 mapping) or (systematic n2 literature) or (systematic n2 (Embase or Medline or PsycInfo or PubMed)) or (systematic n2 (review or reviews)) or ((systematical or systematically) n2 (review or reviewed reviews)) or (systematically n1 identified) or (systematized n1 review) or (umbrella n1 (review or reviews))) | 171,176 |
|  |  |  |
|  |  |  |
| S51 | TI (((review n1 economic) and (economic n1 (evaluation or evaluations))) or ((scoping or systematic) n2 (review or reviews or studies or study))) or AB (((review n1 economic) and (economic n1 (evaluation or evaluations))) or ((scoping or systematic) n2 (review or reviews or studies or study))) | 177,213 |
|  |  |  |
|  |  |  |
| S50 | TI (((rapid n1 evidence) and (evidence n1 assessment)) or (rapid n1 realist) or (rapid n2 (review or reviews)) or (realist n2 (review or reviews or syntheses or synthesis))) or AB (((rapid n1 evidence) and (evidence n1 assessment)) or (rapid n1 realist) or (rapid n2 (review or reviews)) or (realist n2 (review or reviews or syntheses or synthesis))) | 2,362 |
|  |  |  |
|  |  |  |
| S49 | TI (((prognostic or psychometric) n1 review) or ((qualitative n1 (evidence or research)) and ((evidence or research) n1 synthesis))) or AB (((prognostic or psychometric) n1 review) or ((qualitative n1 (evidence or research)) and ((evidence or research) n1 synthesis))) | 783 |
|  |  |  |
|  |  |  |
| S48 | TI ((narrative n1 synthesis) or (overview n4 reviews) or ("PRISMA" n4 (guideline or guidelines or preferred or reporting or requirements)) or (PRISMA n "P")) or AB ((narrative n1 synthesis) or (overview n4 reviews) or ("PRISMA" n4 (guideline or guidelines or preferred or reporting or requirements)) or (PRISMA n "P")) | 17,710 |
|  |  |  |
|  |  |  |
| S47 | TI ((mixed n1 methods) and (methods n1 (review or synthesis))) or AB ((mixed n1 methods) and (methods n1 (review or synthesis))) | 1,028 |
|  |  |  |
|  |  |  |
| S46 | TI ((methodological or methodology) n1 review) or AB ((methodological or methodology) n1 review) | 2,556 |
|  |  |  |
|  |  |  |
| S45 | TI ((meta n1 (epidemiological or ethnographic or ethnography or interpretation or narrative or review or study or synthesis or summary or theory)) or metaethnographic or metaethnography or metasynthesis) or AB ((meta n1 (epidemiological or ethnographic or ethnography or interpretation or narrative or review or study or synthesis or summary or theory)) or metaethnographic or metaethnography or metasynthesis) | 66,170 |
|  |  |  |
|  |  |  |
| S44 | TI ((evidence n1 mapping) or (evidence n1 review) or (exploratory n1 review) or (framework n1 synthesis) or (mapping n1 review)) or AB ((evidence n1 mapping) or (evidence n1 review) or (exploratory n1 review) or (framework n1 synthesis) or (mapping n1 review)) | 12,674 |
|  |  |  |
|  |  |  |
| S43 | TI ((evidence n1 assessment) and GRADE) or AB ((evidence n1 assessment) and GRADE) | 141 |
|  |  |  |
|  |  |  |
| S42 | TI ((diagnostic n1 test) and ((accuracy n1 review) or (accuracy n1 reviews) or (accuracy n1 studies) or (accuracy n1 study)) and (meta-analysis or scoping or systematic)) OR AB ((diagnostic n1 test) and ((accuracy n1 review) or (accuracy n1 reviews) or (accuracy n1 studies) or (accuracy n1 study)) and (meta-analysis or scoping or systematic)) | 247 |
|  |  |  |
|  |  |  |
| S41 | TI ((critical n1 interpretive) and ((interpretive n1 review) or (interpretive n1 synthesis))) or AB ((critical n1 interpretive) and ((interpretive n1 review) or (interpretive n1 synthesis))) | 158 |
|  |  |  |
|  |  |  |
| S40 | TI ((comparative n1 effectiveness) and (effectiveness n1 review)) or AB ((comparative n1 effectiveness) and (effectiveness n1 review)) | 68 |
|  |  |  |
|  |  |  |
| S39 | S36 AND S37 AND S38 | 169 |
|  |  |  |
|  |  |  |
| S38 | TI (eligibility or exclude d or exclusion or included or inclusion) or AB (eligibility or exclude d or exclusion or included or inclusion) | 704,685 |
|  |  |  |
|  |  |  |
| S37 | (database or databases or cinahl or cochrane or embase or psycinfo or pubmed or medline or scopus or (web n1 science) or ((bibliographic or literature) n (review or reviews)) or ((electronic adj (database or databases)) or (databases n3 searched))) | 482,752 |
|  |  |  |
|  |  |  |
| S36 | (((comprehensive or comprehensively) n (analysis or review or reviewed)) or ((literature or scoping) n (search or searches))) | 611 |
|  |  |  |
|  |  |  |
| S35 | ("Cochrane Database of Systematic Reviews" or evidence report technology assessment or evidence report technology assessment summary) | 18,779 |
|  |  |  |
|  |  |  |
| S34 | MH “Meta analysis” | 74,010 |
|  |  |  |
|  |  |  |
| S33 | MH "systematic review" | 134,419 |
|  |  |  |
|  |  |  |
| S32 | S28 AND S31 | 3,646 |
|  |  |  |
|  |  |  |
| S31 | S29 OR S30 | 1,118,974 |
|  |  |  |
|  |  |  |
| S30 | TI (autonomy or empower* or beneficence or non-maleficence or equity or justice or discrimination or dignity or personhood or paternalis* or identity or freedom or restraint or coercive or risk or stigma or privacy or confidential* or deception or (social n (inclusion or exclusion))) OR AB (autonomy or empower* or beneficence or non-maleficence or equity or justice or discrimination or dignity or personhood or paternalis* or identity or freedom or restraint or coercive or risk or stigma or privacy or confidential* or deception or (social n (inclusion or exclusion))) | 1,107,444 |
|  |  |  |
|  |  |  |
| S29 | (MH "Ethics") | 13,497 |
|  |  |  |
|  |  |  |
| S28 | S3 AND S27 | 21,965 |
|  |  |  |
|  |  |  |
| S27 | S4 OR S5 OR S6 OR S7 OR S8 OR S9 OR S10 OR S11 OR S12 OR S13 OR S14 OR S15 OR S16 OR S17 OR S18 OR S19 OR S20 OR S21 OR S22 OR S23 OR S24 OR S25 OR S26 | 616,448 |
|  |  |  |
|  |  |  |
| S26 | (artificial n2 intelligence) | 24,913 |
|  |  |  |
|  |  |  |
| S25 | (telecare or "tele care" or telemedicine* or telerehabilitation* or telepsychiatry or telepsychology or teleconsultation* or remote consultation* or "tele therap*" or tele-therap* or teletherap* or videoconferenc* or "video conferenc*" or videoconsultation* or "video consultation*") | 50,105 |
|  |  |  |
|  |  |  |
| S24 | ("head* up display*" or "head-up display*" or "head mount* display*" or "head-mount* display*" or "head* worn display*" or "head-worn* display*" or HUD* or "holographic display*" or "smart glass*") | 47,764 |
|  |  |  |
|  |  |  |
| S23 | (Oculus or "Google Cardboard" or "Google Glass*" or "HTC Vive" or "Holo Lens" or "Samsung Gear" or "Magic Leap" or Wevr or NextVR or Pico or Varjo) | 10,082 |
|  |  |  |
|  |  |  |
| S22 | ((mixed or extended) n2 realit*) | 616 |
|  |  |  |
|  |  |  |
| S21 | ((virtual or augmented) n2 (realit* or environment* or platform* or world*)) | 22,187 |
|  |  |  |
|  |  |  |
| S20 | (wearable n2 (device* or technolog*)) | 3,582 |
|  |  |  |
|  |  |  |
| S19 | ((mobile or software or electronic) n1 (app or apps or application)) | 24,101 |
|  |  |  |
|  |  |  |
| S18 | (Tablet or iPad* or iPhone* or "handheld device*" or handheld-device* or "handheld computer*" or handheld-computer* or microcomputer* or palmtop* or laptop* or "personal digital assist*" or PDA or "telephone application*" or touch-screen* or touchscreen* or Robot*) | 97,258 |
|  |  |  |
|  |  |  |
| S17 | ("mobile phone*" or Smartphone* or "smart phone*" or "Mobile device*") | 35,010 |
|  |  |  |
|  |  |  |
| S16 | (E-health or ehealth* or "e health*" or mhealth or m-health or "m health" or "mobile health" or etherap* or e-therap* or (electronic adj therap*)) | 46,954 |
|  |  |  |
|  |  |  |
| S15 | ("information communication technolog*" or ICT or Computer-assisted or computer-based or Web-based or "assistive technolog*") | 165,095 |
|  |  |  |
|  |  |  |
| S14 | ((assistiv* or orthotic* or supportiv* or electronic* or welfare or everyday) n2 (technolog* or device*)) | 40,401 |
|  |  |  |
|  |  |  |
| S13 | MH “Augmented Reality” | 673 |
|  |  |  |
|  |  |  |
| S12 | MH “Virtual Reality+” | 7,971 |
|  |  |  |
|  |  |  |
| S11 | MH “Telemedicine+” or MH “telehealth+” | 38,331 |
|  |  |  |
|  |  |  |
| S10 | MH “Wearable sensors+” | 8,141 |
|  |  |  |
|  |  |  |
| S9 | MH “Microcomputers+” | 12,328 |
|  |  |  |
|  |  |  |
| S8 | MH “Computer Assisted therapy+” | 35,902 |
|  |  |  |
|  |  |  |
| S7 | MH “Mobile Applications” | 13,675 |
|  |  |  |
|  |  |  |
| S6 | MH “Information technology+” | 23,559 |
|  |  |  |
|  |  |  |
| S5 | MH “Internet+” | 166,105 |
|  |  |  |
|  |  |  |
| S4 | MH “Assistive technology devices+” | 40,119 |
|  |  |  |
|  |  |  |
| S3 | S1 OR S2 | 174,819 |
|  |  |  |
|  |  |  |
| S2 | (dement* or alzheimer* or lewy or CJD or JCD or Creutzfeldt or binswanger or korsakoff or frontotemporal or FTD or VaD or "pick* disease") | 174,644 |
|  |  |  |
|  |  |  |
| S1 | (MH "Dementia+") | 85,952 |
|  |  |  |
|  |  |  |
