# Supplementary material for: Digital assistive technologies for community-dwelling people with dementia: A systematic review of systematic reviews by the INTERDEM AI & assistive technology taskforce
Source: Digit Health. 2025 Aug 3;11:20552076251362353. doi: 10.1177/20552076251362353 (PMC12319280; doi:10.1177/20552076251362353)
Supplement: sj-docx-2-dhj-10.1177_20552076251362353 - Supplemental material for Digital assistive technologies for community-dwelling people with dementia: A systematic review of systematic reviews by the INTERDEM AI & assistive technology taskforce [file sj-docx-2-dhj-10.1177_20552076251362353.docx]

**Development theme: Identification of studies via databases and registers**

Records identified from databases

(n= 1,327)

Records removed *before screening*:

Duplicate records removed (n= 226)

**Identification**

Records screened

(n = 1,101)

Records excluded (n= 1,010)

Reports sought for retrieval

(n= 91)

Reports not retrieved (n= 0)

**Screening**

Reports excluded: (n= 44)

Wrong population (n=19)

No or wrong technology (n=6)

Wrong publication type (n=10)

No development reported (n=8)

No report in English (n=1)

Reports assessed for eligibility

(n= 91)

Studies included in review

(n= 47)

**Included**

**Usability theme: identification of studies via databases and registers**

Records identified from databases

(n= 488)

Records removed *before screening*:

Duplicate records removed (n= 204)

**Identification**

Records screened

(n = 284)

Records excluded (n= 226)

Reports sought for retrieval

(n= 58)

Reports not retrieved (n= 1)

**Screening**

Reports assessed for eligibility

(n= 57)

Reports excluded: (n= 18)

Wrong publication type (n= 15)

No usability reported (n= 3)

Studies included in review

(n= 39)

**Included**

**(Cost-)effectiveness theme: Identification of studies via databases and registers**

Records identified from databases

(n= 2,186)

Records removed *before screening*:

Duplicate records removed (n= 1,073)

**Identification**

Records screened

(n = 1,113)

Records excluded (n= 1,053)

Reports sought for retrieval

(n= 60)

Reports not retrieved (n= 0)

**Screening**

Reports assessed for eligibility

(n= 60)

Reports excluded: (n= 25)

Wrong population (n= 9)

Wrong interventions (n= 2)

Wrong publication type (n= 7)

No relevant outcomes reported (n=7)

Studies included in review

(n= 35)

**Included**

**Implementation theme: Identification of studies via databases and registers**

Records identified from databases (n= 2,256)

Records removed *before screening*:

Duplicate records removed (n=857)

**Identification**

Records screened

(n = 1,399)

Records excluded (n= 1,143)

Reports sought for retrieval

(n=256)

Reports not retrieved (n= 2)

**Screening**

Reports excluded: (n= 221)

Wrong population (n=56)

Wrong publication type (n=86)

No implementation reported (n= 69)

No or wrong technology reported (n=8)

No report in English (n=2)

Reports assessed for eligibility

(n=254)

Studies included in review

(n= 33)

**Included**

**Ethics theme: Identification of studies via databases and registers**

Records identified form databases

(n= 1,578)

Records removed *before screening*:

Duplicate records removed (n= 363)

**Identification**

Records screened

(n = 1,215)

Records excluded (n= 1,189)

Reports sought for retrieval

(n= 26)

Reports not retrieved (n= 0)

**Screening**

Reports assessed for eligibility

(n= 26)

Reports excluded: (n= 12)

Wrong population (n= 2)

Wrong publication type (n= 2)

No ethics discussion (n= 8)

Studies included in review

(n= 14)

**Included**

Source: Page MJ, et al. BMJ 2021;372:n71. doi: 10.1136/bmj.n71.

This work is licensed under CC BY 4.0. To view a copy of this license, visit <https://creativecommons.org/licenses/by/4.0/>
